# Infectious toxicities associated with bispecific antibodies and CAR-T Cells in multiple myeloma: a systematic review

**DOI:** 10.1007/s00277-026-07140-8

**Published:** 2026-06-18

**Authors:** Magdalena Benda, Patrick Reimann, Wolfgang Willenbacher, Eberhard Gunsilius, Katja Weisel, Irene Strassl, Maria Theresa Krauth, Stefan Knop, Thomas Winder, Normann Steiner

**Affiliations:** 1 Department of Internal Medicine II, Feldkirch Academic Teaching Hospital, Feldkirch, Austria; 2 Faculty of Medical Sciences, Private University of the Principality of Liechtenstein, Triesen, Principality of Liechtenstein; 3 Molecular Biology Laboratory, Vorarlberg Institute for Vascular Investigation and Treatment, Dornbirn, Austria; 4 Department of Internal Medicine V, Comprehensive Cancer Center Innsbruck, Medical University of Innsbruck, Innsbruck, Austria; 5 Connect to Cure, Syndena GmbH, Innsbruck, Austria; 6 Department of Oncology, Hematology and Bone Marrow Transplantation with Section Pneumology, University Cancer Center Hamburg-Eppendorf, Hamburg, Germany; 7 Division of Hematology with Stem Cell Transplantation, Hemostaseology and Medical Oncology, Department of Internal Medicine I, Ordensklinikum Linz, Linz, Austria; 8 Medical Faculty, Johannes Kepler University Linz, Linz, Austria; 9 Department of Internal Medicine I, Division Hematology & Hemostaseology, Medical University of Vienna, Vienna, Austria; 10 Department of Internal Medicine 5, Hematology and Oncology, Nuremberg General Hospital, Paracelsus Medical University, Nuremberg, Germany; 11 University of Zurich, Zurich, Switzerland

**Keywords:** Multiple myeloma, CAR-T cells, Bispecific antibodies, Infectious complications, Immunosuppression

## Abstract

T-cell–redirecting therapies have transformed the treatment of relapsed/refractory multiple myeloma (RRMM) but are associated with substantial infection risk. We systematically reviewed infections after CAR T-cell therapy and bispecific antibodies (BsAbs), focusing on incidence, timing, pathogens, risk factors, and prevention. Following PRISMA guidelines, we searched PubMed through May 22, 2026, and included studies reporting infectious outcomes in RRMM patients treated with CAR-T cells or BsAbs. A total of 123 predominantly early-phase, non-comparative studies were analyzed. Across BCMA-directed CAR-T products and BsAbs, infections were frequent (any-grade incidence ~ 40–80%), with highest risk early after CAR-T infusion and during the first months of BsAb therapy. Grade ≥ 3 infections were common and exceeded 50% in several BsAb cohorts. Fatal infections were less frequent but occurred across both CAR-T and BsAb studies. In randomized CAR-T trials, grade 5 infections represented a substantial proportion of treatment-related deaths. Talquetamab showed lower infection rates (47–55%) but relevant mucocutaneous toxicity. Viral and bacterial pathogens predominated; CMV reactivation, invasive fungal infections, and Pneumocystis jirovecii pneumonia, particularly without prophylaxis, were also reported. Risk factors included neutropenia, corticosteroid/tocilizumab exposure, and hypogammaglobulinemia. Antiviral and Pneumocystis jirovecii pneumonia (PJP) prophylaxis and, most notably, immunoglobulin replacement reduced infections, though residual risk persisted. Real-world data suggest higher infection-related healthcare use and mortality with BsAbs versus CAR-T therapy. Extended dosing intervals may reduce infections, particularly severe infections, while preserving efficacy. Infectious toxicity remains a key limitation. Risk is particularly high with BCMA-targeted therapies and continuous BsAb administration. Tailored prophylaxis, early immunoglobulin replacement, and response-adapted treatment strategies, including extended dosing intervals, may improve safety and outcomes.

**Supplementary Information:**

The online version contains supplementary material available at https://doi.org/10.1007/s00277-026-07140-8.

## Introduction

The therapeutic landscape for multiple myeloma (MM) has transformed. Following the introduction of monoclonal antibodies, bispecific antibodies (BsAbs) and CAR-T cell therapies have ushered in T-cell–mediated immunotherapy and are central to relapsed or refractory multiple myeloma (RRMM).

While both induce deep responses, they also cause prolonged immune dysregulation, making infections a major driver of morbidity, mortality, and healthcare use. Persistent cytopenias, B-cell aplasia, hypogammaglobulinemia, and impairment of innate and adaptive immune compartments contribute to immunosuppression [1].

Despite growing clinical use, a systematic, infection-focused synthesis of incidence, timing, severity, risk factors, and preventive strategies remains limited and is essential for responsible resource utilization and patient selection. CAR-T cell therapy is resource-intensive and requires complex logistics, whereas BsAbs are administered continuously in most current regimens, potentially prolonging exposure-related infectious risk [2].

Direct comparative data are currently lacking. However, emerging real-world data suggest potential differences in infection profiles and toxicity severity [3].

This systematic review therefore focuses on infectious complications of CAR-T cells and BsAbs in RRMM, compares infection profiles across agents, and summarizes management strategies to inform patient selection, treatment planning, and supportive care.

## Methods

### Search methods and study selection

This review followed PRISMA 2020 guidelines [4]. PubMed/MEDLINE was searched through May 22, 2026.

Eligible studies included randomized, cohort, and real-world studies reporting infectious outcomes in MM patients treated with CAR-T cells or BsAbs. Reviews, meta-analyses, case reports, editorials, and studies without extractable infection data were excluded.

Screening and extraction were performed independently (MB, NS), with consensus resolution (TW).

The search strategy combined terms for disease (“multiple myeloma”), treatment (“CAR-T”, “bispecific antibody”, teclistamab, elranatamab, linvoseltamab, talquetamab, cevostamab, cilta-cel, ide-cel), and outcome (“infection”).

#### Inclusion criteria

- Adult patients with multiple myeloma.
- Reports on infectious toxicity, including incidence, timing, severity, pathogen spectrum, or infection-related mortality.
- Treatment with CAR-T cell therapy and/or BsAbs.

#### Exclusion criteria

- Malignancies other than multiple myeloma.
- Absence of reported infectious outcome.
- Treatment modalities other than CAR-T cells or BsAbs.

### Quality assessment and risk of bias

**Fig. 1:**
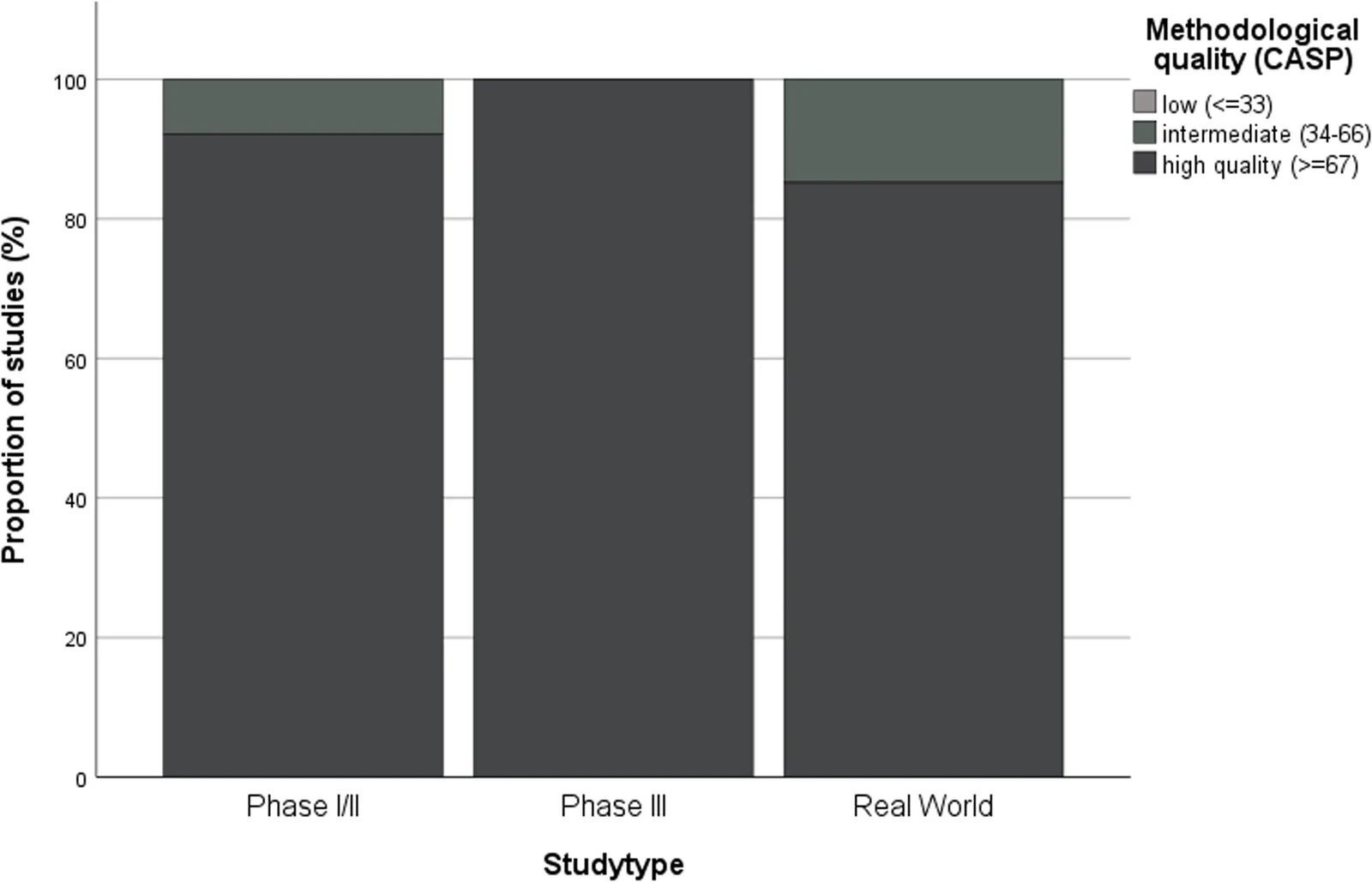
Methodological appraisal of included studies assessed by the Critical Appraisal Skills Programme (CASP). CASP-based methodological appraisal of included studies.Stacked bars display the proportion of studies within each study type (Phase I/II, Phase III, and real-world evidence), normalized to 100%. For descriptive visualization, CASP checklist items were summarized as the proportion of criteria fulfilled per study and categorized as intermediate (34–66%) or higher completeness (≥67%). No study met criteria for low completeness (<33%). CASP-based categorization was used for descriptive purposes only and did not inform study weighting or exclusion

Methodological quality was assessed using CASP checklists (RCT or cohort versions as appropriate) [5]. The proportion of fulfilled items was calculated as a percentage solely for visualization and categorized descriptively (34–66% intermediate; ≥67% high); results are shown in Fig. 1, with item-level details in Supplementary Table 1.

**Fig. 2:**
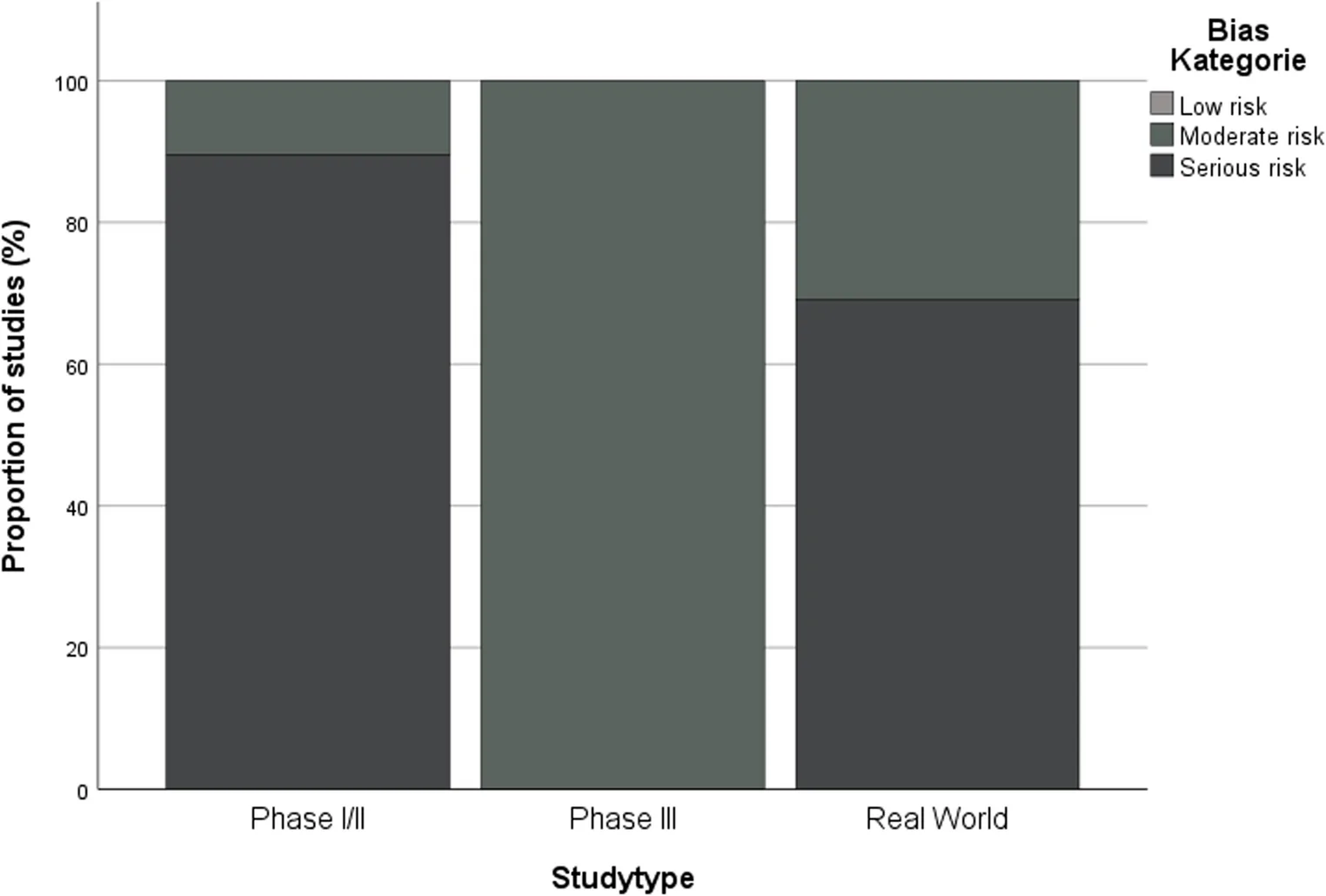
Overall risk of bias by study type. Distribution of overall risk of bias across study types. Bars represent 100%-normalized proportions of studies within each study type. Risk of bias was assessed using RoB 2 for randomized controlled trials and ROBINS-I for non-randomized and real-world studies; overall judgments were derived using the worst-domain principle

Risk of bias was evaluated using RoB 2 (randomized trials) and ROBINS-I (non-randomized studies). Overall judgments followed the worst-domain principle and are summarized in Fig. 2 and Supplementary Table 2 [6, 7].

### Data synthesis

Due to substantial heterogeneity, quantitative meta-analysis was not considered appropriate. Results are presented narratively by treatment modality and agent.

Language editing was performed using AI tools (DeepL, ChatGPT) and professional editing services (MDPI). The authors are solely responsible for its scientific content.

## Systematic review

**Fig. 3:**
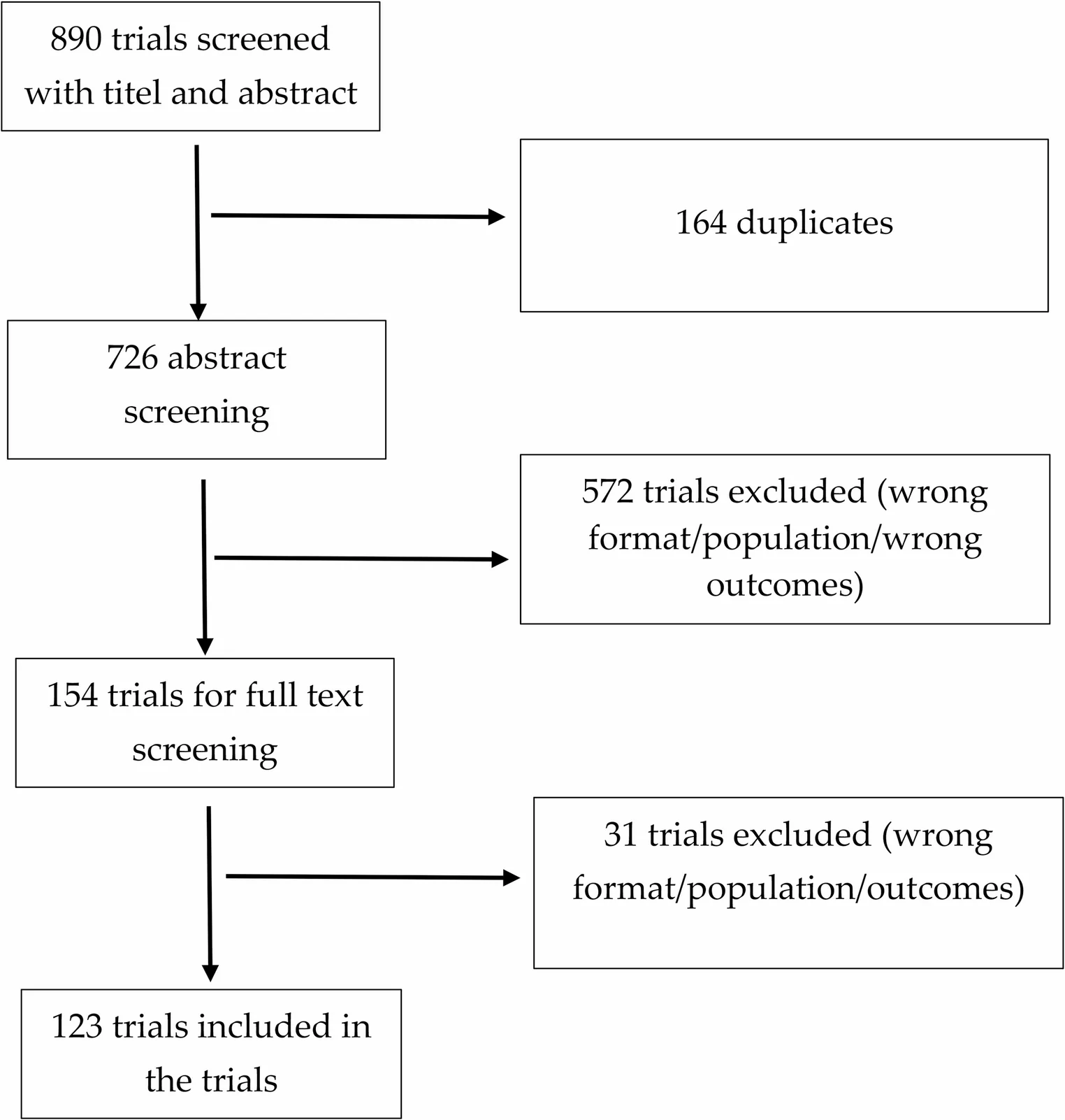
Flow-chart of Research. Flow diagram showing the study selection process from initial title and abstract screening to full-text assessment and final study inclusion

The review included 123 studies (Fig. 3). Lower CASP scores reflected non-comparative designs, small cohorts, short follow-up, and incomplete reporting of attrition and confounder control (Fig. 1; Supplementary Table 1). Overall risk of bias was often rated serious (Fig. 2), mainly due to confounding and selection bias inherent to early-phase, single-arm studies, while other domains were low to moderate, indicating generally robust conduct (Supplementary Table 2).

Cumulative infection incidence by treatment and type is presented in Table 1. Table 2 contextualizes risk by modality, setting, and cohort size. Supplementary Table 3 summarizes study characteristics, responses, key adverse events (CRS, ICANS), and infection prophylaxis/types; Supplementary Table 4 provides a summary and endpoints.

**Table 1:** Cumulative incidence of infections stratified by therapy and infection type

Any grade infection	Grade 3/ 4 (grade 5)	Bacterial infection	Other viral infection	CMV	PJP	HSV/VZV	Fungal infection
Teclistamab							
36.8–96.5%	19.2–54.1% (5%)	1.2–72.2%	41–45%	1.8–11.5%	2.7–3.6%	4.2%	2.8–6.7%
Elranatamab							
44-74.5%	39.8–41.7% (6.5%)	5.7–18.9%	43.5.	5.7–10.8%	3-4.9%	n.a.	7%
Linvoseltamab							
75.2%	47.9%	n.a.	6%	n.a.	4.3%	n.a.	5.2%
AMG 420							
33% (10%)	10%	n.a.	n.a.	n.a.	n.a.	n.a.	2%
Pavurutamab							
70.9%	30.8% (4.1%)	n.a.	26.7%	4.1%	1.2%	0.6%	4.7%
Alnuctamab							
64.2%	14.7% (1%)	n.a.	51.6%	4%	0%	n.a.	1%
Talquetamab							
55.8–73%	16.7–48% (1.5%)	4.5%-41%	59%.	1–11%	0%	n.a.	2-2.9%
Cevostamab							
42.5	18.8%	n.a.	n.a.	n.a.	n.a.	n.a.	n.a.
All bispecific antibodies							
27.4–100%	24-66.7% (4%)	12–56%	46–60%	9–47%	2.2–13%	3–6%	1–12%
Idecabtagene Vicleucel							
44-75.8%	12–23%	8–50%	16–47%	9.2–11%	0%	6–10%	1–5%
Ciltacabtagene autoleucel:							
85.4%	24-37.5% (4%)	11.1–43%	42%	n.a.	2%	18.8%	2.7–14%
All Car-T cells:							
30.6–61%	17.6–34%	2.4–42%	5–10%	5–20%	0%	7%	2–10%

Table 1 shows the cumulative incidence of infections across the studies of the individual approved treatments. The cumulative incidence of all infections is reported, with Grade 3/4 infections indicated in parentheses. Cytomegalovirus (CMV) infections, herpes simplex virus and varicella-zoster virus infections (HSV/VZV), as well as other viral infections, are listed. Additionally, Pneumocystis pneumonia and fungal infections are described. Only the percentage values are provided. Where available, the time window of infections (in days) or the frequency reported over the follow-up period in the studies (referred to as “overall”) is also included

**Table 2:** Study characteristics and infection-related outcomes by study design and recruitment period

Subject	Study design	Study	Pts.count	Recrutiment period	LOT	Infections	Hypo-IgG	NE
Teclistamab	Phase I/II	Moreau et al. N Engl J med 2022	51–200	2017–2021	> 3 LOT	76.4% (44.8%)	74.5%	70.9% (64.2%)
		Usmani, Garfall et al. 2021	51–200	2017–2021	> 3 LOT	60% (22%)	n.a.	65% (40%)
		Nooka, Rodriguez et al. 2024	51–200	2020–2021	> 3 LOT	80% (55.2%)	71.5%	71.5% (65.5%)
		Frerichs, Verkleij et al. 2024	51–200	2020–2021	> 3 LOT	(42.3%)	n.a.	n.a.
		Touzeau, Krishnan et al. 2024	≤ 50	2020–2023	> 3 LOT	70% (42.5%)	n.a.	n.a.
		Ishida, Kuroda et al. 2025	≤ 50	2021–2024	> 3 LOT	76.9% (19.2%)	84.6%	73.1%
		Cai, Xia et al. 2025	≤ 50	2021–2022	> 3 LOT	96.2%	92.3%	n.a.
	Phase III	Costa, Bahlis et al. 2025	> 200	2021–2023	> 1 LOT	96.5%	84.5%	75.6%
	Real-World	Dima, Davis et al. 2024	51–200	2022–2023	n.a.	36.8% (17%)	42%	47% (21%)
		Riedhammer, Bassermann et al. 2024	51–200	2022–2023	> 3 LOT	54% (26.8%)	n.a.	55.3% (38%)
		Kawasaki, Steele et al. 2024	≤ 50	2022–2023	> 3 LOT	25%	n.a.	22%
		Tan, Asoori et al. 2025	> 200	2022–2023	> 3 LOT	56.2% (22%)	69%	(39.5%)
		Perrot, Hulin et al. 2024	> 200	2022–2023	> 3 LOT	29.9%	n.a.	n.a.
		Mohan, Monge et al. 2024	51–200	2023–2023	> 3 LOT	40% (26%)	n.a.	n.a.
		Ariane, Stéphanie et al. 2025	≤ 50	2022–2023	n.a.	54.2%	50%	n.a.
		Razzo, Midha et al. 2026	> 200	2022–2024	> 3 LOT	42% (22%)	n.a.	n.a.
		Bros, Harel et al. 2025	≤ 50	2022–2023	> 3 LOT	n.a.	n.a.	n.a.
		Snyder, Atrash et al. 2025	51–200	2022–2024	> 3 LOT	Continuous: 59% (22%); Fixed: 75% (69%)	n.a.	n.a.
		Stork, Radoucha et al. 2025	51–200	2023–2025	> 3 LOT	76.7% 834.2%)	n.a.	52.1% (28.8%)
		Yi, Lee et al. 2025	< 50	2022–2023	> 3 LOT	47.6% (42.9%)	50%	73.8% (69%)
		Smits, Groen et al. 2026	51–200	2019–2024	> 3 LOT	90% (38%)	83%	n.a.
		Ssentongo, Guare et al. 2026	< 50	2023	> 3 LOT	57.9% (78.9%)	n.a.	n.a.
		Cheema, Shrestha et al. 2026	51–200	2022–2024	> 3 LOT	n.a.	n.a.	n.a.
Elranatamab	Phase I/II	Lesokhin, Tomasson et al. 2023	51–200	2021–2022	> 3 LOT	69.9% (39.8%)	75.5%	48.8%
		Bahlis, Costello et al. 2023	51–200	2017–2021	> 3 LOT	74.5% (26.8%)	n.a.	74.5%
		Tomasson, Iida et al. 2024	51–200	2021–2022	> 3 LOT	70.70%	n.a.	49.6%
		Iida, Ito et al. 2024	≤ 50	2021–2022	> 3 LOT	58.3%-75%	25–50%	75-83.3%
		Soltantabar, Hibma et al. 2026	> 200	2021–2022	> 3 LOT	41.7%grade ≥ 3 infections	n.a.	48.1% grade ≥ 3 neutropenia
	Real-World	Malard, Bobin et al. 2024	51–200	2022–2023	> 3 LOT	49% (24%)	n.a.	n.a.
		Portuguese, Davis et al. 2026	51–200	2023–2025	> 3 LOT	38% (57%)	n.a.	n.a.
AMG420	Phase I/II	Topp, Duell et al. 2020	≤ 50	2015–2019	> 3 LOT	33% (10%)	n.a.	n.a.
Pavurutamab	Phase I	Lee, Plattel et al. 2026	51–200	2017–2022	> 3 LOT	70.9% (30.8%)	n.a.	n.a.
Linvoseltamab	Phase I/II	Bumma, Richter et al. 2024	51–200	2019–2022	> 3 LOT	74.4% (25.9%)	n.a.	n.a.
	Phase I/II	Lee, Zonder et al. 2025	51–200	2019–2022	> 3 LOT	75%( 48%)	n.a.	43.6% (42.7%)
Alnuctamab	Phase I	Bar, Martin et al. 2026	51–200	2021–2024	> 3 LOT	64.2% (14.7%)	51.6%	53.7% (43.2%)
Talquetamab	Phase I/II	Chari, Minnema et al. 2022	> 200	2018–2021	> 3 LOT	n.a.	87%	67%
		Chari, Touzeau et al. 2025;	> 200	2018–2023	n.a.	n.a.	n.a.	34.9%
		Iida, Sunami et al. 2025	≤ 50	2021–2024	> 3 LOT	53.3%	6%	60%
		Schinke, Rodriguez-Otero et al. 2025	> 200	2018–2023	> 3 LOT	59–73% (16–28%, grade 5 1.5%)	32–49%	28–55% (22–53%)
	Real-World	Schinke, Dhakal et al. 2024	≤ 50	2024	n.a.	n.a.	n.a.	n.a.
		Frenking, Riedhammer et al. 2025	51–200	2023–2024	> 3 LOT	55.8% (16.7%)	50%	56%
		Dhakal, Akhtar et al. 2025	51–200	2022–2025	5 LOT	5% grade ≥ 3 infections after CAR-T	n.a.	n.a.
		Al Hadid, Szabo et al. 2025	51–200	2023–2025	6	27% (18%)	n.a.	n.a.
		Janakiram, Tan et al. 2026	51–200	2022–2025	6	51% (48%)	n.a.	36% (36%)
Cevostamab	Phase I/II	Trudel, Cohen et al. 2021	51–200	2020–2021	> 3 LOT	42.5%	45%	n.a.
	Phase I/II	Lesokhin, Richter et al. 2022	> 200	2021–2023	> 3 LOT	12%	n.a.	n.a.
Ide-Cel	Phase I/II	Munshi, Anderson et al. 2021;^10^	51–200	2017–2018	> 3 LOT	69% (22%)	21%	91% (89%)
		Lin, Raje et al. 2023	51–200	2016–2020	> 3 LOT	n.a.	75.8% (22.6%)	88.7%
		Minakata, Ishida et al. 2023	≤ 50	n.a.	> 3 LOT	56% (11%)	n.a.	100%
	Phase III	Ailawadhi, Arnulf et al. 2024	> 200	2017–2018	> 2 LOT	n.a.	24%	n.a.
	Real-World	Hashmi, Hansen et al. 2024	> 200	2021–2022	> 3 LOT	n.a.	n.a.	n.a.
		Hansen, Sidana et al. 2023	51–200	2021–2022	> 2 LOT	n.a.	34%	97%
		Kalariya, Hildebrandt et al. 2024;	51–200	2021–2022	> 3 LOT	< 65 y 17.2% ≥ 65 y 24.0%	n.a.	Day 30 41.4%
		Gaballa, Puglianini et al. 2025;	≤ 50	prior to 2025	> 3 LOT	50%	n.a.	n.a.
		Ferreri, Hildebrandt et al. 2023;	> 200	2021–2022	> 3 LOT	Prior BCMA 82% no prior BCMA 39%	n.a.	n.a.
		Dima, Rashid et al. 2024;	51–200	up to 2023	> 3 LOT	69% (22%)	n.a.	41%
		Sidana, Ahmed et al. 2025;	> 200	n.a.	> 3 LOT	45%	n.a.	n.a.
		Trando, Ghamsari et al. 2024;	≤ 50	2021–2023	> 3 LOT	44% (12%)	96%	n.a.
		Logue, Peres et al. 2022	51–200	2021	> 3 LOT	54% (23%)	n.a.	n.a.
		Kikuchi, Watanabe et al.;	51–200	2022–2024	n.a.	n.a.	n.a.	n.a.
		Akhtar, Oloyede et al. 2025;	> 200	n.a.	n.a.	frail 49.6% no frail 40.9%	n.a.	n.a.
		Caillot, Sleiman et al. 204	≤ 50	2021–2023	> 3 LOT	79% (33%)	n.a.	n.a.
		Sanoyan, Seipel et al. 2023	≤ 50	2022	n.a.	53%	85%	100%
Cilta-Cel	Phase I/II	Berdeja, Madduri et al. 2021;	51–200	2018–2019	> 3 LOT	58% (20%)	n.a.	95.9%
		Mi, Zhao et al. 2023	≤ 50	2021–2022	n.a.	85.4%	70%	97.9%
		Ri, Suzuki et al. 2022	≤ 50	2019–2021	> 3 LOT	11.1%	n.a.	88.9%
		Htut, Dhakal et al. 2023	≤ 50	2018–2020	> 3 LOT	57.1% (20%)	65%	100%
	Phase III	San-Miguel, Dhakal et al.2023 Einsele, San-Miguel et al. 2026	> 200	2020–2021	> 1 LOT	62% (26.9%)	90.9%	88.9%
	Real-World	Sidana, Patel et al.2025	> 200	2022	> 3 LOT	47% (21.7%)	n.a.	n.a.
		Ellithi, Elsallab et al. 2025	> 200	2021–2023	n.a.	n.a.	n.a.	n.a.
		Dima, Logue et al. 2026	51–200	2022–2023	> 3 LOT	49% (32%)	n.a.	n.a.
		Hansen, Dima et al. 2026	> 200	2022–2023	> 3 LOT	47%	n.a.	At day 30 15%
All BsABs	Phase I/II	Cohen, Magen et al. 2025	51–200	2020–2023	n.a.	89% (64%)	n.a.	73%
		Kumar, Mateos et al. 2026	51–200	n.a.	> 3 LOT	31% grade 3 /4	n.a.	n.a.
	Real-World	Jourdes, Cellerin et al. 2024	> 200	2020–2023	> 3LOT	70% (53%)	61%	n.a.
		Li, Zhang et al. 2025	≤ 50	n.a.	n.a.	n.a.	n.a.	n.a.
		Mohan, Szabo et al. 2025	> 200	2017–2023	> 3LOT	73% (53%)	n.a.	n.a.
		Lancman, Parsa et al. 2023	≤ 50	2019–2022	> 3LOT	100%	100%	73%
		Uttervall, Tätting et al. 2024	51–200	2021–2023	> 3LOT	53%	n.a.	43%
		Sim, Longhitano et al. 2023	≤ 50	2018–2022	> 3LOT	90% (41%)	87%	n.a.
		Contejean, Janssen et al. 2023	> 200	2018–2023	n.a.	27.4% (24%)	n.a.	n.a.
		Kowalski, Lykon et al. 2025	51–200	2022–2024	> 3LOT	47% (17.6%)	80%	n.a.
		Choi, Buyn et al. 2024	51–200	2021–2023	n.a.	81%	81%	n.a.
		Park, Jang et al. 2025	51–200	2021–2024	> 3LOT	66%	n.a.	n.a.
		Banerjee, Marsal et al. 2021	≤ 50	2017–2020	n.a.	severe 6%	n.a.	n.a.
		Cani, Scott et al. 2025	51–200	2022–2024	> 3 LOT	46%, (25%)	39–62%	58–63%
		Wang, Li et al. 2026	> 200	Up to 2025	> 2 LOT	n.a.	n.a.	n.a.
		Jelinek, Zihala et al. 2026	51–200	2017–2025	> 2 LOT	Anti-BCMA: 79% (33%); Anti-GPRC5D: 54% (34%)	n.a.	n.a.
		Piron, Rodrigo et al. 2025	51–200	2020–2024	> 3 LOT	84.8%	n.a.	n.a.
		Bilbao, Anson et al. 2025	51–200	2021–2025	> 3 LOT	n.a.	n.a.	n.a.
		Tan, Usmani et al. 2026	> 200	2022–2025	> 3 LOT	n.a.	66%	n.a.
All BsABs and CAR-T cell therapies	Real-World	Nath, Shekarkhand et al. 2024;	> 200	CAR-T: 2017–2023; bsAB: 2020–2023; ADC 2018–2023	> 3 LOT	83.5%	n.a.	n.a.
		Howard, Concepcion et al. 2024	> 200	2021–2023	> 3 LOT	CAR-T20%, bsAB24%	n.a.	n.a.
		6Hammons, Haider et al. 2024;	> 200	n.a.	> 3 LOT	CAR-T50%, bsAB44%	n.a.	n.a.
		Lesokhin, Nath et al. 2024	> 200	CAR-T: 2017–2023; bsAb 2020–2023; ADC 2018–2023	> 3 LOT	CAR-T60%, bsAB49%	n.a.	n.a.
		Mersi, Burow et al. 2026	51–200	2016–2023	> 3 LOT	CAR-T67% (19%) bsAB-BCMA:85%(19%) bsAB-GPRC5D:81%(8%)	n.a.	n.a.
All CAR-T cell therapies	Phase I/II	Xu, Wang et al.	51–200	2016–2022	n.a.	n.a.	n.a.	n.a.
		Oliver-Caldés, González-Calle et al. 2023	51–200	2020–2021	> 3 LOT	67%	n.a.	n.a.
		Mailankody, Matous et al. 2023	≤ 50	2022	n.a.	53.5% (23.3%)	n.a.	n.a.
		Ravi, Richard et al. 2025	51–200	2020–2022	> 3 LOT	32% (5%)	n.a.	n.a.
		Zhao, Wang et al. 2022	51–200	2016–2017	n.a.	n.a.	n.a.	n.a.
		Xia, Sun et al. 2025	≤ 50	2021–2024	> 3 LOT	51% (22%)	27%	n.a.
		Yan, Du et al. 2026	≤ 50	2023–2024	0 LOT	30.6% (19.4%)	n.a.	88.9%
	Real-World	Hansen, Peres et al. 2025	> 200	up to 2021	> 2 LOT	IdeCel (I) 58% (28%), CiltaCel (C) 61% (34%)	I: 67% C 79%	n.a.
		Merz, Albici et al. 2025	> 200	2021–2024	> 3 LOT	n.a.	n.a.	n.a.
		Wesson, Dima et al. 2024	51–200	2021–2023		40%(20%)	50%	n.a.
		Wang, Cao et al. 2022	51–200	2017–2020	> 3 LOT	45%(27%)	45%	n.a.
		Merz, Gagelmann et al. 2025	> 200	n.a.	n.a.	47.1%(17.6%)	79,8%	n.a.
		Portuguese, Liang et al. 2025;	51–200	2021–2024	> 3 LOT	n.a.	n.a.	n.a.
		Rejeski, Hansen et al. 2023	51–200	2021–2022	> 3 LOT	39% (20%)	n.a.	n.a.
		Hill, Martens et al. 2024	51–200	2021	n.a.	29%	n.a.	n.a.
		Little, Tandon et al. 2023	51–200	2016–2022	> 3 LOT	37%	70%	n.a.
		Richardson, Schütte et al.2025	51–200	n.a.	> 3 LOT	71%	n.a.	n.a.
		Frenking, Zhou et al. 2024	51–200	up to 2023	> 3 LOT	severe 13%	n.a.	n.a.
		Reyes, Huang et al. 2023	51–200	2017–2022	> 3 LOT	36 vs. 52%	82%	n.a.
		Wang, Lii et al. 2021	≤ 50	2017–2020	> 3 LOT	57,5%	53%	n.a.
		Thiruvengadam, Sheng et al. 2022	51–200	2018–2020	> 3 LOT	53%	76%	n.a.
		Josyula, Pont et al. 2022	≤ 50	2018–2020	> 3 LOT	53%	88%	n.a.
		Little, Pena et al. 2025	> 200	2016–2023	> 3 LOT	n.a.	n.a.	n.a.
		Cohen, Lebel et al. 2026	51–200	2021–2023	> 3 LOT	n.a.	n.a.	n.a.
		Habib, Ahmed et al. 2026	> 200	2021–2024	> 3 LOT	severe 44%	n.a.	84%
		Liu, Zhang et al. 2026	51–200	2017–2024	> 3 LOT	Severe 29.9%	80%	n.a.

This table summarizes study design, recruitment period, and key infection-related outcomes from clinical trials and real-world studies evaluating bispecific antibodies (BsABs) and CAR-T cell therapies in relapsed or refractory multiple myeloma. Reported variables include patient numbers, number of prior lines of therapy (LOT), incidence of infections, hypogammaglobulinemia (Hypo-IgG), and neutropenia (NE). Infection rates are reported as the proportion of patients experiencing at least one infection; values in parentheses denote grade ≥ 3 or severe infections, where specified in the original publications. Data are presented as reported by the study authors; missing information is indicated as “n.a.”. Differences in study design, recruitment period, follow-up duration, and outcome definitions limit direct comparability across studies

Abbreviations: BsAB, bispecific antibody; CAR-T, chimeric antigen receptor T cell; LOT, lines of therapy; Hypo-IgG, hypogammaglobulinemia; NE, neutropenia; n.a., not available

### Teclistamab

We included seven early-phase trial reports, one phase-III trial and fifteen real-world cohort studies [8–29].

An extended safety analysis of MajesTEC-1 reported infections in 80% of patients, predominantly involving the respiratory tract (19.4%), with an infection-related mortality rate of 12.7%. Median onset was 1.7 months; although most events occurred within 30 days, risk persisted over time. Pathogens included herpesviruses (4.2%), CMV reactivation (1.2%), PJP (4.2%), and fungal infections (5.5%) [10]. The phase III MajesTEC-3 trial confirmed the substantial infectious burden associated with BCMA-directed bispecific antibody therapy in earlier treatment lines, with infections occurring in 96.5% of patients, grade 3–4 infections in 54.1%, and fatal infections in 4.6%. Most infection-related deaths occurred within the first six months of therapy, while the incidence of new severe infections declined after transition to monthly dosing and reinforced immunoglobulin replacement strategies [30].

Real-world data confirmed infection rates of 42–60%, with serious infections in 22%-42.9%, 13% discontinuation and 30% hospitalization; infections were predominantly viral (50%) and bacterial (45%), whereas opportunistic infections occurred less frequently, including fungal infections (5%) and CMV reactivation (22%) [9, 13, 15, 19, 22, 26, 29]. Infections remain the leading cause of treatment discontinuation in real world setting [28].

Cohorts were consistently heavily pretreated but heterogeneous in recruitment and design; Prospective trials applied Common Terminology Criteria for Adverse Events (CTCAE) reporting, whereas real-world studies were retrospective, contributing to differences in captured low-grade events. Antiviral prophylaxis (HSV/VZV) became standard alongside PJP prophylaxis (initially 83% in the dose-finding trial), with antibacterial and antifungal prophylaxis during neutropenia [9, 16]. Intravenous immunoglobulin (IVIG) use increased from 11% to 62% and was associated with fewer severe infections (grade 3/4 at 6 months: 5.3% vs. 54.8%) [12, 15, 18, 20]. The lower infection rates in the real-world cohort were partly attributed to treatment outside the peak COVID-19 pandemic period, but also to increased IVIG use in routine clinical practice [22]. Increasingly early and systematic IVIG replacement has been adopted in the real-world setting and was associated with reduced annualized rates of both all-grade and severe infections [23, 27]. However, limitations in IVIG availability have been reported [21]. Real-world analyses of fixed-duration teclistamab showed that infections may persist despite treatment discontinuation [24]. Similarly, reduced-frequency dosing (every 2–4 weeks instead of weekly) was associated with comparable PFS and lower rates of neutropenia, although infection risk remained clinically relevant [25]. More recent analyses also reported reductions in annualized all-grade and severe infection rates following dose de-escalation [27]. Nevertheless, the interpretation of dose reduction or discontinuation strategies remains limited by small cohort sizes and relatively short follow-up periods.

Furthermore, BCMA-directed therapy induces profound B-cell depletion and hypogammaglobulinemia, resulting in impaired vaccine responses; current practice includes baseline HBV, HCV, and HIV screening, CMV monitoring, and up-to-date vaccination against influenza, VZV, pneumococci, and SARS-CoV-2 [10, 11].

### Elranatamab

This review comprises five prospective early-phase trials and two real-world analysis. Cohort sizes were comparable (51–200 patients in 4 of 5 studies), with longer follow-up in the real-world cohort. All patients were heavily pretreated [31–37].

High rates of infection (74.5%) and hypogammaglobulinemia (75.5%) were reported in clinical trials with a 49% infection incidence in the real-world setting. These differences likely reflect study design (prospective vs. retrospective) and pandemic-related follow-up [32, 35]. Grade ≥ 3 infections occurred in 41.7%. While cytopenias mainly occurred during the initial treatment cycles, severe infections were distributed across later treatment periods [36].

Prophylactic strategies varied widely: while > 80% received antivirals and ~ 50% PJP prophylaxis, antifungal and antibacterial strategies were rarely used [31]. The protective effect of immunoglobulin supplementation was described as the most apparent factor associated with a reduced risk of grade ≥ 3 infections, despite heterogeneous implementations in clinical trials. In real-world setting immunoglobulin supplementation was administered to 46% of patients and was associated with improved infection-free survival [37].

### Linvoseltamab

The phase I/II study reported infections in 74.4%. Three infection-related deaths and 9.4% treatment discontinuation were reported. Hypogammaglobulinemia occurred in 64.1% [38]. The updated analyses described grade ≥ 3 infection in 48%. Opportunistic infections (11%), including CMV reactivation/infection (5%) and PJP occurred. Infection incidence decreased after 6 months of treatment, coinciding with transition to less frequent Q4W dosing in responding patients [39].

### AMG 420

One single-arm, non-randomized phase I trial evaluated the BCMA-targeted antibody AMG. Severe adverse events occurred in 48% of patients, including 14 cases of infection and polyneuropathy. Due to the short half-life, daily infusion was necessary [40].

### Pavurutamab

One single-arm phase 1 study evaluating the BCMA-targeted BsAb pavurutamab in 172 heavily pretreated patients with relapsed/refractory multiple myeloma reported an ORR of 65.8%, with infections occurring in 70.9% of patients, including grade 3–4 infections in 30.8% and fatal infections in 4.1%. Opportunistic infections were reported in 12.2% of patients [41].

### Alnuctamab

The first-in-human phase I study of the BCMA-directed BsAb alnuctamab demonstrated meaningful clinical activity (ORR 58.9%) with frequent but predominantly low-grade infections (64.2%), while grade ≥ 3 infections occurred in 14.7% of patients. Opportunistic infections were uncommon and included asymptomatic CMV reactivation (4%) and a single case of fungal esophagitis [42].

### Talquetamab

A total of nine multicenter non-randomized studies investigating talquetamab were identified: four open-label clinical phase I/II trials and five retrospective real-world cohort studies [43–50].

Infections occurred in 47% of patients treated with talquetamab, with opportunistic infections reported in approximately 3%, including CMV reactivation and invasive fungal sepsis. Hypogammaglobulinemia occurred in 87% [43, 44]. An analysis of MonumenTAL-1 focusing on infections and humoral immunity showed that most severe infections occurred within the first 100 days of treatment, followed by rapid recovery of neutrophil counts and preservation of B-cell levels, with polyclonal IgG levels increasing over time [47, 48].

In the real-world setting, infections occurred in 51-55.8% of patients, with 48% of infected patients requiring hospitalization. CMV reactivation occurred in 11% [46, 50]. No infection-related deaths occurred [49].Differences likely reflect recruitment periods and more detailed prospective monitoring in trials. Results from Iida et al. differed from other trials, likely due to small sample size and limited interpretability [45]. The target GPRC5D was associated with additional toxicities affecting the skin, nails, and oral mucosa [43]. Local care measures involving cortisone and nystatin are being considered as a way of preventing local inflammation [47]. Haematotoxicity (36–67%) was rarely severe [43–46]. Talquetamab bridging to CAR-T therapy in 134 real-world patients achieved a 71% response rate, while grade ≥ 3 infections after CAR-T occurred in 5% of patients .

### Cevostamab

Cevostamab, the first FcRH5-directed BsAb, has been evaluated in two single-arm phase I clinical trials. Infections occurred in 49–53.9% of patients, including severe infections in 22.2%, with predominantly bacterial and rare fungal etiologies [51]. Hypogammaglobulinemia was observed in nearly half of patients [51, 52].

### Across bispecific antibody therapies and their combinations

Nineteen studies (seventeen real-world) evaluated infection risk under BsAb therapy [53–72]. Populations were heavily pretreated and older (median age 67; ECOG ≥ 1 in 77.6%) than in clinical trials, addressing COVID-19, infection timing, immunoglobulin substitution, and infection patterns.

Jourdes et al. reported 234 infections (53% grade ≥ 3) in 229 patients receiving approved BsAbs, with higher rates under BCMA- versus GPRC5D-directed agents [55]. BCMA-directed bispecific antibodies, unlike GPRC5D-directed bispecific antibodies, led to profound and persistent depletion of mature B cells, B-cell precursors, and normal plasma cells [68]. Between the first two BCMA-targeting bispecific antibodies, elranatamab and teclistamab, real-world data did not demonstrate significant differences in infection rates or infection severity after propensity score matching [67]. Most infections occurred within the first 3–6 months, but cumulative incidence increased to 67–72% by month 18, a pattern consistent with Hammons et al. (288 infections/225 patients), who observed PJP exclusively without prophylaxis [63, 64, 73]. Despite routine antiviral and PJP prophylaxis, infections—predominantly viral and bacterial—remained frequent [58–60]. A real-world analysis identified a relatively high incidence of Campylobacter spp. infections in patients receiving BCMA- and GPRC5D-directed bispecific antibodies [70]. Infection-related hospitalization was reported in 41% of cases, with treatment discontinuation due to infections in 7% and infection-related mortality in 4% [66]. In a real-world analysis, 34% of patients died within the first 12 months after initiation of bispecific antibody therapy. Progressive disease was the leading cause of death, followed by infections, which accounted for 13% of early mortality events [71].

Hypogammaglobulinaemia was common (40–100%), particularly with BCMA-directed bispecific antibodies, resulting in a higher need for immunoglobulin substitution. Immunoglobulin substitution reduced subsequent infections. Preemptive immunoglobulin substitution in patients receiving BCMA-directed bispecific antibodies was associated with a 66% reduction in all-grade infections and a 76% reduction in grade ≥ 3 infections, while also significantly delaying the onset of infectious events [69]. Primary IVIG prophylaxis was associated with improved PFS and OS, whereas corticosteroids, lymphopenia, and tocilizumab exposure increased infection risk; prophylactic tocilizumab was safe Next to IVIG initiation, reduced bispecific antibody dosing frequency was also associated with a lower incidence of infections [55, 57, 65, 66, 74]. Severe COVID-19 was linked to cytokine-mediated inflammation (leukemia inhibitory factor = LIF, JAK–STAT activation) [72].

Talquetamab–teclistamab combination therapy achieved an ORR of 80%, including substantial activity in extramedullary myeloma, but was associated with considerable toxicity, including 64% grade 3/4 infections [53]. In the phase II study restricted to extramedullary myeloma, grade 3/4 infections occurred in 31% of patients, with five infection-related deaths reported [54].

### Ide-Cel

Seventeen studies (three early-phase, one phase III, 13 real-world) evaluated ide-cel in heavily pretreated cohorts [75–91].

In phase I, neutropenia (88.7%) and infections (75.8%) were frequent; similar safety profiles were observed in phase II/III trials, with infections in up to 69% (grade 3/4: 22%). Antimicrobials, immunoglobulins, and granulocyte colony-stimulating factor (G-CSF) were commonly used [75, 91].

Real-world infection rates ranged from ~ 50% to 79% (overall ~ 53%), predominantly viral and bacterial. Variability reflected differences in patient characteristics, study design, supportive care, and infection definitions, including capture of mild events (e.g., BK virus) [84, 89].

Neutropenia (88%) and infections (34%) were reported in one cohort [76]. CMV reactivation occurred in up to 47.4%, including CMV rhinitis [85, 87]. Frailty—rather than chronological age—was associated with higher toxicity, including infections and ICANS; prior BCMA therapy did not increase infection risk [88].

A detailed real-world analysis reported infections in 54% (grade 3/4: 23%), clustering within 30 days; 50% of early infections were severe and mainly bacterial (68%; pneumonia, C. difficile colitis, soft tissue). Beyond day 30, infections were more heterogeneous (50% bacterial, 42% viral—primarily SARS-CoV-2) and less severe (grade 3/4: 13%). A longer interval between bridging therapy and lymphodepletion (29 vs. 15 days) was the only significant predictor of infection; tocilizumab, corticosteroids, and IVIG were not associated with increased risk [86].

By day 90, loss of preexisting immunity was observed in a subset (HSV 19%, VZV 16%, CMV 7%), correlating with low CD4 counts (median 90/µL); pneumococcal immunity declined in 32%. Supportive care was intensive: G-CSF in 88% (median start day 9; duration 29 days), autologous stem cell boost in 8%, transfusions in 65% within 100 days, and TPO agonists in 21% (limited efficacy; 82% remained thrombocytopenic). IVIG was used in 13% (median start day 39; ongoing 11%) [86].

### Cilta-Cel

Ten studies evaluated cilta-cel (four prospective early-phase single-arm trials, four large retrospective real-world cohort > 200 patients, and two publications of the randomized phase III trial). Except for the phase III study, cohorts were heavily pretreated [92–101].

Across phase I–III trials, infections occurred in 62–85.4%, alongside neutropenia (97.9%), CRS (76.1–97.9%), ICANS (4.5%), and hypogammaglobulinemia (70–91%). Three infection-related deaths were reported in phase II. Respiratory infections predominated; herpes zoster (18.8%) and HBV (1.1%) were also observed [92, 93, 95]. The updated randomized phase III CARTITUDE-4 trial further reported detailed infection grading under cilta-cel, with grade 1–2 infections occurring in 35% of patients, whereas clinically more relevant grade 3–4 infections requiring hospitalization occurred in 24% and < 1%, respectively. Grade 5 infections occurred in 4% of patients, and four of six treatment-related deaths were infection-related, predominantly within the first year after treatment initiation [99].

In real-world data, infection incidence was 47–49%. Patients were mainly recruited in 2022, with retrospective data collection and center-specific supportive care. Grade 3/4 infections occurred in 32% and were primarily bacterial (41%) and viral (47%), with few fungal events (3%). Infection was the leading cause of treatment-related mortality [94, 100, 101]. Prior allogeneic stem cell transplantation was not associated with an increased infection risk. Rare infections (e.g., Haemophilus influenzae, CMV) were reported post-marketing FAERS analyses [98]. Comprehensive prophylaxis—including antiviral, antibacterial, antifungal, and PJP prophylaxis—is recommended [96].

### Across CAR-T cell therapies and combinations

Twenty-one studies evaluated approved BCMA CAR-T cells (ide-cel, cilta-cel) seven single-arm trials and nineteen real-world analyses. One phase II study assessed the anti-GPRC5D CAR-T therapy. Most cohorts were heavily pretreated, with infection rates ranging from 32.5% to 67% (recruitment 2017–2024). Heterogeneity reflected differences in follow-up, patient populations and analytical focus [102–127].

No randomized head-to-head comparison of CAR-T products has been published; however, real-world analyses suggest higher response rates and longer PFS/OS with Cilta-Cel versus ide-cel, accompanied by higher infection rates, longer hospitalization, and increased steroid use [103]. Deep remission correlated with durable outcomes, and no safety differences were observed between patients aged ≥ 70 and < 70 years [102, 120].

Across products, efficacy was high [104]. Cytopenias occurred in 44% (long-term sequelae: 1.6%); neutropenia and infections were reported in 32–33%. Early mortality was low (1.1%), mainly due to hemophagocytic lymphohistiocytosis and infections, with an additional 1.2% by month three, exclusively infection-related. Approximately 25% required temporary relocation to the clinical center for monitoring [106].

Within one year, infections occurred in 53–71% (53% viral, 40% bacterial, 6% fungal), predominantly within the first 100 days; severe infections clustered in the first 28 days [116, 117, 121]. The 1-year cumulative incidence of invasive fungal disease was low (1.7%) and was largely restricted to patients receiving intensive immunosuppression for severe CAR T-cell–related toxicities [124]. CMV reactivation occurred in 20% and EBV reactivation in 8% of patients following anti-BCMA CAR T-cell therapy [125].

Hypogammaglobulinemia occurred frequently (80%) and was linked with an increased mortality following BCMA CAR-T therapy [127]. Persistent hypogammaglobulinemia at one year (IgG 65%, IgM 73%, IgA 34%) was associated with infection risk and long-term follow-up revealed sustained B-cell aplasia and herpes zoster reactivation [107, 116]. Extramedullary disease correlated with higher CRS, ICANS, infections, and treatment-related morbidity, with lower CR rates and shorter PFS/OS despite similar ORR [108]. Similarly, patients with renal impairment experienced increased toxicity, particularly higher rates of ICANS and severe infections requiring hospitalization (44% vs. 20%) [126].

The CAR-HEMATOTOX score stratified risk in 113 patients: high-risk patients (HT > 1) had more severe neutropenia (80% vs. 21%), severe infections (40% vs. 5%), and non-relapse mortality (13% vs. 2%), alongside inferior efficacy (≥ VGPR 44% vs. 70%), shorter PFS (5 vs. 15 months), prolonged neutropenia (9 vs. 3 days), and higher infection-related mortality [109]. The EASIX score predicted late hematotoxicity and severe late infections, ICHAT, and ICANS [119].

Post–CAR-T SARS-CoV-2 mRNA vaccination induced robust immune responses with few severe adverse events [115].

Long-term follow-up studies of BCMA and non-BCMA CAR-T products report durable efficacy with manageable toxicity. LCAR-B38M showed high ORR (88%) with low early, mainly viral infections, while 5-year data confirmed sustained OS (49%) [105, 114]. The Spanish ARI0002h achieved an ORR of 100% but with high rates of CRS and hematologic toxicity (67%) [110]. The allogeneic product ALLO-715 demonstrated moderate efficacy (ORR 56%), frequent but manageable infections (53.5%), and no graft-versus-host disease (GVHD) [111]. A next-generation BCMA CAR-T manufactured via the NEX T^®^ platform achieved a 95% ORR with few infections (32%, grade 3/4: 5%) [112]. An anti-GPRC5D CAR-T in phase 2 showed an ORR of 84% with manageable infections (51%) and cytopenias (78%) [118]. More recently, frontline BCMA-directed CAR-T therapy has been investigated in transplant-ineligible patients with newly diagnosed multiple myeloma and demonstrated deep, durable responses while maintaining a manageable safety profile. Infections occurred in 30.6% of patients, including 19.4% grade ≥ 3 infections, and no treatment-related deaths were reported [123].

### Across CAR-T Cells and Bispecific Antibodies therapies and combinations

Five large retrospective studies compared BCMA CAR-T, BsAbs, and antibody-drug conjugates (ADCs) [1, 3, 128–130]. Differences in recruitment periods (CAR-T 2017–2023; ADC from 2018; BsAbs from 2020), follow-up, and endpoints likely explain variability in reported infection rates (e.g. CAR-T 20–60%). Howard et al. assessed unscheduled health care interactions in responders over 125 days, whereas Lesokhin et al. analyzed CTCAE-graded infections with follow-up up to one year. Despite heterogeneity, these real-world data provide comparative insights not yet captured in clinical trials [1, 129]. 

Available real-world data suggests a higher burden of infections with BsAbs (99 events; grade ≥ 3 in 40%), followed by CAR-T therapy (115 events; grade ≥ 3 in 27%), with the lowest rates observed for ADCs. In reported comparative cohorts, infection-related deaths were observed predominantly in patients receiving BsAbs. No grade 4/5 infections were reported in the available CAR-T cohorts [129]. More recent real-world analyses have documented fatal infections in 8.6% of CAR-T recipients, all occurring within three months of infusion and associated with severe neutropenia [130]. Infection risk was further amplified by hypogammaglobulinemia and neutropenia, particularly in the BsAbs cohort. CAR-T infections clustered within 100 days (79%), whereas 50% of severe BsAbs infections occurred after day 100, indicating sustained risk. Median time to first infection was 2.5 months (CAR-T) versus 3.1 months (BsAbs); viral and bacterial pathogens predominated, while fungal/opportunistic infections were rare but more frequent with BsAbs [3, 129]. Beyond infection incidence and severity, recent real-world data indicate a greater cumulative infectious burden with BCMA-directed compared with GPRC5D-directed bispecific antibodies. Patients receiving BCMA-targeted BsAbs experienced prolonged periods of infection-related symptoms during treatment, with more than 80% of infections being low grade but often recurrent and persistent, resulting in a substantial impact on quality of life and overall treatment burden [130].

In a cohort of 46 patients, BsAbs were associated with 1.8-fold more unscheduled healthcare interactions, mainly due to mild respiratory infections; emergency visits and inpatient days were comparable. After allogeneic transplantation, CAR-T achieved higher ORR than BsAbs (92% vs. 44%) with similar infection rates and no increased GVHD [131].

## Discussion

This systematic review of 123 studies evaluates infectious complications in RRMM treated with CAR-T cells or BsAbs. Despite predominantly early-phase and heterogeneous evidence, a consistent pattern emerges: continuously administered BsAbs appear to be associated with a cumulative infectious burden, whereas CAR-T therapy is characterized by a more pronounced early infection peak. Infection risk—driven by cytopenias, hypogammaglobulinemia, and CRS/ICANS-related immunosuppression—remains a major cause of hospitalization and non-relapse mortality [3].

BCMA-directed BsAbs are associated with higher infection rates across studies, whereas talquetamab shows lower infection rates but notable mucocutaneous toxicity [43]. Infection risk appears to peak within the first six months and may persist, reflecting sustained B-cell depletion, hypogammaglobulinemia, neutropenia, and immunosuppression related to CRS/ICANS management [9, 15, 19, 53, 55]. Recent real-world studies suggest that immune dysfunction and infectious susceptibility may persist despite treatment discontinuation. However, currently available follow-up remains relatively short, and longer-term studies are needed to define the duration of immune impairment and infectious risk. Dose de-escalation and interval-extension strategies have been associated with lower rates of infections, particularly grade ≥ 3 infections, while maintaining clinical efficacy in selected patients. Controlled studies with longer follow-up are needed to confirm these preliminary observations [24, 25, 30]. Viral and bacterial infections predominate, although opportunistic infections occur [10, 12, 60, 61, 73]. The burden of severe infections should not be underestimated, as grade ≥ 3 infections frequently require hospitalization and are therefore associated with substantial impairment in quality of life [30, 55, 99, 130]. This appears particularly relevant for BCMA-directed bispecific antibodies. In addition, the duration and cumulative burden of infectious complications should be considered. Recent real-world data suggest that patients receiving BCMA-directed bispecific antibodies may spend a greater proportion of treatment time with infection-related symptoms compared with patients receiving GPRC5D-directed therapies. However, infection duration is rarely reported in current studies despite its likely impact on quality of life and healthcare utilization [130].

Antiviral and Pneumocystis prophylaxis are standard until immune reconstitution, while antibacterial and antifungal prophylaxis remain individualized [12, 83]. Although vaccine responses are frequently attenuated, vaccination remains a key component of infection prevention and should be implemented whenever feasible [11, 15, 19]. Across studies, the most consistent benefit is observed with pre-emptive and sustained immunoglobulin replacement targeting IgG levels > 400 mg/dL, which was associated with reduced severe infections and improved survival [21, 57, 58].

**Fig. 4:**
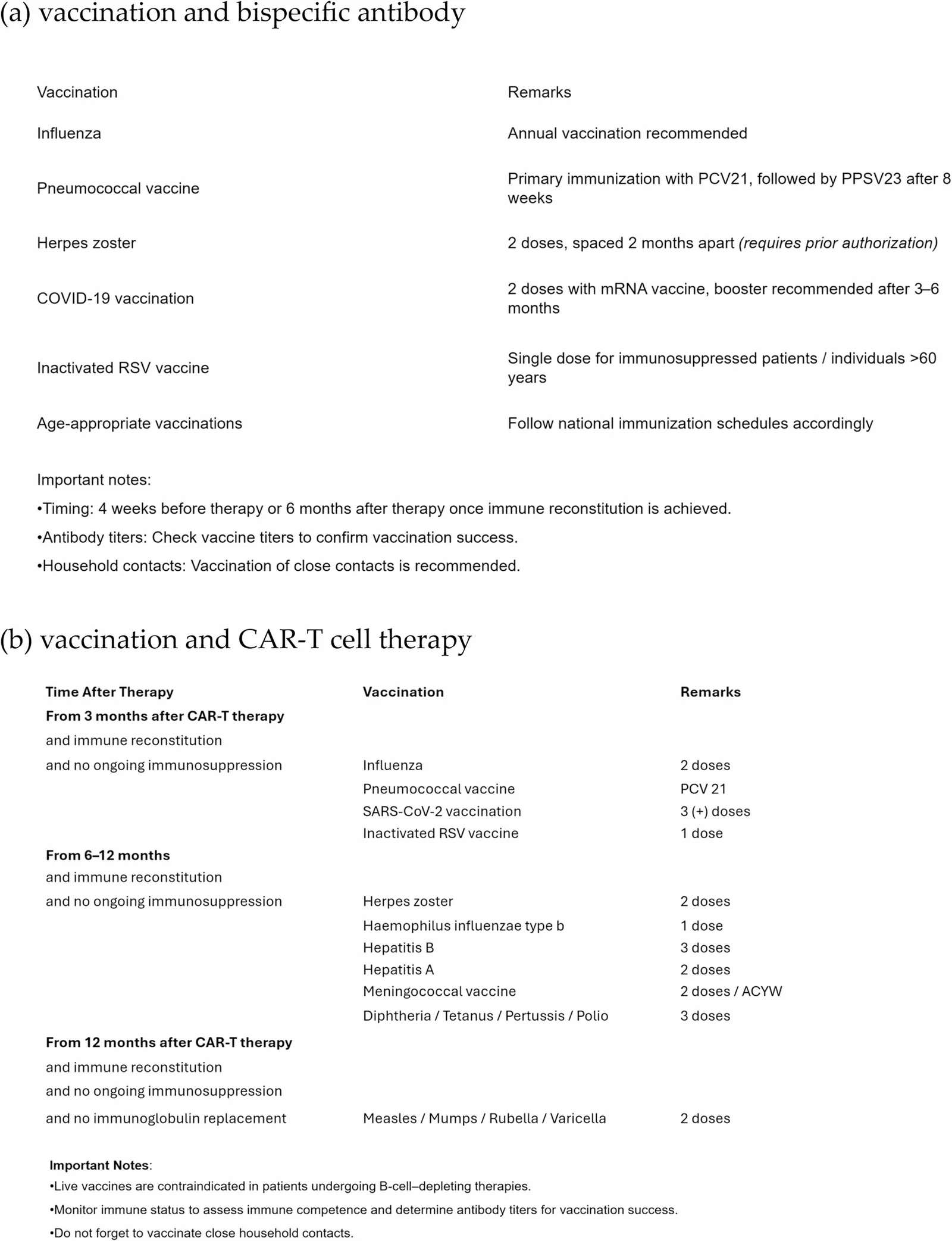
Displays a potential recommendation for vaccination bevor (preferably) and after bispecific antibody treatment (**a**) and CAR-T cell therapy (**b**). Recommended vaccinations following CAR-T cell therapy and treatment with bispecific antibodies. . [84–86] Abbreviations: PCV21 – 21-valent pneumococcal conjugate vaccine; PPSV23 – 23-valent pneumococcal polysaccharide vaccine; ACYW – meningococcal vaccine covering serogroups A, C, Y, and W

Both ide-cel and cilta-cel demonstrate high efficacy, including high-risk populations [81, 84, 94, 97]. Real-world data suggest higher efficacy but increased infectious toxicity with cilta-cel compared with ide-cel [98, 102, 104]. Infections occur predominantly early: within 30 days they are largely cytopenia-related, whereas infections between days 30–100 reflect delayed immune reconstitution [86]. These patterns support selective early antibacterial and antifungal prophylaxis, continued antiviral and Pneumocystis prophylaxis until immune recovery, and risk-adapted use of G-CSF guided by the CAR-HEMATOTOX score [109]. Immunoglobulin replacement is commonly employed. Post–CAR-T cell mRNA vaccination has demonstrated preserved immunogenicity in most patients and should be completed prior to therapy whenever feasible [115]. Suggested vaccination schedules are summarized in Fig. 4 [132–134].

Other myeloma regimens (SVd, IsaKd, BeMafPd) achieve meaningful responses but were studied in less heavily pretreated, lower-risk populations. Infection rates are generally lower; however, regimen-specific toxicities (e.g., gastrointestinal, cardiac, ocular) remain relevant and should be considered in treatment selection and supportive care planning [135–137].

Heterogeneity in follow-up, patient selection, infection definitions, and supportive care limits direct comparability. Nevertheless, these data remain clinically relevant, as they address aspects not captured in pivotal trials, including frailty, vaccine responsiveness, and supportive-care strategies. Frailty—rather than age—emerges as a key determinant of infection risk. Observational data suggest a lower cumulative infectious burden with CAR-T than with continuously administered BsAbs, although these comparisons are limited by differences in exposure duration and follow-up. Variability across SARS-CoV-2 waves further complicates interpretation. Moreover, most studies report infection incidence and severity but provide limited information regarding infection duration, time to resolution, or patient-reported outcomes. As prolonged or recurrent infections may substantially affect quality of life and healthcare utilization, future studies should incorporate these parameters as clinically meaningful endpoints.

The literature search was restricted to PubMed/MEDLINE, which may have limited capture of relevant studies. Nonetheless, the large number of included studies and the breadth of agents provide a broad overview of the available evidence.

Despite methodological heterogeneity, consistent prophylactic strategies—including antiviral and Pneumocystis prophylaxis, early immunoglobulin replacement, and vaccination—can reduce, though not eliminate, infection-related morbidity in patients receiving T-cell–redirecting therapies. Further studies are warranted to evaluate future approaches, including time-limited minimal residual disease (MRD)-driven treatment strategies and immune-supportive interventions.

## Conclusions

Infections remain a major limitation of CAR-T cell therapies and BsAbs, largely driven by persistent B-cell aplasia and hypogammaglobulinemia. Early immunoglobulin replacement in hypogammaglobulinemic patients appears to be an important component of infection mitigation, complemented by routine PJP and antiviral prophylaxis with risk-adapted antibacterial and antifungal strategies. Frailty-informed patient selection may help optimize treatment selection and supportive care intensity. Prospective studies are needed to define optimal treatment duration, particularly fixed-duration or response-adapted BsAb approaches, to reduce cumulative infectious risk while preserving efficacy.

## Supplementary Information

Below is the link to the electronic supplementary material.

Supplementary Material 1 (DOCX 55.0 KB)

Supplementary Material 2 (DOCX 95.1 KB)

Supplementary Material 3 (DOCX 133 KB)

Supplementary Material 4 (DOCX 90.5 KB)

## Data Availability

No datasets were generated or analysed during the current study.
