# Supplementary material for: Infectious toxicities associated with bispecific antibodies and CAR-T Cells in multiple myeloma: a systematic review
Source: Ann Hematol. 2026 Jun 18;105(9):397. doi: 10.1007/s00277-026-07140-8 (PMC13521976; doi:10.1007/s00277-026-07140-8)
Supplement: Supplementary file 1 [file 277_2026_7140_MOESM1_ESM.docx]

| Supplementary Table 2: Risk of bias assessment of included studies using ROBINS-I and RoB 2 tools | | | | | | | | | |  |
| --- | --- | --- | --- | --- | --- | --- | --- | --- | --- | --- |
| Moderate | **Studytype** | **Bias Tool** | **D1** | **D2** | **D3** | **D4** | **D5** | **D6** | **D7** | **Overall** |
| *Sanoyan, Seipel et al. 2023* | Real-World | ROBINS-I | Moderate | Moderate | Moderate | Moderate | Moderate | Moderate | Moderate | Moderate |
| *Contejean, Janssen et al. 2023* | Real- World | ROBINS-I | Serious | Serious | Moderate | Low | Moderate | Serious | Moderate | Serious |
| *Ellithi, Elsallab et al. 2025* | Real- World | ROBINS-I | Serious | Serious | Moderate | Low | Moderate | Serious | Moderate | Serious |
| *Wang, Cao et al. 2022* | Phase II | ROBINS-I | Moderate | Moderate | Moderate | Low | Low | Low | Low | Moderate |
| *Iida, Ito et al. 2024* | Phase I/II | ROBINS-I | Moderate | Moderate | Low | Low | Low | Low | Low | Moderate |
| *Banerjee, Marsal et al 2021* | Real-World | ROBINS-I | Serious | Moderate | Low | Moderate | Low | Low | Low | Serious |
| *Sim, Longhitano et al. 2023* | Real- World | ROBINS-I | Serious | Moderate | Low | Moderate | Moderate | Moderate | Moderate | Serious |
| *Lancman, Parsa et al. 2023* | Real-World | ROBINS-I | Serious | Moderate | Low | Moderate | Moderate | Low | Low | Serious |
| *Li, Zhang et al. 2025* | Real- World | ROBINS-I | Serious | Serious | Moderate | Moderate | Moderate | Serious | Serious | Serious |
| *Hammons, Haider et al. 2024* | Real-World | ROBINS-I | Serious | Serious | Moderate | Low | Moderate | Low | Moderate | Serious |
| *Gaballa, Puglianini et al. 2025* | Real- World | ROBINS-I | Serious | Moderate | Moderate | Moderate | Moderate | Moderate | Moderate | Serious |
| *Ariane, Stéphanie et al. 2025* | Real-World | ROBINS-I | Moderate | Low | Moderate | Moderate | Moderate | Moderate | Moderate | Moderate |
| *Kawasaki, Steele et al. 2024* | Real-World | ROBINS-I | Serious | Moderate | Low | Moderate | Moderate | Moderate | Moderate | Serious |
| *Lesokhin, Richter et al. 2022* | Phase I | ROBINS-I | Serious | Serious | Low | Moderate | Moderate | Moderate | Moderate | Serious |
| *Topp, Duell et al. 2020* | Phase I | ROBINS-I | Serious | Moderate | Low | Moderate | Moderate | Low | Moderate | Serious |
| *Park, Jang et al 2025* | Real-World | ROBINS-I | Moderate | Moderate | Low | Moderate | Moderate | Moderate | Moderate | Moderate |
| *Uttervall, Tätting et al. 2024* | Real-World | ROBINS-I | Serious | Moderate | Moderate | Moderate | Moderate | Moderate | Moderate | Serious |
| *Hammons, Szabo et al. 2024* | Real- World | ROBINS-I | Moderate | Moderate | Low | Moderate | Moderate | Moderate | Low | Moderate |
| *Wang, Lii et al 2021* | Real-World | ROBINS-I | Serious | Low | Moderate | Moderate | Low | Moderate | Moderate | Serious |
| *Frenking, Zhou et al 2024* | Real-World | ROBINS-I | Moderat | Low | Low | Moderate | Moderate | Low | Low | Moderate |
| *Richardson, Schütte et al. 2025* | Real-World | ROBINS-I | Moderate | Moderate | Low | Moderate | Moderate | Low | Low | Moderate |
| *Merz Gagelmann et al* | Real – World | ROBINS-I | Moderate | Moderate | Low | Moderate | Moderate | Low | Low | Moderate |
| *Malard, Bobin et al. 2024* | Real – World | ROBINS-I | Moderate | Moderate | Low | Moderate | Moderate | Low | Low | Moderate |
| *Schinke, Dhakal et al. 2024* | Real – World | ROBINS-I | Serious | Serious | Moderate | Moderate | Low | Moderate | Moderate | Serious |
| *Mohan, Monge et al. 2024* | Real – World | ROBINS-I | Moderate | Moderate | Low | Moderate | Moderate | Moderate | Moderate | Moderate |
| *Perrot, Hulin et al. 2024* | Real – World | ROBINS-I | Serious | Moderate | Low | Moderate | Moderate | Moderate | Low | Serious |
| *Cohen, Magen et al. 2025* | Phase I/II | ROBINS-I | Moderate | Moderate | Moderate | Low | Low | Low | Low | Moderate |
| *Oliver-Caldés, González-Calle et al. 2023* | Phase I/II | ROBINS-I | Serious | Moderate | Serious | Serious | Moderate | Low | Moderate | Serious |
| *Trudel, Cohen et al. 2021* | Phase I/II | ROBINS-I | Moderate | Serious | Moderate | Low | Low | Low | Low | Serious |
| *Munshi, Anderson et al. 2021;^10^* | Phase I/II | ROBINS-I | Moderate | Moderate | Moderate | Moderate | Low | Low | Low | Moderate |
| *Chari, Minnema et al. 2022* | Phase I/II | ROBINS-I | Serious | Moderate | Low | Moderate | Moderate | Moderate | Moderate | Moderate |
| *Cai, Xia et al 2024* | Phase I/II | ROBINS-I | Serious | Moderate | Low | Moderate | Moderate | Low | Moderate | Serious |
| *Ishida, Kuroda et al. 2025* | Phase I/II | ROBINS-I | Serious | Moderate | Low | Moderate | Moderate | Moderate | Moderate | Serious |
| *Nooka, Rodriguez et al. 2024* | Phase I/II | ROBINS-I | Serious | Moderate | Low | Moderate | Moderate | Moderate | Moderate | Serious |
| *Kowalski, Lykon et al. 2025* | Real-World | ROBINS-I | Serious | Serious | Low | Moderate | Moderate | Moderate | Moderate | Serious |
| *Mohan, Szabo et al. 2025* | Real-World | ROBINS-I | Serious | Moderate | Moderate | Moderate | Moderate | Moderate | Moderate | Serious |
| *Josyula, Pont et al. 2022* | Real-World | ROBINS-I | Serious | Moderate | Moderate | Low | Moderate | Moderate | Low | Serious |
| *Thiruvengadam, Sheng et al. 2022* | Real-World | ROBINS-I | Serious | Moderate | Low | Moderate | Moderate | Moderate | Low | Serious |
| *Reyes, Huang et al 2023* | Real- world | ROBINS-I | Serious | Serious | Low | Moderate | Moderate | Moderate | Moderate | Serious |
| *Lesokhin, Nath et al 2024* | Real-world | ROBINS-I | Serious | Serious | Moderate | Moderate | Serious | Low | Moderate | Serious |
| *Howard, Concepcion et al. 2024* | Real-world | ROBINS-I | Serious | Serious | Low | Moderate | Moderate | Moderate | Moderate | Serious |
| *Sidana, Patel et al.2025* | Real- world | ROBINS-I | Moderate | Moderate | Low | Low | Moderate | Moderate | Low | Moderate |
| *Caillot, Sleiman et al 2024* | Real-world | ROBINS-I | Serious | Moderate | Low | Low | Moderate | Moderate | Moderate | Serious |
| *Kikuchi, Watanabe et al* | Real-world | ROBINS-I | Moderate | Moderate | Low | Low | Low | Moderate | Moderate | Moderate |
| *Dima, Rashid et al 2024* | Real-world | ROBINS-I | Serious | Serious | Low | Moderate | Moderate | Moderate | Low | Serious |
| *Ferreri, Hildebrandt et al. 2023;* | Real-world | ROBINS-I | Serious | Moderate | Low | Moderate | Moderate | Moderate | Low | Serious |
| *Tan, Asoori et al. 2025* | Real-world | ROBINS-I | Serious | Moderate | Low | Moderate | Moderate | Moderate | Low | Serious |
| *Riedhammer, Bassermann et al. 2024;* | Real-world | ROBINS-I | Serious | Moderate | Low | Low | Moderate | Moderate | Low | Serious |
| *Zhao, Wang et al. 2022* | Phase I/II | ROBINS-I | Serious | Moderate | Low | Moderate | Low | Moderate | Moderate | Serious |
| *Ravi, Richard et al. 2025* | Phase I/II | ROBINS-I | Serious | Moderate | Low | Moderate | Moderate | Moderate | Moderate | Serious |
| *Mailankody, Matous et al. 2023* | Phase I/II | ROBINS-I | Serious | Moderate | Low | Moderate | Moderate | Moderate | Moderate | Serious |
| *Htut, Dhakal et al. 2023* | Phase I/II | ROBINS-I | Serious | Serious | Low | Moderate | Moderate | Low | Moderate | Serious |
| *Ri, Suzuki et al. 2022* | Phase I/II | ROBINS-I | Serious | Serious | Low | Moderate | Moderate | Low | Moderate | Serious |
| *Tomasson, Iida et al. 2024* | Phase I/II | ROBINS-I | Serious | Serious | Low | Moderate | Low | Moderate | Moderate | Serious |
| *Lin, Raje et al. 2023* | Phase I/II | ROBINS-I | Serious | Serious | Low | Moderate | Moderate | Moderate | Moderate | Serious |
| *Bumma, Richter et al. 2024* | Phase I/II | ROBINS-I | Serious | Serious | Low | Moderate | Moderate | Moderate | Moderate | Serious |
| *Iida, Sunami et al 2025* | Phase I/II | ROBINS-I | Serious | Serious | Low | Moderate | Moderate | Moderate | Moderate | Serious |
| *Touzeau, Krishnan et al. 2024;* | Phase I/II | ROBINS-I | Serious | Moderate | Low | Low | Low | Low | Moderate | Serious |
| *Frerichs, Verkleij et al. 2024* | Phase I/II | ROBINS-I | Serious | Moderate | Low | Moderate | Moderate | Low | Moderate | Serious |
| *Usmani, Garfall et al. 2021* | PhaseI/II | ROBINS-I | Serious | Serious | Low | Moderate | Moderate | Moderate | Low | Serious |
| *Choi, Buyn et al 2024* | Real-World | ROBINS-I | Moderate | Moderate | Low | Moderate | Moderate | Moderate | Low | Moderate |
| *Jourdes, Cellerin et al. 2024* | Real-World | ROBINS-I | Serious | Moderate | Low | Moderate | Moderate | Moderate | Low | Serious |
| *Little, Tandon et al. 2023* | Real-World | ROBINS-I | Moderate | Moderate | Low | Low | Moderate | Moderate | Low | Moderate |
| *Rejeski, Hansen et al 2023* | Real-World | ROBINS-I | Serious | Moderate | Low | Low | Moderate | Moderate | Moderate | Serious |
| *Wesson, Dima et al. 2024* | Real-World | ROBINS-I | Serious | Moderate | Low | Moderate | Moderate | Moderate | Moderate | Serious |
| *Merz, Albici et al. 2025* | Real-World | ROBINS-I | Serious | Moderate | Low | Moderate | Moderate | Moderate | Moderate | Serious |
| *Nath, Shekarkhand et al. 2024;* | Real-World | ROBINS-I | Serious | Serious | Moderate | Moderate | Moderate | Moderate | Moderate | Serious |
| *Trando, Ghamsari et al. 2024;* | Real-World | ROBINS-I | Serious | Serious | Serious | Moderate | Moderate | Moderate | Moderate | Serious |
| *Sidana, Ahmed et al. 2025;* | Real-World | ROBINS-I | Moderate | Low | Low | Moderate | Moderate | Low | Low | Moderate |
| *Kalariya, Hildebrandt et al. 2024;* | Real-World | ROBINS-I | Moderate | Moderate | Low | Moderate | Moderate | Low | Low | Moderate |
| *Dima, Davis et al. 2024* | Real-World | ROBINS-I | Moderate | Moderate | Low | Moderate | Moderate | Low | Low | Moderate |
| *Xia, Sun et al 2025.* | Phase I/II | ROBINS-I | Serious | Serious | Low | Moderate | Low | Moderate | Low | Serious |
| *Xu, Wang et al* | Phase I/II | ROBINS-I | Serious | Moderate | Low | Moderate | Moderate | Moderate | Low | Serious |
| *Mi, Zhao et al. 2023* | Phase I/II | ROBINS-I | Serious | Serious | Low | Moderate | Moderate | Moderate | Low | Serious |
| *Berdeja, Madduri et al. 2021;* | Phase I/II | ROBINS-I | Serious | Moderate | Low | Moderate | Low | Moderate | Low | Serious |
| *Bahlis, Costello et al. 2023* | Phase I/II | ROBINS-I | Serious | Moderate | Low | Moderate | Moderate | Low | Low | Serious |
| *Lesokhin, Tomasson et al. 2023* | Phase I/II | ROBINS-I | Serious | Moderate | Low | Moderate | Moderate | Low | Low | Serious |
| *Minakata, Ishida et al. 2023 – Phase 2* | Phase I/II | ROBINS-I | Serious | Serious | Low | Moderate | Moderate | Low | Moderate | Serious |
| *Chari, Touzeau et al. 2025;* | Phase I/II | ROBINS-I | Serious | Moderate | Low | Moderate | Low | Low | Moderate | Serious |
| *Moreau et al N Engl J med 2022;* | Phase I/II | ROBINS-I | Serious | Moderate | Low | Moderate | Low | Moderate | Moderate | Serious |
| *Hill, Martens et al. 2024* | Real-World | ROBINS-I | Serious | Moderate | Moderate | Low | Moderate | Moderate | Low | Moderate |
| *Portuguese, Liang et al. 2025;* | Real-World | ROBINS-I | Serious | Moderate | Moderate | Moderate | Moderate | Moderate | Moderate | Serious |
| *Hansen, Peres et al. 2025* | Real-World | ROBINS-I | Moderate | Moderate | Moderate | Moderate | Low | Moderate | Low | Moderate |
| *Logue, Peres et al. 2022* | Real-World | ROBINS-I | Moderate | Moderate | Low | Moderate | Moderate | Moderate | Moderate | Moderate |
| *Hansen, Sidana et al. 2023* | Real-World | ROBINS-I | Moderate | Moderate | Moderate | Serious | Moderate | Moderate | Low | Serious |
| *Frenking, Riedhammer et al. 2025* | Real-World | ROBINS-I | Serious | Moderate | Moderate | Moderate | Moderate | Moderate | Low | Serious |
| *Akhtar, Oloyede et al. 2025;* | Real-World | ROBINS-I | Moderate | Moderate | Moderate | Low | Moderate | Low | Moderate | Moderate |
| *Hashmi, Hansen et al. 2024* | Real-World | ROBINS-I | Serious | Moderate | Moderate | Moderate | Moderate | Moderate | Moderate | Serious |
| *Dhakal, Akhtar et al 2025* | Real- World | ROBINS-I | Serious | Serious | Low | Moderate | Moderate | Moderate | Moderate | Serious |
| *Kumar, Mateos et al 2026* | Phase-II | ROBINS-I | Serious | Moderate | Low | Moderate | Moderate | Low | Moderate | Serious |
| *Razzo, Midha et al 2026* | Real-Wold | ROBINS-I | Serious | Moderate | Low | Moderate | Moderate | Moderate | Moderate | Serious |
| *Schinke, Rodriguez-Otero et al 2025* | Phase I/II | ROBINS-I | Serious | Moderate | Low | Moderate | Moderate | Low | Moderate | Serious |
| *Cani, Scott et al 2025* | Real-World | ROBINS-I | Serious | Moderate | Low | Moderate | Moderate | Moderate | Moderate | Serious |
| *Al Hadidi, Szabo et al 2025* | Real-World | ROBINS-I | Serious | Moderate | Low | Moderate | Moderate | Moderate | Moderate | Serious |
| *Lee, Zonder et al 2025* | Phase I/II | ROBINS-I | Serious | Moderate | Low | Moderate | Moderate | Moderate | Low | Serious |
| *Bros, Harel et al 2025* | Real-World | ROBINS-I | Serious | Moderate | Moderate | Moderate | Serious | Moderate | Moderate | Serious |
| *Soltantabar, Hibma et al 2026* | Phase I/II | ROBINS-I | Serious | Moderate | Low | Moderate | Moderate | Low | Moderate | Serious |
| *Snyder, Atrash et al 2025* | Real-World | ROBINS-I | Serious | Serious | Low | Moderate | Moderate | Moderate | Moderate | Serious |
| *Stork, Radocha et al 2025* | Real-World | ROBINS-I | Serious | Moderate | Moderate | Moderate | Moderate | Low | Moderate | Serious |
| *Dima, Loque et al 2026* | Real-World | ROBINS-I | Serious | Moderate | Low | Moderate | Moderate | Low | Moderate | Serious |
| *Yi, Lee et al 2025* | Real-World | ROBINS-I | Serious | Moderate | Low | Moderate | Moderate | Low | Moderate | Serious |
| *Wang, Li et al 2026* | Real-World | ROBINS-I | Moderate | Moderate | Low | Moderate | Moderate | Moderate | Moderate | Moderate |
| *Jelinek, Zihala et al 2026* | Real-World | ROBINS-I | Moderate | Moderate | Low | Moderate | Moderate | Low | Moderate | Moderate |
| *Hansen, Dima et al 2026* | Real-World | ROBINS-I | Moderate | Moderate | Low | Moderate | Moderate | Low | Low | Moderate |
| *Piron, Rodrigo et al 2025* | Real World | ROBINS-I | Serious | Moderate | Low | Moderate | Moderate | Moderate | Moderate | Serious |
| *Portuguese, Davis et al 2026* | Real-World | ROBINS-I | Serious | Moderate | Low | Moderate | Moderate | Low | Moderate | Serious |
| *Smits, Groen et al 2026* | Real-World | ROBINS-I | Serious | Moderate | Low | Moderate | Moderate | Low | Moderate | Serious |
| *Bilbao, Anson et al 2025* | Real-World | ROBINS-I | Serious | Moderate | Low | Moderate | Moderate | Low | Moderate | Serious |
| *Ssentongo, Guare et al 2026* | Real-World | ROBINS-I | Serious | Moderate | Low | Moderate | Moderate | Low | Moderate | Serious |
| *Cheema, Shrestha et al 2026* | Real-World | ROBINS-I | Serious | Moderate | Low | Moderate | Low | Low | Moderate | Serious |
| *Tan, Usmani et al 2026* | Real-World | ROBINS-I | Serious | Low | Low | Moderate | Moderate | Low | Moderate | Serious |
| *Little, Pena et al 2025* | Real-World | ROBINS-I | Serious | Moderate | Low | Moderate | Moderate | Low | Moderate | Serious |
| *Yan, Du et al 2026* | Phase II | ROBINS-I | Serious | Moderate | Low | Low | Low | Low | Moderate | Serious |
| *Cohen, Lebel et al 2026* | Real-World | ROBINS-I | Serious | Moderate | Low | Low | Low | Low | Moderate | Serious |
| *Bar, Martin et al 2026* | Phase I | ROBINS-I | Serious | Moderate | Low | Moderate | Low | Low | Moderate | Serious |
| *Habib, Ahmed et al 2026* | Real-World | ROBINS-I | Moderate | Low | Low | Low | Low | Low | Moderate | Moderate |
| *Janakiram, Tan et al 2026* | Real- World | ROBINS-I | Serious | Low | Low | Low | Low | Low | Moderate | Serious |
| *Lee, Plattel et al 2026* | Phase I | ROBINS-I | Serious | Moderate | Low | Low | Moderate | Low | Moderate | Serious |
| *Liu, Zhang et al 2026* | Real-World | ROBINS-I | Serious | Moderate | Low | Low | Moderate | Low | Moderate | Serious |
| *Mersi, Burow et al 2026* | Real-World | ROBINS-I | Moderate | Moderate | Low | Low | Moderate | Moderate | Low | Moderate |
| Moderate | **Studytype** | **Bias Tool** | **Bias from randomization** | **Bias due to deviation** | **Bias due to missing outcome data** | **Bias in measurement** | **Bias in selection** |  |  | **Overall** |
| *San-Miguel, Dhakal et al.2023* | Phase III | RoB2 | Low | Moderate | Low | Low | Moderate |  |  | Moderate |
| *Ailawadhi, Arnulf et al 2024* | Phase III | RoB2 | Low | Moderate | Moderate | Low | Low |  |  | Moderate |
| *Einsele, San-Miguel et al 2026* | Phase III | RoB2 | Low | Moderate | Low | Low | Moderate |  |  | Moderate |
| *Costa, Bahlis et al 2025* | Phase III | RoB2 | Low | Moderate | Low | Low | Low |  |  | Moderate |

**Supplementary Table 2:** Risk of bias was assessed using ROBINS-I for non-randomized studies (real-world and phase I/II studies) and RoB 2 for randomized phase III trials. ROBINS-I evaluates seven domains (D1–D7), including confounding, participant selection, intervention classification, deviations from intended interventions, missing data, outcome measurement, and selective reporting, whereas RoB 2 assesses bias arising from randomization, deviations from intended interventions, missing outcome data, outcome measurement, and selective reporting. Risk of bias was rated as low, moderate, or serious, and the overall judgment reflects the highest risk identified across domains.
