# Supplementary material for: Infectious toxicities associated with bispecific antibodies and CAR-T Cells in multiple myeloma: a systematic review
Source: Ann Hematol. 2026 Jun 18;105(9):397. doi: 10.1007/s00277-026-07140-8 (PMC13521976; doi:10.1007/s00277-026-07140-8)
Supplement: Supplementary file 2 [file 277_2026_7140_MOESM2_ESM.docx]

**Supplementary Table 1**. Methodological quality assessment using the Critical Appraisal Skills Programme (CASP)

| *Study* | Study type | CASP tool | Key CASP items |
| --- | --- | --- | --- |
| *Sanoyan, Seipel et al. 2023* | Real-World | CASP Cohort Study Checklist | - Retrospective real-world cohort → selection and documentation bias - No control/comparator group → descriptive only, no causal inference - No multivariable adjustment → residual confounding likely - Short follow-up (median 5.7 months) → durability and late infections underestimated - Small sample size → limited precision and generalizability - Infection outcomes retrospectively captured → ascertainment bias |
| *Contejean, Janssen et al. 2023* | Real-World | CASP Cohort Study Checklist | - Pharmacovigilance database → no denominator, no incidence estimation - Strong reporting and notoriety bias - No patient-level data → confounding uncontrolled - No adjustment for disease severity or co-medications - Signal-generating only; no causal inference |
| *Ellithi, Elsallab et al. 2025* | Real-World | CASP Cohort Study Checklist | - Pharmacovigilance design → no denominator, no absolute risk estimation - Reporting bias and duplicate reports possible - No patient-level data → confounding by disease severity unaddressed - Disproportionality analyses are hypothesis-generating only - Cannot compare agents or quantify excess risk |
| *Wang, Cao et al. 2022* | Phase II | CASP Cohort Study Checklist | - Single-arm phase II study → no comparator - Non-randomized design → confounding likely - Limited adjustment despite multivariable analyses - Precision limited by sample size |
| *Iida, Ito et al. 2024* | Phase I/II | CASP Cohort Study Checklist | - Single-arm, descriptive subgroup analysis - Very small sample size → low statistical precision - No control for confounding - Limited long-term inference |
| *Banerjee, Marsal et al 2021* | Real world | CASP Cohort Study Checklist | - Retrospective design → selection bias - No adjustment for confounding - Small subgroup sizes → imprecise estimates |
| *Sim, Longhitano et al. 2023* | Real- World | CASP Cohort Study Checklist | - retrospective single-centre design - llimited control of confounding - small sample size → reduced generalizability. |
| *Lancman, Parsa et al. 2023* | Real- World | CASP Cohort Study Checklist | - Retrospective single-center cohort → selection bias - Small sample size (n=37) → low statistical power - Non-randomized IVIG use → confounding by indication - Heterogeneous bispecific antibodies and dosing - Limited external validity |
| *Li, Zhang et al. 2025* | Real- World | CASP Cohort Study Checklist | - Very small sample (n=29 total) → unstable estimates - Single-center cohort → limited generalizability - Non-randomized groups → confounding likely - Selection restricted to infected patients → selection bias - High-dimensional protein screening → multiplicity / false-positive risk - Single post-infection sampling → timing variability |
| *Hammons, Haider et al. 2024* | Real-World | CASP Cohort Study Checklist | - Retrospective, non-randomized design - Small sample size (n=33) and unequal group sizes - Highly selected post-allo-HCT population - Heterogeneous CAR-T and bispecific agents - Limited adjustment for confounding - Descriptive comparisons only |
| *Gaballa, Puglianini et al. 2025* | Real-World | CASP Cohort Study Checklist | - Retrospective design with no comparator - Very small sample (n=10) → imprecise estimates - Selection and referral bias (tertiary centers) - Heterogeneous CAR-T products - CNS disease heterogeneity and incomplete CSF data - Variable bridging and CNS-directed therapies → confounding - Possible misclassification and timing bias |
| *Ariane, Stéphanie et al. 2025* | Real- world | CASP Cohort Study Checklist | - Retrospective single-center cohort → selection bias - Non-randomized HN-Ig exposure → confounding by indication - Inconsistent infection ascertainment → documentation bias - No standardized prophylaxis or monitoring framework - Time-trend bias due to evolving practice - Small sample → limited causal inference |
| *Kawasaki, Steele et al. 2024* | Real- world | CASP Cohort Study Checklist | - Retrospective single-center design - Very small comparator group (n=4) → unstable rates - Non-random allocation with time-trend bias - Selection bias from excluding incomplete step-up dosing - Residual confounding (disease burden, comorbidities, medications) - Later toxicities and infections may be under-captured |
| *Lesokhin, Richter et al. 2022* | Real- world | CASP Cohort Study Checklist | - Selected responder-completer cohort → landmark and survivorship bias - Phase I, single-arm design → no comparator - Very small sample (n=16) with immature follow-up - Informative censoring possible - Dose heterogeneity within cohort - Abstract-only reporting → limited methodological detail |
| *Topp, Duell et al. 2020* | Phase I | CASP Cohort Study Checklist | - Phase I single-arm dose-escalation → no comparator - Very small per-dose cohorts → imprecision - Restrictive eligibility → limited generalizability - Heterogeneous exposure duration → survivor bias risk - Supportive care variability across centers |
| *Park, Jang et al 2025* | Real-World | CASP Cohort Study Checklist | - Retrospective single-center design → selection bias - Exposure heterogeneity (different BsAbs, trial patients included) - Center-specific prophylaxis and pathogen epidemiology - Subgroup analyses confounded by disease severity - Outcomes focus on management-relevant events → mild infections under-captured - Limited precision for rare infections |
| *Uttervall, Tätting et al. 2024* | Real-World | CASP Cohort Study Checklist | - Retrospective, non-randomized cohort - Confounding by indication likely - No concurrent control group - Heterogeneous treatments and dosing - Infection surveillance not standardized - Restricted generalizability |
| *Hammons, Szabo et al. 2024* | Real-World | CASP Cohort Study Checklist | - Selection bias due to clinical-trial population - Mixed targets and combination therapies → exposure confounding - Variable prophylaxis practices → outcome distortion - Small subgroup sizes → unstable estimates - Retrospective infection ascertainment - Differing exposure durations |
| *Wang, Lii et al 2021* | Real-World | CASP Cohort Study Checklist | - Responder-only cohort → survivorship bias - Informative censoring at progression or next therapy - Residual confounding from supportive care and immune status - Selective and missing laboratory data |
| *Frenking, Zhou et al 2024* | Real-World | CASP Cohort Study Checklist | - Retrospective study design - Missing data for selected endpoints - Center-specific differences in management practices - No prospective validation |
| *Richardson, Schütte et al. 2025* | Real-World | CASP Cohort Study Checklist | - Retrospective cohort design - Limited control of confounding - Censoring at relapse or next therapy may bias infection incidence - Follow-up heterogeneity |
| *Merz, Gagelmann et al* | Real-World | CASP Cohort Study Checklist | - Non-random treatment allocation → confounding likely - Registry-based follow-up incompleteness possible - Residual confounding despite propensity methods - Limited detail on supportive care practices |
| *Malard, Bobin et al. 2024* | Real-World | CASP Cohort Study Checklist | - Retrospective compassionate-use cohort - Single-arm design → no causal inference - High-risk, trial-ineligible population → confounding by disease severity - Residual confounding from routine-care variability - Heterogeneous supportive care |
| *Schinke, Dhakal et al. 2024* | Real-World | CASP Cohort Study Checklist | - Qualitative design → not comparable to cohort studies - No effect estimates or risk quantification - Findings context-specific; not generalizable |
| *Mohan, Monge et al. 2024* | Real-World | CASP Cohort Study Checklist | - Retrospective design → selection bias - Non-randomized treatment pathways → residual confounding - Heterogeneous prophylaxis and monitoring practices - Short follow-up → durability uncertain - Limited generalizability |
| *Perrot, Hulin et al. 2024* | Real-World | CASP Cohort Study Checklist | - Retrospective cohort design - Confounding only partially addressed - Short follow-up duration - Limited external validity |
| *Cohen, Magen et al. 2025* | Phase I/II | CASP Cohort Study Checklist | - Single-arm early-phase design → no comparator - Limited ability to address confounding - Small sample size → imprecision |
| *Oliver-Caldés, González-Calle et al. 2023* | Phase I/II | CASP Cohort Study Checklist | - Single-arm pilot study → no comparator - Small sample size → limited precision - Interim analysis with immature follow-up |
| *Trudel, Cohen et al. 2021* | Phase I/II | CASP Cohort Study Checklist | - Single-arm design → no causal inference - Confounders described but not controlled - Trial-eligible population → limited generalizability |
| *Ailawadhi, Arnulf et al. 2024* | Phase III | CASP Randomised Controlled Trial Checklist | - Open-label design: potential influence on co-interventions, AE reporting, and clinical decision-making. - High crossover (SR → ide-cel): confounds overall survival; ITT OS becomes hard to interpret. - Attrition before infusion: a meaningful fraction in the ide-cel arm never received ide-cel, linked to early deaths/progression → dilutes/complicates ITT interpretation. - Bridging therapy constraints (≤1 cycle) may have been insufficient for aggressive disease, increasing early events before infusion. - Early mortality imbalance (more early deaths in ide-cel arm largely among non-infused patients) affects OS curve shape and interpretation. |
| *Munshi, Anderson et al. 2021;^10^* | Phase I/II | CASP Cohort Study Checklist | - Single-arm phase II study → no comparator - Confounding not addressable - Limited external validity |
| *Chari, Minnema et al. 2022* | Phase I/II | CASP Cohort Study Checklist | - Single-arm phase I study → no comparator - Selection limits generalizability - Exposure and follow-up heterogeneity - Attrition and informative censoring possible |
| *Cai, Xia et al 2024* | Phase I/II | CASP Cohort Study Checklist | - Single-arm phase II cohort → no relative efficacy estimation - Small sample size → wide uncertainty - Substantial censoring of durability endpoints - Context-dependent infection risk. |
| *Ishida, Kuroda et al. 2025* | Phase I/II | CASP Cohort Study Checklist | - Single-arm open-label design → no comparator - Small sample size → rare AEs may be missed - Selection of fitter patients → limited generalizability - Immature survival data. |
| *Nooka, Rodriguez et al. 2024* | Phase I/II | CASP Cohort Study Checklist | - Single-arm descriptive analysis - Substantial confounding from disease biology and pretreatment - Heterogeneous prophylaxis and COVID-era effects - Attrition bias over time - Exploratory statistics only |
| *Kowalski, Lykon et al. 2025* | Phase I/II | CASP Cohort Study Checklist | - Single-center, non-randomized cohort - No comparator group - Confounding by center-specific supportive care - Limited external validity |
| *Mohan, Szabo et al. 2025* | Real-World | CASP Cohort Study Checklist | - Non-randomized IVIG exposure → confounding by indication - Residual immortal-time bias possible - Heterogeneous treatments and evolving practice over time - Retrospective, multi-center outcome ascertainment |
| *Josyula, Pont et al. 2022* | Real-World | CASP Cohort Study Checklist | - Small retrospective single-center cohort - Highly selected trial population - Major confounding from CRS severity and immunosuppression - Limited precision |
| *Thiruvengadam, Sheng et al. 2022* | Real-World | CASP Cohort Study Checklist | - Retrospective single-center design - Selection bias and residual confounding - Non-standardized prophylaxis - Short follow-up → late infections underestimated |
| *Reyes, Huang et al 2023* | Real-World | CASP Cohort Study Checklist | - Retrospective single-center cohort - Selection bias favoring fitter older patients - Residual confounding (frailty not systematically assessed) - Small subgroup sizes → underpowered comparisons |
| *Lesokhin, Nath et al 2024* | Real-World | CASP Cohort Study Checklist | - Retrospective single-center cohort - Non-randomized exposure → confounding likely - Heterogeneous treatments - Descriptive, non-causal analysis, Hypothesis-generating |
| *Howard, Concepcion et al. 2024* | Real-World | CASP Cohort Study Checklist | - Responder-only cohort → strong survivorship bias - Small sample size → low power - Residual confounding likely - Limited generalizability |
| *Sidana, Patel et al.2025* | Real- World | CASP Cohort Study Checklist | - Retrospective real-world cohort → selection bias - Academic SOC centers → referral bias - Non-random treatment pathways → residual confounding - Investigator-assessed efficacy → potential assessment bias - Moderate follow-up → late toxicities underestimated |
| *Caillot, Sleiman et al 2024* | Real- World | CASP Cohort Study Checklist | - Retrospective single-center cohort - Very small sample size → low precision - Selection and survivorship bias - Major residual confounding - Limited follow-up |
| *Kikuchi, Watanabe et al.* | Real- World | CASP Cohort Study Checklist | - Retrospective single-center design → bias risk - Possible selection and detection bias - Confounding largely unaddressed - Very small number of events → low precision - Safety signal only; incidence uncertain - Limited generalizability |
| *Dima, Rashid et al. 2024;* | Real- World | CASP Cohort Study Checklist | - Retrospective cohort → selection and survivorship bias - Limited control for confounding - Real-world outcome assessment less rigorous than trials - Moderate follow-up → durability uncertain - Limited precision for subgroup analyses |
| *Ferreri, Hildebrandt et al. 2023;* | Real- World | CASP Cohort Study Checklist | - Retrospective cohort → selection and attrition bias - Baseline imbalance between cohorts → residual confounding - Small prior-BCMA subgroups → imprecise estimates - Short follow-up → OS uncertain |
| *Tan, Asoori et al. 2025* | Real- World | CASP Cohort Study Checklist | - Retrospective design → high residual confounding risk - Selection bias toward fitter patients - Short follow-up (~5 months) - Incomplete real-world outcome ascertainment - Heterogeneous management across centers - Underpowered subgroup analyses |
| *Riedhammer, Bassermann et al. 2024;* | Real- World | CASP Cohort Study Checklist | - Retrospective real-world cohort → selection and documentation bias - Inclusion of patients receiving ≥1 full dose only → survivorship bias - No randomized comparator → descriptive, non-causal inference - Short follow-up with heavy censoring → durability and late toxicity uncertain - Residual confounding (disease severity, prior BCMA therapy, center practices) - Heterogeneous outcome ascertainment (CR confirmation, infection surveillance) |
| *Zhao, Wang et al. 2022* | Phase I/II | CASP Cohort Study Checklist | - Single-arm phase 1 → cannot estimate relative benefit/risk or causal effect - Selection + center effects (specialist sites; trial-eligible patients) → limited generalizability - Open-label + multi-site regimen heterogeneity - subgroup/biomarker findings are hypothesis-generating - Late safety signals (viral reactivation, second primaries) based on few events → imprecise |
| *Ravi, Richard et al. 2025* | Phase I/II | CASP Cohort Study Checklist | - Phase 1, single-arm, open-label → no causal/comparative efficacy conclusions - Selection/attrition bias: analyzed infused, conforming-product patients; pre-infusion dropouts + manufacturing failures excluded - Indirect historical comparisons (e.g., vs orva-cel) → strong confounding - Small dose cohorts + rare-event uncertainty → imprecise safety signals (e.g., MDS/SPM) - Follow-up maturity limited for long-term durability and late toxicities |
| *Mailankody, Matous et al. 2023* | Phase I/II | CASP Cohort Study Checklist | - Phase 1, single-arm, open-label (interim) → no causal or comparative conclusions - Small sample size and small subgroups → limited precision; rare adverse events uncertain - Selection and attrition bias (trial population; interim, as-treated analyses) - Heterogeneity in lymphodepletion and supportive care (ALLO-647 regimen, prophylaxis, monitoring) → outcomes potentially influenced - Limited follow-up → long-term safety, late infections, and delayed toxicities not fully assessed |
| *Htut, Dhakal et al. 2023* | Phase I/II | CASP Cohort Study Checklist | - Retrospective subgroup analysis within a single-arm trial → no causal inference; comparison to non-alloSCT is non-randomized. - Very small sample (n=7) → imprecise estimates; rare/late toxicities and GVHD reactivation risk remain uncertain. - Strong selection bias (only clinically stable post-alloSCT, no active GVHD, off immunosuppression; alloSCT ≤6 months excluded) → limited generalizability. - Confounding likely (different baseline risk and treatment history; more heavily pretreated) with no robust adjustment feasible. - Missing key transplant biology data (e.g., donor chimerism of collected T cells) limits interpretation of GVHD risk and mechanism. |
| *Ri, Suzuki et al. 2022* | Phase I/II | CASP Cohort Study Checklist | - Single-arm, open-label, interim analysis → no causal or comparative conclusions. - Very small sample (n=9 infused; mITT n=8) → wide uncertainty; rare AEs and subgroup effects cannot be assessed. - Attrition/selection before infusion (screened/apheresed patients not infused) → potential selection/attrition bias (“treated” population may be healthier). - Bridging therapy and supportive care (incl. mandatory hospitalization + infection prophylaxis) may influence safety/outcomes and limits generalizability. - Short follow-up (~8.5 months) → durability (DOR/PFS/OS) and late toxicities (e.g., infections, neurocognitive events, SPMs) incompletely characterized. - mITT excludes a below-target dose patient → analysis set choice may overestimate efficacy. |
| *Tomasson, Iida et al. 2024* | Phase I/II | CASP Cohort Study Checklist | - Single-arm, open-label→ no causal or comparative conclusions. - small sample (n=9 infused; mITT n=8) → imprecise estimates; rare AEs cannot be reliably detected. - Selection/attrition before infusion (apheresed/screened patients not infused; mITT excludes below-target dose) → potential selection bias and inflated efficacy. - Bridging therapy + intensive supportive care (mandatory hospitalization, prophylaxis/monitoring) may influence outcomes and limits generalizability. - Short follow-up (~8.5 months) → durability (DOR/PFS/OS) and late toxicities (late infections, neurocognitive events, SPMs) not fully characterized. |
| *Lin, Raje et al. 2023* | Phase I/II | CASP Cohort Study Checklist | - Single-arm, open-label Phase 1 → no comparative or causal inference. - Post hoc analyses (incl. biomarker/“correlates”) → hypothesis-generating; multiple testing without correction increases false positives. - Dose-escalation + dose-expansion heterogeneity (different baseline risk, eligibility changes, and dose groups) → confounding by indication and unstable subgroup comparisons. - Small overall sample (n=62 infused) and very small dose subgroups (e.g., 50×10⁶ and 800×10⁶) → wide uncertainty; rare/late AEs imprecise - Pre-infusion attrition (67 apheresis → 62 infused) + bridging therapy in many patients → potential selection/attrition bias. - Supportive care and management (tocilizumab/steroids, infection prophylaxis) not standardized across sites/time → outcomes may be influenced. - Follow-up is moderate (median ~18 months): late toxicities (late infections, cytopenias, secondary malignancies) and long-term durability still not fully captured. |
| *Bumma, Richter et al. 2024* | Phase I/II | CASP Cohort Study Checklist | - Single-arm, open-label Phase 1 → no comparative or causal inference. - Post hoc analyses (efficacy, biomarkers) with multiple testing unadjusted → hypothesis-generating only. - Small sample (n=62 infused) and very small dose subgroups → low precision; rare/late AEs uncertain. - Selection/attrition before infusion and bridging therapy → selection and confounding by indication. - Dose escalation/expansion heterogeneity (baseline risk, doses) → unstable subgroup comparisons. - Supportive care not standardized (tocilizumab/steroids, prophylaxis) → outcome influence. - Follow-up moderate → late toxicities and long-term durability incompletely captured. |
| *Iida, Sunami et al 2025* | Phase I/II | CASP Cohort Study Checklist | - Phase 1, single-arm, open-label → no comparative or causal inference - Very small sample (n=15) and tiny dose cohorts → low precision - Dose-escalation heterogeneity (QW vs Q2W, step-up schedules) - Selection bias (fit trial population; ECOG 0–1) - Confounding by prior therapies/supportive care not controlled - Short follow-up → durability, late AEs, infections uncertain |
| *Touzeau, Krishnan et al. 2024;* | Phase I/II | CASP Cohort Study Checklist | - Single-arm, non-comparative design → no causal inference - Small sample and tiny subgroups (ADC vs CAR-T) → low precision - Selection bias (fit patients, ECOG 0–1) - Heterogeneity of prior BCMA exposure (type, timing, refractoriness) - Residual confounding (disease biology, immune status, sequencing) - Infection risk context-dependent (COVID era, evolving prophylaxis) |
| *Frerichs, Verkleij et al. 2024* | Phase I/II | CASP Cohort Study Checklist | - Non-randomized, post-hoc analyses - Selection bias (trial population, ECOG 0–1) - Small vaccination cohorts → low precision - IVIG effect observational (physician discretion) - Residual confounding (prior therapies, immune status) - Limited long-term immune recovery data |
| *Usmani, Garfall et al. 2021* | Phase I/II | CASP Cohort Study Checklist | - Single-arm, open-label phase 1 → no comparator; no causal efficacy conclusions - Selection bias → highly selected, heavily pretreated trial population - Performance & detection bias → open-label design; subjective AE reporting possible - Confounding not controlled → supportive care, infection prophylaxis, dose cohorts vary - Small RP2D cohort (n=40) → limited precision; rare toxicities may be missed - Short follow-up → durability and long-term safety uncertain - Dose heterogeneity (IV/SC, escalation cohorts) → outcome heterogeneity - Generalizability limited → trial setting, ECOG 0–1 only |
| *Choi, Buyn et al 2024* | Real-world | CASP Cohort Study Checklist | - Retrospective, non-randomized design - Residual confounding despite propensity score matching - Heterogeneous BiTE agents and combinations - Short and unequal follow-up for OS - Subgroup analyses underpowered (BCMA vs non-BCMA) |
| *Jourdes, Cellerin et al. 2024* | Real-world | CASP Cohort Study Checklist | - Retrospective, non-randomized design - Residual confounding despite propensity score matching - Heterogeneous BiTE agents and treatment combination - Short follow-up limits OS interpretation - Subgroup analyses (BCMA vs non-BCMA) underpowered |
| *Little, Tandon et al. 2023* | Real-world | CASP Cohort Study Checklist | - Retrospective observational design - No control or comparator cohort - Potential under-ascertainment of mild infections - Heterogeneous CAR-T products and supportive care - IVIG use not standardized (provider dependent) - Limited power for multivariable risk modeling |
| *Rejeski, Hansen et al 2023* | Real-world | CASP Cohort Study Checklist | - Retrospective observational design (risk of missing/misclassified events) - Prognostic score evaluated without prospective pre-specification/impact design - Heterogeneity across 6 centres (infection prophylaxis, G-CSF use, admission practices) - Limited follow-up duration (toxicity/infection patterns beyond 3–6 months may be underestimated) - Residual confounding likely (disease burden, prior therapies, frailty, centre effects) - Response assessment not centrally reviewed (potential measurement bias) - Small numbers for some endpoints (e.g., NRM, severe ICANS; risk of model instability/overfitting) - Score partly built from baseline cytopenias → may reflect overall illness severity (“prognostic enrichment”) |
| *Wesson, Dima et al. 2024* | Real-world | CASP Cohort Study Checklist | - Retrospective design → risk of missing/misclassified toxicity and infection events - Short observation window (Day 90) → cannot assess late neurotoxicity, late infections, or late NRM - Heterogeneous center practices (admission, prophylaxis, grading, thresholds for “relocation” - Limited confounder adjustment (baseline risk, disease burden, frailty, prior infections) - Low number of NRM events → imprecise estimates and limited subgroup reliability - Infection definitions/diagnostic intensity may differ by center → measurement bias - Relocation and distance are institution-dependent → limits comparability and external validity |
| *Merz, Albici et al. 2025* | Real-world | CASP Cohort Study Checklist | - Retrospective, non-randomized design → confounding by indication likely - Unequal sample size (162 vs 42) → unstable estimates for cilta-cel subgroups and rare toxicities - Different follow-up duration (shorter for cilta-cel) → biased OS/late toxicity comparisons - Manufacturing/turnaround time differs (ide-cel faster, cilta-cel slower) → selection of fitter/stable patients and potential timing-related bias - Center heterogeneity (bridging therapy, admission, prophylaxis, grading, supportive care) → measurement and performance bias - Response assessment not centrally adjudicated; “investigator discretion” → outcome misclassification risk - Baseline imbalance signals (e.g., extramedullary disease higher in cilta-cel) + unmeasured factors (tumor burden, infection history, frailty) - Race/ethnicity representation limited (especially cilta-cel all White) → limited generalizability |
| *Nath, Shekarkhand et al. 2024;* | Real-world | CASP Cohort Study Checklist | - Retrospective, single-center design → limited generalizability, incomplete capture of infections. - Confounding by indication: treatment choice (CAR-T vs BsAb vs ADC) likely linked to patient fitness, disease status, prior therapies. - Heterogeneous exposures: commercial + investigational products pooled within each class; dosing schedules vary (especially BsAb). - Different treatment eras and changing prophylaxis/testing over time → time-trend bias. - Outcome ascertainment bias: CAR-T patients often monitored more intensively early; viral PCR testing not uniform → differential detection. - Short/variable follow-up for infection endpoints; long-term infection burden may be underestimated. - Prior therapy crossover (some BsAb patients had prior CAR-T); authors did sensitivity analyses, but residual impact possible. |
| *Trando, Ghamsari et al. 2024;* | Real-world | CASP Cohort Study Checklist | - Retrospective, single-institution design. - Very small cohort → low power; wide uncertainty. - No control group → descriptive only; no comparative effectiveness. - Selection by clinical judgment → strong confounding by indication - Heterogeneous prior therapies (including prior BCMA exposure) → residual confounding. - Non-uniform response confirmation (some VGPR without marrow). - Infection/CMV outcomes depend on local surveillance & prophylaxis (IVIg, PCR frequency, treatment thresholds). - Limited ability to identify predictive biomarkers (negative findings likely underpowered). |
| *Sidana, Ahmed et al. 2025;* | Real-world | CASP Cohort Study Checklist | - Observational registry design; not randomized. - Infused-patient only → survivorship/attrition bias before infusion. - Variable center practices (bridging, prophylaxis, monitoring) affect outcomes. - Potential under-reporting/misclassification of infections and toxicities. - Short/medium follow-up for late events (durability, late neurotoxicity, SPM). - Residual confounding in prognostic factor analyses. |
| *Kalariya, Hildebrandt et al. 2024;* | Real-world | CASP Cohort Study Checklist | - Retrospective design → incomplete capture of events, documentation variability. - Selection bias → clinicians may choose fitter older patients for ide-cel - No randomization → residual confounding despite adjustment. - Heterogeneous supportive care across 5 centers (IVIG, G-CSF, infection monitoring). - Frailty tool simplified (Facon scale) → may misclassify frailty; age heavily weighted. - Missing data (e.g., ECOG/CCI → some not evaluable for frailty) - Response assessment not fully standardized (e.g., confirmatory testing/imaging for CR/EMD not mandated). - Small subgroup (≥70 years) → limited power for age-stratified conclusions - Bridging therapy and prior treatments may differ and influence outcomes. |
| *Dima, Davis et al. 2024* | Real-world | CASP Cohort Study Checklist | - Retrospective design → selection and information bias - No control group → no causal or comparative inference - Short follow-up → durability of response and late infections unclear - Residual confounding despite multivariable adjustment - Heterogeneous practice (step-up schedule, supportive care across centers) - Response assessment non-central (CR/EMD confirmation not mandatory) - Small subgroups (prior BCMA, EMD) → limited precision |
| *Xia, Sun et al 2025* | Phase I/II | CASP Cohort Study Checklist | - Single-arm, open-label phase 2 → no comparator; no causal or comparative conclusions - Single-centre → center-specific practice and performance bias - Small sample size (n=37) → limited precision; rare toxicities may be missed - Highly selected population (post–BCMA CAR-T, age ≤70, fit patients) → selection bias - Short–moderate follow-up → long-term durability, late toxicities, and secondary malignancies unclear - As-treated analysis only → excludes screening/manufacturing failures - No central response review → potential outcome assessment bias - Exploratory endpoints → efficacy signals hypothesis-generating only |
| *Xu, Wang et al.* | Phase I/II | CASP Cohort Study Checklist | - Single-arm / no control group → no comparative or causal inference - Non-randomised, open-label → selection + performance bias likely - Small sample size → imprecise estimates; rare events may be missed - Heterogeneous treatment/supportive care (centres, conditioning, dosing) → confounding - Investigator-assessed outcomes (no central review) → measurement bias - Missing/incomplete baseline data (e.g., cytogenetics not for all) → limited adjustmen - Long follow-up with attrition → survivorship/attrition bias - Subsequent therapies after relapse → OS confounded, harder to attribute benefit |
| *Mi, Zhao et al. 2023* | Phase I/II | CASP Cohort Study Checklist | - Single-arm, open-label → no comparative or causal inference. - Selection bias: 16/64 enrolled never infused (PD/death/AEs/withdrawal) → infused cohort likely fitter. - Performance/management bias: supportive care and transfusion access influenced outcomes (some treatment-related deaths linked to limited transfusions). - Shorter maturity: median PFS/OS/DOR not reached → durability and late toxicities uncertain. - Small sample (n=48 infused) → imprecise subgroup estimates. - Generalizability limited: Chinese sites, different prior-therapy landscape, HBV context, no prior BCMA exposure allowed. |
| *Berdeja, Madduri et al. 2021;* | Phase I/II | CASP Cohort Study Checklist | - Single-arm, open-label → no comparator; no causal/comparative inference. - Selection bias / limited generalisability (ECOG 0–1, trial-eligible, specialised centres). - Short follow-up → durability, late relapses, late infections/secondary malignancies uncertain. - Potential reporting/assessment bias for subjective AEs in open-label setting. - Rare safety events (e.g., grade 5 CRS/HLH) → estimates unstable; limited power - Manufacturing/bridging attrition not fully captured in “treated” analysis → real-world effectiveness may be lower. |
| *Bahlis, Costello et al. 2023* | Phase I/II | CASP Cohort Study Checklist | - Single-arm phase 1 → no direct comparator; no causal/comparative inference. - Selection bias (trial-eligible, heavily pretreated; generalisability limited). - Dose/regimen heterogeneity (IV vs SC; sub-efficacious doses; priming/premedication introduced later) → mixed populations. - Short follow-up → long-term durability, late infections, and late immune toxicity uncertain. - Small numbers in key subgroups (prior BCMA therapy, EMD, high-risk cytogenetics) → imprecise estimates. - MRD data highly selected (only CR-or-better + clonotype-evaluable patients) → overestimates “MRD negativity rate”. - Discontinuations/AEs could bias efficacy if early stopping linked to toxicity/progression. |
| *Lesokhin, Tomasson et al. 2023* | Phase I/II | CASP Cohort Study Checklist | - Single-arm, open-label → no direct comparator or causal inference - Selection bias (trial-eligible, BCMA-naïve cohort only) - Short/immature follow-up → long-term durability and late infections uncertain - High censoring for time-to-event endpoints - Subgroup analyses underpowered (EMD, high-risk cytogenetics) - MRD analysis restricted to ≥CR and evaluable patients → optimistic rates - Supportive care variability (prophylaxis evolved during trial) |
| *Minakata, Ishida et al. 2023 – Phase 2* | Phase I/II | CASP Cohort Study Checklist | - Single-arm, open-label → no causal or comparative inference - Very small sample size (n=9) → imprecise estimates, unstable rates - Subgroup analysis only → not powered, descriptive results - Selection bias (trial-eligible, fitter patients) - Short follow-up → long-term durability, late toxicity unknown - Manufacturing/bridging therapy effects may influence outcomes - Limited generalizability beyond Japanese CAR-T centers |
| *Chari, Touzeau et al. 2025;* | Phase I/II | CASP Cohort Study Checklist | - Single-arm, open-label → no causal or comparative inference - Selection bias (trial-eligible, fitter patients; screen failures) - Residual confounding in post-hoc subgroup analyses - Heterogeneous prior therapies (CAR-T vs bispecific; timing effects) - Subjective AEs (taste changes) → measurement uncertainty - Limited generalisability (demographics, specialised centres) |
| *Moreau et al N Engl J med 2022;* | Phase I/II | CASP Cohort Study Checklist | - Single-arm design → no direct comparison to standard of care - Open-label → potential reporting/management differences (especially supportive care) - Selection bias (trial-eligible, relatively fit patients; strict inclusion/exclusion) - Confounding by prior therapies (heterogeneous prior regimens and disease biology) - Follow-up still limited for long-term OS, late toxicities, and durability - High infection/cytopenia burden may be influenced by site-specific prophylaxis practices - Subgroup/MRD analyses may be underpowered or affected by missing evaluability |
| *San-Miguel, Dhakal et al.2023* | Phase III | CASP Randomised Controlled Trial Checklist | - Open-label → potential bias in AE reporting, supportive care intensity, and symptom endpoints - Treatment timing/logistics bias (manufacturing delay + bridging/lymphodepletion only in cilta-cel arm) - Early events imbalance (more progressions/deaths in first 0–8 weeks in cilta-cel arm before infusion) - Non-standard primary analysis (weighted log-rank to handle bridging period; assumptions may affect interpretation) - Not all randomized patients received cilta-cel (attrition before infusion → dilutes/complicates ITT vs as-treated) - Control arm limited to DPd/PVd (other effective triplets not included at trial start → comparator may not reflect current options everywhere) - OS immature at interim analysis - Generalizability limits (ECOG ≤1, no prior BCMA-directed therapy; access to CAR-T differs by setting) |
| *Hill, Martens et al. 2024* | Phase I/II | CASP Cohort Study Checklist | - Observational (non-randomised) design → no causal inference - Vaccine timing not assigned → confounding by indication / calendar time - Vaccination schedule not standardised (doses, intervals, boosters) - Co-interventions (immunosuppression, IVIG, Evusheld) may distort immune readouts - Neutralising/T-cell testing only in a subgroup → lower power/selection bias - Anti-S IgG assay ceiling (2500 U/mL) → limited discrimination at high titers - CAR-T cohort small → imprecise subgroup estimates - Immunogenicity is a surrogate; infections likely under-detected |
| *Portuguese, Liang et al. 2025;* | Phase I/II | CASP Cohort Study Checklist | - manufacturing failures). - Exposure misclassification risk (EMD definition) → imaging-based; biopsy confirmation only in a minority; timing/activity may vary. - Confounding by disease severity → EMD likely correlates with higher tumor burden/inflammation; may drive toxicity independent of EMD itself. - Treatment heterogeneity → ide-cel vs cilta-cel; differing bridging regimens and supportive care may affect toxicity/infection outcomes. - Residual confounding → important variables may be unmeasured (frailty, comorbidity, infection prophylaxis intensity, prior therapies). - Outcome assessment variability → toxicity grading depends on documentation; retrospective grading can misclassify CRS/ICANS/eICAHT. - Time-related bias → long study period (2021–2024): evolving management (steroids/anakinra/G-CSF, admission thresholds) can change toxicity rates. - Multiple comparisons → many endpoints tested; risk of chance findings if not adjusted consistently. - Limited mechanistic data → no CAR-T expansion/persistence pharmacokinetics; harder to link biology to toxicity. |
| *Hansen, Peres et al. 2025* | Phase I/II | CASP Cohort Study Checklist | - Retrospective, non-randomized design limits causal inference. - Treatment allocation not random, raising concern for selection bias. - Residual confounding remains possible despite IPTW adjustment. - Differences in treatment era (cilta-cel approved later) may influence outcomes. - Incomplete capture of patients who progressed or died before infusion. - Short and variable follow-up duration, particularly for overall survival. - Outcome assessment reliant on chart review, introducing measurement variability. - No patient-reported outcomes or quality-of-life data included. - Rare toxicities show wide confidence intervals, limiting precision. |
| *Logue, Peres et al. 2022* | Phase I/II | CASP Cohort Study Checklist | - Severe cytopenias were almost universal early: Grade ≥3 cytopenias peaked around day 7 after ide-cel (about 94% of patients), then improved but persisted in a substantial proportion by day 30 and day 90 (notably thrombocytopenia). - Supportive care use was common: Most patients required G-CSF, many needed RBC/platelet transfusions, and a subset received TPO agonists, IVIG, or CD34+ stem cell boosts. - Infections were frequent but time-dependent in pattern: Roughly half of patients developed an infection within 100 days; early (≤30 days) infections were mainly bacterial and more often severe, while later (31–100 days) infections included more viral events and tended to be less severe. - Potential predictors/signals: Higher pre–CAR-T marrow myeloma burden, circulating plasma cells, and baseline severe anemia/cytopenia were associated with prolonged severe cytopenias; longer time from last bridging therapy to lymphodepletion was associated with infection risk. - Clinical take-away: In the SOC setting, monitoring and proactive supportive care for cytopenias and infections are central in the first 3 months post–ide-cel. |
| *Hansen, Sidana et al. 2023* | Phase I/II | CASP Cohort Study Checklist | - Retrospective observational design (non-randomized, non-interventional) - No concurrent control group (comparisons mainly against historical KarMMa data) - Short median follow-up (~6 months) → limited maturity for PFS/OS and late toxicities - Center-to-center heterogeneity in toxicity management, supportive care, infection prophylaxis, and bridging therapy - Response assessment not centrally reviewed (investigator-assessed; no blinded independent review - Confirmatory assessments not mandated (e.g., imaging/confirmatory testing for CR in EMD; MRD methods varied) - Missing data / variable assessment scedules inherent to real-world retrospective data captur - Multiple comparisons without adjustment → increased risk of false-positive findings in exploratory analyses - Potential selection/attrition along the pathway (apheresis → manufacturing → infusion) may influence outcome estimates |
| *Frenking, Riedhammer et al. 2025* | Phase I/II | CASP Cohort Study Checklist | - Retrospective observational design → no causal inference - No concurrent control group → effect size vs alternatives unclear - Selection bias (only patients who received talquetamab; access/fitness/referral influence) - Confounding by indication (sicker/healthier patients treated differently; prior therapies drive outcomes) - Heterogeneous practice patterns (dose/interval modifications, toxicity management vary by center/physician) - Missing data / loss to follow-up (external care, incomplete records) - Outcome misclassification risk (retrospective IMWG adjustments; imaging/confirmatory tests not mandatory) - Short follow-up (~8 months) → limited long-term PFS/OS, late infections, late toxicities - Trial comparison is indirect (different populations and follow-up; not intended for clinical guidance) |
| *Akhtar, Oloyede et al. 2025;* | Phase I/II | CASP Cohort Study Checklist | - Real-world CIBMTR registry cohort evaluating outcomes after idecabtagene vicleucel (ide-cel) - Focus on older (≥70 years) and frail patients with relapsed/refractory multiple myeloma - Overall survival and progression-free survival were comparable between older and younger patients - Frailty, rather than chronological age, was associated with inferior outcomes - Rates of CRS and ICANS were not meaningfully higher in older patients - Non-relapse mortality remained low across age groups - Study supports careful patient selection, not age alone, for CAR-T eligibility - Provides important real-world evidence beyond clinical trial |
| *Hashmi, Hansen et al. 2024* | Phase I/II | CASP Cohort Study Checklist | - Retrospective, observational design → susceptible to selection bias and unmeasured confounding; no causal inference. - Multicenter real-world data → variability in local practice (bridging therapy, supportive care, follow-up intensity). - Outcome assessment not centrally adjudicated → potential measurement/detection bias in response and progression. - Small numbers in key subgroups (e.g., plasma cell leukemia) → wider confidence intervals; less precise estimates. - Missing/incomplete baseline data for some variables (e.g., cytogenetics in a subset) → potential misclassification. - Confounding by indication likely (bridging therapy and prior BCMA therapy may reflect more aggressive disease/tumor burden). - Limited biomarker depth → important predictors (e.g., soluble BCMA, PET metabolic tumor volume, CAR-T expansion/persistence metrics) not available - Generalizability constraints → 11 US academic centers; may not fully represent community settings or other health systems. - Shorter effective follow-up for “early progression” focus → strong for the ≤3-month question, but less informative for long-term outcomes. |
| *Einsele, San-Miguel et al 2026* | Phase III | CASP Randomised Controlled Trial Checklist | - Open-label design → potential bias in AE reporting, supportive care intensity, infection monitoring, and patient-reported symptom endpoints. - Treatment logistics differ substantially between arms: cilta-cel required apheresis, bridging therapy, lymphodepletion, and delayed infusion, whereas standard therapy was started continuously. - Early events before CAR-T infusion may complicate intention-to-treat interpretation and attribution of infectious toxicity. - Infection outcomes were reported by CTCAE grade, which strengthens interpretability; however, duration of infections was not systematically reported. - Supportive care differed markedly, including substantially higher IVIG use in the cilta-cel arm, which may influence infection rates and limits direct comparison. - Control arm was restricted to DPd/PVd and may not fully reflect all current standard options. - Generalizability is limited to relatively fit patients with ECOG 0–1 and no prior BCMA-directed therapy. - Longer follow-up improves interpretation of OS and late safety, but very late infections and long-term immune reconstitution remain incompletely characterized. |
| *Dhakal, Akhtar et al 2025* | Real-World | CASP Cohort Study Checklist | - Retrospective multi-center design → selection and documentation bias possible. - No randomized comparator group → descriptive feasibility/safety data only, no causal inference. - Highly selected population intended for CAR-T → survivorship/selection bias. - Heterogeneous CAR-T products used after bridging: cilta-cel and ide-cel. - Supportive care, infection monitoring, and toxicity management likely varied across 20 centers. - Short bridging duration and limited follow-up → late infections and delayed toxicities may be underestimated - Infection reporting limited; only grade ≥3 infections after CAR-T are highlighted. - Very relevant real-world population, including many trial-ineligible patients, but generalizability depends on CAR-T access and center expertise. |
| *Kumar, Mateos et al 2026* | Phase II | CASP Cohort Study Checklist | - Clearly focused clinical question: efficacy and safety of talquetamab + teclistamab in extramedullary RRMM. - Highly selected cohort with aggressive extramedullary disease treated in a clinical trial setting. - Exposure/intervention well defined and protocol driven. - Outcomes assessed systematically using functional imaging and standardized toxicity grading. - Infection reporting clinically relevant but microbiological details limited. - No comparator arm → major limitation for interpretation of efficacy and toxicity. - Important confounders such as prior therapies and disease biology acknowledged but not fully adjusted for. - Median follow-up of 12.6 months may underestimate late infections and delayed toxicities. - Confidence intervals reported, supporting moderate statistical precision. - Results clinically meaningful given the poor prognosis of extramedullary MM. - Generalizability mainly limited to high-risk tertiary-care RRMM populations. |
| *Razzo, Midha et al 2026* | Real-World | CASP Cohort Study Checklist | - Clearly focused study question evaluating real-world safety and efficacy of teclistamab in RRMM patients. - Large multicenter retrospective cohort from 15 US academic centers improves external validity. - Cohort recruitment appropriate for real-world analysis, although tertiary referral-center bias likely. - Majority of patients (89%) would have been ineligible for MajesTEC-1, increasing clinical relevance. - Exposure/intervention clearly defined as commercial or expanded-access teclistamab therapy. - Outcomes clinically meaningful and systematically reported (ORR, CRS, ICANS, infections, PFS, OS). - Important confounders identified, including prior BCMA therapy, lymphopenia, cytopenias, ferritin, and disease burden. - Multivariable analyses performed to address confounding, strengthening interpretation. - Retrospective design with non-standardized supportive care and toxicity management across institutions. - Infection reporting clinically relevant, but microbiological details and infection duration incompletely characterized. - Response and toxicity assessment performed locally without central review. - Missing data for several prognostic variables and laboratory parameters. - Median follow-up of 10.1 months may underestimate late infections and long-term toxicity. - Results clinically applicable to real-world teclistamab practice. |
| *Schinke, Rodriguez-Otero et al 2025* | PhaseI/II | CASP Cohort Study Checklist | - Clearly focused study question evaluating infections and humoral immunity during talquetamab treatment in RRMM. - Cohort recruitment appropriate for heavily pretreated triple-class exposed RRMM patients. - Intervention/exposure clearly defined with protocol-driven talquetamab dosing schedules. - Outcomes systematically assessed using CTCAE grading and longitudinal immune monitoring. - Important infection-related outcomes reported, including timing, severity, opportunistic infections, hospitalization, and infection-related mortality. - Relevant biological correlates assessed, including B-cell counts, neutrophils, and polyclonal IgG levels. - No comparator arm, limiting causal interpretation versus BCMA-directed therapies. - Potential confounding by prior T-cell redirection therapy and heterogeneous supportive care practices across institutions. - Median follow-up adequate for early infectious complications but may underestimate long-term immune effects. - Infection prophylaxis, IVIG administration, and growth-factor support varied according to institutional practice. - Results clinically relevant - Generalizability mainly applies to fit RRMM patients eligible for clinical trial participation. |
| *Cani, Scott et al 2025* | Real-World | CASP Cohort Study Checklist | - Clearly focused study question - Retrospective single-center cohort - Cohort recruitment appropriate for a real-world RRMM - Exposure/intervention clearly defined (teclistamab, elranatamab, talquetamab). - Infection outcomes systematically graded using CTCAE criteria. - Clinically important infectious endpoints reported - Relevant biological correlates assessed - Important confounders identified, including steroid exposure, IVIG use, dosing interval modifications, and CRS/ICANS treatment. - Retrospective design and short follow-up may underestimate late or mild infections. - Single-center tertiary-care setting limits generalizability. - Mild infections diagnosed outside the institution may not have been captured completely. |
| *Al Hadidi, Szabo et al 2025* | Real-World | CASP Cohort Study Checklist | - Clearly focused study question - Cohort recruitment appropriate - Exposure/intervention clearly defined - Outcomes clinically relevant and systematically reported - Infection characteristics comprehensively described - Important confounders identified. No multivariable analysis for infection predictors - Retrospective design; institutional variability in IVIG and antimicrobial prophylaxis may influence infection outcomes. - Response and toxicity assessments performed locally without central review. - Median follow-up relatively short - Clinically important findings - Generalizability mainly applicable to tertiary-care RRMM Overall moderate |
| *Lee, Zonder et al 2025* | Phase I/II | CASP Cohort Study Checklist | - Clearly focused study question - Recruitment appropriate - Intervention clearly defined - Outcomes are clinically relevant and systematically reported - Safety outcomes comprehensively described - Confounders identified, no comparator - Prospective design, variability in prophylactic management - Follow-up short, long-term effects might be missed. - Generalizability mainly applies to fit RRMM patients eligible for clinical trial participation. |
| *Bros, Harel et al 2025* | Real-World | CASP Cohort Study Checklist | - Clearly focused study question - Retrospective single cohort design - Recruitment appropriate - Intervention clearly defined - Relevant safety outcomes assessed - Important confounders identified - No multivariable analyses, no comparator - Follow-up short - Generalizability mainly applies to heavily pretreated RRMM patients |
| *Soltantabar, Hibma et al 2026* | Phase I/II | CASP Cohort Study Checklist | - Clearly focused study question - Pooled analysis of prospective trial cohorts - Recruitment appropriate - Intervention clearly defined - Relevant safety outcomes assessed - Important confounders identified - Incorporated multivariable analyses, no comparator - Heterogenicity in prophylactic management - Adverse events systematically assessed - Follow-up short - Generalizability mainly applies to heavily pretreated RRMM patients |
| *Snyder, Atrash et al 2025* | Real-World | CASP Cohort Study Checklist | - Clearly focused study question. - Multicenter retrospective real-world cohort study. - Cohorts appropriately recruited from heavily pretreated RRMM patients treated with teclistamab. - Intervention/exposure clearly defined - Relevant clinical outcomes assessed - Important confounders identified - No randomization - Fixed-duration cohort highly selected and substantially smaller than continuous cohort (16 vs. 72 patients). - Retrospective design with heterogeneous follow-up and supportive care strategies. - Infection analyses were descriptive and not systematically adjudicated. - Short median follow-up - Generalizability mainly applicable to heavily pretreated RRMM populations. - Study provides important exploratory real-world evidence regarding response-adapted teclistamab strategies. |
| *Stork, Radocha et al 2025* | Real-World | CASP Cohort Study Checklist | - Clearly focused study question. - Multicenter retrospective real-world cohort study. - Cohorts appropriately recruited from heavily pretreated RRMM patients treated with teclistamab. - Intervention/exposure clearly defined (weekly vs. reduced-frequency dosing). - Relevant clinical outcomes assessed (PFS, OS, infections, hematologic toxicity). - Important prognostic/confounding factors identified (frailty, response status, EMD, high-risk cytogenetics). - No randomization. - Dose de-escalation mainly performed in responding or frailer patients, introducing selection/confounding bias. - Non-weekly cohort substantially smaller than weekly cohort (18 vs. 55 patients). - Retrospective design with heterogeneous supportive care and IVIG prophylaxis strategies. - Infection analyses largely descriptive and not systematically adjudicated. - Short median follow-up (4.9 months) with immature OS data. - Limited statistical power due to small cohort size. - Generalizability mainly applicable to heavily pretreated RRMM populations receiving real-world teclistamab. - Study provides important exploratory real-world evidence supporting response-adapted teclistamab dose de-escalation strategies. |
| *Dima, Loque et al 2026* | Real-World | CASP Cohort Study Checklist | - Clearly focused study question evaluating cytopenias and infections after standard-of-care cilta-cel in RRMM. - Multicenter retrospective real-world cohort study. - Consecutive heavily pretreated RRMM patients appropriately recruited after ≥4 prior lines of therapy. - Exposure/intervention clearly defined - Relevant clinical outcomes comprehensively assessed - Important confounders identified - No randomization or control group. - Retrospective design with heterogeneous institutional supportive care and antimicrobial prophylaxis strategies. - Infection assessment partly dependent on retrospective chart review and outside-hospital documentation. - Follow-up relatively short - Some analyses underpowered - Outcomes were clinically relevant and objectively defined. - Study provides important real-world evidence - Generalizability mainly applicable to heavily pretreated RRMM patients treated with commercial BCMA CAR-T therapy. |
| *Yi, Lee et al 2025* | Real-World | CASP Cohort Study Checklist | - Clearly focused study question - Cohort appropriately recruited - Intervention/exposure clearly defined - Relevant clinical outcomes assessed - Important prognostic/confounding factors identified - No randomization or control group. - Small cohort size (42 patients) limits statistical power. - Retrospective design with heterogeneous institutional prophylaxis and supportive care strategies. - Infection analyses limited by variability in prophylaxis protocols and retrospective AE documentation. - Follow-up duration relatively moderate - Objective response and toxicity criteria used (IMWG and ASTCT criteria). - Generalizability mainly applicable to heavily pretreated Asian RRMM populations treated outside clinical trials. |
| *Wang, Li et al 2026* | Real-World | CASP Cohort Study Checklist | - Clearly focused study question - Multinational retrospective cohort study - Cohorts appropriately - Intervention/exposure clearly defined - Relevant clinical outcomes assessed - Important confounders partially addressed through matching - Residual confounding likely remained because important disease-specific variables were unavailable. - No randomization or prospective treatment allocation. - Retrospective design based on electronic health records and coding algorithms. - Safety outcomes partly dependent on ICD coding and laboratory-derived definitions without central adjudication. - TTNT used as surrogate for PFS and potentially influenced by physician practice patterns. - Follow-up adequate for short-/mid-term outcomes but limited for long-term toxicity interpretation. - Large matched cohort (188 vs 188 patients) improved statistical robustness. - Generalizability high for real-world RRMM patients treated with BCMA bispecific antibodies. - Study provides important comparative real-world evidence |
| *Jelinek, Zihala et al 2026* | Real-World | CASP Cohort Study Checklist | - Clearly focused study question - Multicenter translational cohort study combining clinical real-world data with immune profiling analyses. - Cohorts appropriately recruited - Exposure/intervention clearly defined - Relevant clinical and biological outcomes assessed - Important confounders identified - No randomization or prospective treatment allocation. - Retrospective clinical data collection with heterogeneous supportive care and prophylaxis strategies between centers. - Several subcohorts relatively small, limiting statistical power for subgroup analyses. - Infection reporting based partly on retrospective chart review. - Advanced and standardized laboratory methods used - Longitudinal immune monitoring - Mechanistic findings biologically plausible - Generalizability mainly applicable to heavily pretreated RRMM patients receiving bispecific antibodies - Study provides important mechanistic evidence |
| *Hansen, Dima et al 2026* | Real-World | CASP Cohort Study Checklist | - Clearly focused study question. - Large multicenter registry-based cohort. - Cohort appropriately recruited. - Intervention/exposure clearly defined. - Relevant clinical outcomes assesse. - Important prognostic factors identified and adjusted for in multivariable analyses. - No randomization or comparator group. - Standardized outcome definitions used. - Some clinically relevant variables were unavailable or incompletely captured. - Median follow-up of 12 months limits assessment of long-term efficacy, infections, and secondary malignancies. - Infection data were collected, but detailed pathogen-specific analyses and prophylaxis strategies were not systematically available. - Generalizability is high for real-world RRMM patients - Study provides important real-world evidence |
| *Piron, Rodrigo et al 2025* | Real-World | CASP Cohort Study Checklist | - Clearly focused study question. - Single-center retrospective real-world cohort study. - Consecutive RRMM patients were appropriately recruited. - Exposure/intervention clearly defined. - Relevant clinical outcomes assessed. - Important confounders identified. - No randomization. - Historical control design introduces risk of temporal bias due to evolving supportive care and physician experience. - Most baseline characteristics were balanced between groups. - Appropriate statistical analyses performed. - Small sample size (n=66) and single-center design. - Follow-up restricted to 9 months. - Study provides important real-world evidence. |
| *Portuguese, Davis et al 2026* | Real-World | CASP Cohort Study Checklist | - Cllearly focused study question. - Exposure/intervention clearly defined. - Relevant clinical outcomes assessed. - Important confounders identified and addressed in multivariable analyses. - No randomization or comparator group. - Retrospective design with heterogeneous supportive care. - Large proportion of frail and trial-ineligible patients. - Appropriate statistical methods used. - Missing data for some variables. - Median follow-up is relatively short. - Infection outcomes were systematically analyzed. - Generalizability is high for heavily pretreated RRMM patients. - Study provides important real-world evidence. |
| *Smits, Groen et al 2026* | Real-World | CASP Cohort Study Checklist | - Clearly focused study question. - Single-center retrospective real-world cohort study. - Cohort appropriately recruited- - Exposure/interventions clearly defined. - Relevant clinical outcomes assessed. - Important confounders identified. No randomization. - Historical changes in supportive care may have influenced outcomes. - Infection assessment was comprehensive. - Use of annualized infection rates provided a more accurate estimate of infectious burden than cumulative incidence alone. - Dose de-escalation analyses are potentially affected by survivor bias. - Small sample size (n = 80) and single-center design limit external validity. - Retrospective data collection introduces risk of incomplete ascertainment and residual confounding. - Long median follow-up (21 months) allowed assessment of recurrent infections and late infectious complications. - Study provides important real-world evidence. |
| *Bilbao, Anson et al 2025* | Real-World | CASP Cohort Study Checklist | - Clearly focused study question. - Single-center retrospective observational cohort study. - Cohort appropriately recruited. - Exposure/intervention clearly defined. - Relevant infection-related outcomes assessed. - Important risk factors explored. - No randomization and no comparator group. - Small number of infection events limits statistical power. - Objective outcome assessment. - IVIG use was more frequent among infected patients. - Single-center setting limits external validity and generalizability. - Study provides important hypothesis-generating evidence that Campylobacter spp. may represent an emerging bacterial complication in patients receiving BCMA- and GPRC5D-directed bispecific antibodies. |
| *Ssentongo, Guare et al 2026* | Real-World | CASP Cohort Study Checklist | - Clearly focused study question. - Single-center retrospective real-world cohort study. - Cohort appropriately recruited. - Exposure/intervention clearly defined. - Relevant clinical outcomes assessed. - Potential risk factors explored. - No randomization and no comparator group. - Very small sample size (n = 19) - Heterogeneous prophylaxis practices. - Infection definitions were rigorous. - Outcome assessment was clinically relevant - No standardized infection - Follow-up was relatively short (median 196 days), - Study provides supportive real-world evidence |
| *Cheema, Shrestha et al 2026* | Real-World | CASP- Cohort Study Checklist | - Clearly focused study question. - Single-center retrospective real-world cohort study. - Cohort appropriately recruited. - Exposure/intervention clearly defined (teclistamab therapy). - Relevant clinical outcomes assessed. - Potential risk factors explored. - No randomization and no comparator group. - Relatively large sample size (n = 177). - Nearly universal CMV surveillance (97.7% tested). Objective PCR-based definition of CMV reactivation. - Multivariable analysis performed. - Outcome assessment was clinically relevant. - Variable CMV monitoring frequency. - Retrospective design with potential residual confounding. - Single-center setting may limit generalizability. - No standardized prophylactic CMV strategy. - Follow-up was sufficient to capture most reactivation events. - Study provides important real-world evidence |
| *Tan, Usmani et al 2026* | Real-World | CASP-Cohort Study Checklist | - Large international multicenter retrospective real-world cohort study. - Cohort appropriately recruited (441 patients from 7 countries). - Exposure/intervention clearly defined. - Relevant clinical outcomes assessed. - Potential risk factors explored. - No randomization and no comparator group. - Large sample size (n = 441). - Real-world population included many trial-ineligible patients. - Multivariable Cox regression performed. - Early mortality was objectively defined and measured. - Cause of death was investigator-reported and may be subject to misclassification. - Supportive care and prophylaxis practices were heterogeneous across centers. - Some baseline variables had incomplete data. - Follow-up was adequate (median follow-up 17.1 months). - Study provides important real-world evidence. |
| *Little, Pena et al 2025* | Real-World | CASP Cohort Study Checklist | - Clearly focused study question. - Retrospective two-center real-world cohort study. - Cohort appropriately recruited. - Exposure/intervention clearly defined. - Relevant infectious outcomes assessed. - Standardized EORTC/MSGERC definitions used. - Clinically relevant outcomes assessed. - No randomization and no comparator group. - Large cohort size for this specific research question (n = 234). - Comprehensive follow-up up to 1 year. - Autopsies were reviewed to identify potentially missed fungal infections. - Very low event rate (only 4 IFD cases), limiting statistical power. No formal risk factor analysis possible because of the low number of events. - Antifungal prophylaxis practices differed between centers. - Retrospective design with potential residual confounding. - Published as a research letter with limited methodological detail. Study provides important real-world evidence. |
| *Yan, Du et al 2026* | Phase II | CASP Cohort Study Checklist | - Clearly focused study question. - Prospective phase II, open-label, single-arm trial. - Clearly defined inclusion criteria and study population. - Intervention and treatment protocol clearly described. - Relevant clinical and safety outcomes assessed. - Objective primary endpoint (MRD negativity at 10⁻⁵). - Standardized assessment. Outcome assessment was objective. - No randomization and no comparator/control group. - Small sample size (36 infused patients). - Follow-up relatively short (median 15.8 months). - Limited ability to assess comparative effectiveness or causality. - Potential selection bias due to inclusion of transplant-ineligible/non-transplanted NDMM patients. - Study provides important proof-of-concept evidence for frontline BCMA CAR-T therapy in NDMM. |
| *Cohen, Lebel et al 2026* | Real-World | CASP Cohort Study Checklist | - Clearly focused study question. - Single-center cohort study. Cohort appropriately recruited. - Exposure/intervention clearly defined. Relevant infectious outcomes assessed - Prospective viral monitoring with predefined PCR surveillance - Objective outcome assessment using PCR-based viral detection. Clinically significant viral reactivation was predefined. - Associations with survival outcomes (PFS, OS) and CAR-T toxicities were evaluated. - No randomization and no dedicated control group. - Single-center design may limit generalizability. - Sample size was relatively small (n = 75 MM patients). - Few clinically significant CMV events limited statistical power. - Potential confounding from relapse and subsequent therapies. - Follow-up was adequate. - Outcome assessment was rigorous and clinically relevant. - Study provides important real-world/prospective evidence. |
| *Bar, Martin et al 2026* | Phase I | CASP Cohort Study Checklist | - Clearly focused study question. - First-in-human, open-label phase I dose-escalation and expansion study. - Well-defined RRMM patient population (≥3 prior lines). - Intervention clearly described (IV and SC alnuctamab). - Relevant efficacy and safety outcomes assessed. - Objective outcome assessment using IMWG response criteria and standardized AE grading. - Clinically relevant infectious outcomes reported. - Infection mitigation strategies and prophylaxis measures clearly described. - No randomization and no comparator/control group. - Small sample size typical of an early-phase study. - Potential selection bias due to trial eligibility criteria. - Median follow-up relatively short (11.8 months). - Industry-sponsored study. - Long-term efficacy and infection risk remain uncertain. - Results may not be fully generalizable to routine clinical practice. - Study provides important early evidence regarding efficacy |
| *Habib, Ahmed et al 2026* | Real-World | CASP Cohort Study Checklist | - Clearly focused study question. Multicenter retrospective real-world cohort study.Cohort appropriately recruited. - Clearly defined exposur and comparison groups. - Relevant clinical outcomes assessed. - Outcomes measured using standardized criteria. - Real-world population included patients often excluded from pivotal trials. No randomization. - Potential confounding due to baseline differences between renal impairment and normal renal function cohorts. - Retrospective design with potential for incomplete documentation and reporting bias. - Small renal impairment subgroup (n = 25), limiting statistical power. - Infection outcomes were clinically relevant but not microbiologically characterized. - Follow-up was adequate (median 27.9 months). - Survival outcomes (PFS, OS) systematically assessed. - Missing information on immunoglobulin replacement and some supportive care measures. - Study provides important real-world evidence |
| *Janakiram, Tan et al 2026* | Real-World | CASP Cohort Study Checklist | - Clearly focused study question. Large international multicenter real-world cohort study. Cohort appropriately recruited Intervention clearly described. - Relevant efficacy, safety, and infection outcomes assessed. - Outcomes measured using standardized criteria. - Broad real-world population - Long follow-up (median 12.7 months). - Detailed characterization of infections - No randomization and no comparator group. - Retrospective design: potential selection and reporting bias. - Infection grading was inconsistently reported across centers. - Supportive care/ prophylaxis were not standardized. - Missing data for some variables - Potential confounding due to heterogeneous prior therapies and patient characteristics. - Results are highly relevant to routine clinical practice |
| *Lee, Plattel et al 2026* | Phase I | CASP Cohort Study Checklist | - Clearly focused study question and prospective multicenter phase 1/1b design. - Intervention, efficacy, safety, and infection outcomes clearly defined and assessed using standardized criteria. - Detailed reporting of bacterial, viral, fungal, opportunistic, and fatal infections. - Adequate follow-up (median 17.2 months). - No randomization and no comparator group. - Small sample size and early study termination. - Supportive care and infection prophylaxis were not fully standardized. - Results are clinically relevant for infection risk assessment in BCMA-directed bispecific antibody therapy |
| *Liu, Zhang et al 2026* | Real-World | CASP Cohort Study Checklist | - Clearly focused study question. - Large retrospective cohort of 147. - Relevant infection and immunologic outcomes assessed. - Longitudinal follow-up up to one year after CAR-T therapy. - No comparator group and retrospective design. - Potential selection bias and residual confounding. - Limited pathogen-specific infection data. - Results are highly relevant for understanding infection risk and immune reconstitution after BCMA CAR-T therapy. |
| *Costa, Bahlis et al 2025* | Phase III | CASP randomized controlled trial checklist | - Open-label design (no blinding) - Industry-sponsored trial - Independent review committee for primary endpoint assessment - Large randomized phase III study - Balanced baseline characteristics - Intention-to-treat analysis - Increased risk of serious and fatal infections - Follow-up limited for long-term safety assessment |
| *Mersi, Burow et al 2026* | Real-World | CASP Cohort Study Checklist | - Retrospective cohort design - Single-center study - No control group - Small sample size - Heterogeneous treatment cohorts - Potential selection bias - Potential confounding - Real-world clinical data - Comprehensive infection assessment - Routine IVIG prophylaxis may have influenced infection rates - Limited generalizability - Short-to-moderate follow-up for long-term outcomes |

**Supplementary Table 1:** This table presents the critical appraisal of the included studies using the Critical Appraisal Skills Programme (CASP) tools. Depending on the study design, either the CASP Cohort Study Checklist or the CASP Randomised Controlled Trial Checklist was applied to evaluate methodological quality. The key CASP items summarize the main potential sources of bias and methodological limitations identified in each study, including aspects such as study design, confounding, sample size, follow-up duration, and outcome assessment.

Abbreviations: CASP, Critical Appraisal Skills Programme; RCT, randomized controlled trial; AE, adverse event; CAR-T, chimeric antigen receptor T-cell therapy; BsAb, bispecific antibody; IVIG, intravenous immunoglobulin; CRS, cytokine release syndrome; ICANS, immune effector cell-associated neurotoxicity syndrome; allo-HCT, allogeneic hematopoietic cell transplantation; CNS, central nervous system; CSF, cerebrospinal fluid; OS, overall survival; PFS, progression-free survival; DOR, duration of response; SOC, standard of care; ECOG, Eastern Cooperative Oncology Group; EMD, extramedullary disease; MRD, minimal residual disease; GVHD, graft-versus-host disease; HLH, hemophagocytic lymphohistiocytosis.
