# Supplementary material for: Infectious toxicities associated with bispecific antibodies and CAR-T Cells in multiple myeloma: a systematic review
Source: Ann Hematol. 2026 Jun 18;105(9):397. doi: 10.1007/s00277-026-07140-8 (PMC13521976; doi:10.1007/s00277-026-07140-8)
Supplement: Supplementary file 3 [file 277_2026_7140_MOESM3_ESM.docx]

| Supplementary Table 3: comprehensive clinical summary  Supplementary Table 3.a: study outcomes on infections | | | | | | | | | | | | | | | |
| --- | --- | --- | --- | --- | --- | --- | --- | --- | --- | --- | --- | --- | --- | --- | --- |
| Study | **Hypogamma-globulinemia** | | **Infection**  **any grade**  **(3/4)** | | **Respiratory infection** | | **PJP** | **Viral** | **CMV** | **HSV/ VZV** | **bacteria** | **Fungal infection** | **Prophylaxis**  **PJP: P;**  **Anti-Viral: V; Fungal:F; IVIG: I; Bacterial: B** | **Death due to infection** | **Time to onset of any infection in months** |
| Teclistmab |  |  |  |  |  |  |  |  |  |  |  |  |  |  |  |
| *Moreau et al N Engl J med 2022;* | 74.5%  (<500mg/dl) | | 76.4% (44.8%) | | 14.5% Pneumonia 10.3% | | 3.6% | n.a. | n.a. | n.a. | 1.2% | n.a. | n.a. | n.a. | n.a. |
| *Usmani, Garfall et al. 2021;* | 9% | | 60% (22%) | | 11.5% Pneumonia 18.5% upper respiratory | | n.a. | n.a. | n.a. | n.a. | n.a. | n.a. | P- n.a.; V: 83%; F: 5%; I: 11%; B: n.a. | 7% | n.a. |
| *Nooka, Rodriguez et al. 2024;* | 71.5% (<400mg/dl) | | 80% (55.2%) | | 19.4% | | 4.2% | n.a. | 1.8% | 4.2% | 7.2% | 5.5% | P:32.1%; V:68.5%; F:n.a; I: 6.1%; B: n.a. | 12.7% | 1.7 m |
| *Dima, Davis et al. 2024;* | 42% (<400mg/dl.) | | 36.8% (17%) | | 18.9% viral | | n.a. | n.a. | n.a. | n.a. | 15.1% | 2.8% | n.a. | n.a. | n.a. |
| *Frerichs, Verkleij et al. 2024;* | n.a. | | 42.3% grade 3/ 4 | | 65% | | n.a. | n.a. | n.a. | n.a. | n.a. | n.a. | n.a. | n.a. | n.a. |
| *Riedhammer, Bassermann et al. 2024* | n.a. | | n.a. | | n.a. | | n.a. | n.a. | n.a. | n.a. | n.a. | n.a. | P:100%; V:100%; F: n.a; B: n.a.; I: n.a. | n.a. | n.a. |
| *Touzeau, Krishnan et al. 2024;* | n.a. | | 70% (42.5%) | | n.a. | | n.a. | n.a. | n.a. | n.a. | n.a. | n.a. | n.a. | n.a. | n.a. |
| *Kawasaki, Steele et al. 2024* | n.a. | | 25% | | n.a. | | n.a. | n.a. | n.a. | n.a. | n.a. | n.a. | n.a. | n.a. | n.a. |
| *Tan, Asoori et al. 2025;* | 69% | | 56.2% (22%) | | 19%, Pneumonia: 22.4% | | n.a. | 41% | 4% | n.a. | n.a. | n.a. | P: 100%; V: 100%; F: n.a.; I: 62%; B: n.a. | n.a. | 1.1 m |
| *Ariane, Stéphanie et al. 2025;* | 50% | | 54.2% | | 40.6% | | n.a. | n.a. | n.a. | n.a. | n.a. | n.a. | n.a. | n.a | n.a. |
| *Perrot, Hulin et al. 2024* | n.a. | | 29.9% | | n.a. | | n.a. | n.a. | n.a. | n.a. | n.a. | n.a. | I: 61.4% | n.a. | n.a. |
| *Ishida, Kuroda et al. 2025* | 84.6% | | 76.9% (19.2%) | | n.a. | | n.a. | n.a. | 11.5% | n.a. | n.a. | n.a. | n.a. | n.a. | n.a. |
| *Mohan, Monge et al. 2024;* | n.a. | | 40% (26%) | | 44.6% | | 2.7% | 45% viral | n.a. | n.a. | 48% | 6.7% | V: 99%; P: 58%; B: 12%; F: 14%; I: 43% | n.a. | 2 m |
| *Cai, Xia et al; 2024* | 92.3% | | 96.2% | | n.a. | | n.a. | n.a. | n.a. | n.a. | n.a | n.a. | I: 80.8% | n.a. | n.a. |
| *Razzo, Midha et al 2026* | 42$ | | n.a. | | n.a. | | n.a. | n.a. | n.a. | n.a. | n.a. | n.a. | I: 38% | 5% | n.a. |
| *Bros, Harel et al 2025* | n.a. | | n.a. | | n.a. | | n.a. | n.a. | n.a. | n.a. | 75% | n.a. | I: 68% | n.a. | n.a. |
| *Snyder, Atrash et al 2025* | n.a. | | Continuous 59%(22%); Fixed-duration: 75%(69%) | | n.a. | | n.a. | n.a. | n.a. | n.a. | n.a. | n.a. | n.a. | n.a. | n.a. |
| *Stork, Radocha et al 2025* | n.a. | | Weekly: 80.0% (29.1%) ; Non-weekly: 66.7% (50%) | | n.a. | | n.a. | n.a. | n.a. | n.a. | n.a. | n.a. | n.a. | n.a. | n.a. |
| *Yi, Lee et al 2025* | 50% | | 47.6% (42.9%) | | 14.3% Pneumonia; upper respiratory: 4.7% | | n.a. | COVID-19: 26.2% | n.a. | n.a. | n.a. | n.a. | V: 78.6%  F: 50.0%;  I: 50.0%;  B: 88.1% | 16.6% | n.a. |
| *Smits, Groen et al 2026* | 83% | | 90% (38%) | | 81% | | 1.3% | n.a. | n.a. | n.a. | n.a. | n.a. | I: 83% | 5% | Median time to first infection: 1.3 ; first severe infection: 6.5 m |
| *Ssentongo, Guare et al 2026* | n.a. | | 57.9% (78.9%) | | 36% | | 0%. | 47.4% | 10.5% | 0% | 52.6% | 0% | n.a. | n.a. | n.a. |
| *Cheema, Shrestha et al 2026* | n.a. | | n.a. | | n.a. | | n.a. | n.a. | 22% | n.a. | n.a. | n.a. | I: 61.6% | n.a. | n.a. |
| *Costa, Bahlis et al 2025* | 84.5% | | 96.5% (54.1%) | | Pneumonia: 16.6% | | n.a. | COVID-19: 43.8% | n.a. | n.a. | n.a. | n.a. | n.a. | n.a. | n.a. |
| Elranatamab |  |  |  |  |  |  |  |  |  |  |  |  |  |  |  |
| *Lesokhin, Tomasson et al. 2023* | 75.5% (<400mg/gl) | | 69.9% (39.8%) | | 16.3% | | 4.9% | n.a. | 5.7% | n.a. | 5.7% | 0% | P: 49.6%; V: 87%; F: 11.4%; I:43.1%; B: 5.7% | 3% | n.a. |
| *Bahlis, Costello et al. 2023* | n.a. | | 74.5% (26.8%) | | n.a. | | 3% | n.a. | 1.8% | n.a. | n.a. | n.a. | P:n.a.; V: n.a.; I:32.7%; B:n.a. | 3.6% | n.a. |
| *Tomasson, Iida et al. 2024* | n.a. | | n.a. | | n.a. | | n.a. | n.a. | n.a. | n.a. | n.a. | n.a. | n.a. | n.a. | n.a. |
| *Iida, Ito et al. 2024* | n.a. | | n.a. | | n.a. | | n.a. | n.a. | n.a. | n.a. | n.a. | n.a. | n.a. | n.a. | n.a. |
| *Malard, Bobin et al. 2024* | 49% (24%) | | 44% | | 12% | | 0% | 43.5%. | 10.8% | n.a. | 18.9% | 7% | n.a. | n.a. | n.a. |
| *Soltantabar, Hibma et al 2025* | n.a. | | n.a. | | 41.7% grade ≥3 | | n.a. | n.a. | n.a. | n.a. | n.a. | n.a. | n.a. | n.a. | n.a. |
| *Portuguese, Davis et al* | n.a. | | 38% (57%) | | n.a. | | n.a. | n.a. | n.a. | n.a. | n.a. | n.a. | I: 46% | n.a. | n.a. |
| Linvoseltamab |  | |  | |  | |  |  |  |  |  |  |  |  |  |
| *Bumma, Richter et al. 2024;* | n.a. | | 74.4% (25.9%) | | 18.8% | | 4.3% | 6% | n.a. | n.a. | Pneumonia 16.2% | 5.2% | n.a. | n.a. | 53% within the first 3 months |
| *Lee, Zonder et al 2025* | n.a. | | 75.2% (47.9%) | | 21.4% | | n.a. | COVID-19 22.2% | 5% | n.a. | n.a. | n.a. | n.a. | 7 infection related deaths | n.a. |
| AMG 420 |  |  |  |  |  |  |  |  |  |  |  |  |  |  |  |
| *Topp, Duell et al. 2020;* | n.a. | | 33% (10%) | | 14% pneumonia | | n.a. | n.a. | n.a. | n.a. | n.a. | 2% | n.a. | n.a. | n.a. |
| *Pavurutamab* |  | |  | |  | |  |  |  |  |  |  |  |  |  |
| *Lee, Plattel et al 2026* | n.a. | | 70.9% (30.8%) | | URTI 14.5%; Pneumonia 11% | | 1.2% | 26.7% | 4.1% | 0.6% | 29.1% | 5.4% | P: 33.1%  V: 69.8%  F: 9.3%  I: 37.2% | 4.1% | n.a. |
| Alnuctamab |  | |  | |  | |  |  |  |  |  |  |  |  |  |
| *Bar, Martin et al 2026* | 51.6% | | 64.2% (14.7%) | | URTI 14.7% | | 0% | 51.6% (COVID-19 23.2%) | 4% | n.a. | n.a. | 1% | I: 51.6% | 1% | n.a. |
| Talquetamab |  |  |  |  |  |  |  |  |  |  |  |  |  |  |  |
| *Chari, Minnema et al. 2022;* | 87% | | n.a. | | n.a. | | n.a. | n.a. | 0% | n.a. | n.a. | n.a. | V: 52.4% | n.a. | n.a. |
| *Schinke, Dhakal et al. 2024;* | n.a. | | n.a. | | n.a. | | n.a. | n.a. | n.a. | n.a. | n.a. | n.a. | n.a. | n.a. | n.a. |
| *Chari, Touzeau et al. 2025* | n.a. | | n.a. | | 4.9-5.8% | | 0% | n.a. | 1%. | n.a. | 4,5-8,4% | 2% | n.a. | n.a. | n.a. |
| *Schinke, Rodriguez et al 2025* | 57-67% | | 28-39% | | n.a. | | 2-8% | n.a. | n.a. | 2% VZV | n.a. | 12% non invasive candidiasis | P: 43%  V: 92%  B:n.a.; F: n.a.  IVIG 16% | 1.5% | Mostly within the first 100 days |
| *Frenking, Riedhammer et al. 2025;* | 50% | | 55.8% (16.7%) | | 24.6% | | n.a. | 5% of severe infections | 6.5% | n.a. | 18.8% | 2.9% | n.a. | 4.3% | n.a. |
| *Iida, Sunami et al 2025* | (6.7%) | | 53.3% | | n.a. | | n.a. | n.a. | n.a. | n.a. | n.a. | n.s. | P: 86.7%; V:100%:IVIG: 20%; B: 20% | n.a. | n.a. |
| *Dhakal, Akhtar et al 2025* | n.a. | | Grade ≥3 5% after CAR-T | | n.a. | | n.a. | n.a. | n.a. | n.a. | n.a. | n.a. | n.a. | n.a. | n.a. |
| *Janakiram, Tan et al 2026* | n.a. | | 51% (48%) | | URI 32.1%, pneumonia 12.4% | | n.a. | 12.4% | 11% | n.a. | 10.2% | n.a. | n.a. | 0 | n.a. |
| Cevostamab |  | |  | |  | |  |  |  |  |  |  |  |  |  |
| *Trudel, Cohen et al. 2021* | 45% | | 42.5% (18.8%) | | n.a. | | n.a. | n.a. | n.a. | n.a. | n.a. | n.a. | n.a. | n.a. | n.a. |
| *Lesokhin, Richter et al. 2022* | n.a. | | 12% | | n.a. | | n.a. | n.a. | n.a. | n.a. | n.a. | n.a. | n.a. | n.a. | n.a. |
| All bispecific antibodies | |  |  |  |  |  |  |  |  |  |  |  |  |  |  |
| *Jourdes, Cellerin et al. 2024* | n.a. | | 70% (53%) | | 63% | | 13% | n.a. | 13% | 6% | 56% | 5% | n.a. | n.a. | n.a. |
| *Li, Zhang et al. 2025;* | n.a. | | 100% (66.7%) | | n.a. | | n.a. | n.a. | n.a. | n.a. | n.a. | n.a. | Vaccination, n (%) 2 (22.2) | n.a. | 6 m  (2-10) |
| *Cohen, Magen et al. 2025* | n.a. | | Grade 3/ 4 infection 64% | | n.a. | | n.a. | n.a. | n.a. | n.a. | n.a. | n.a. | n.a. | n.a. | n.a. |
| *Hammons, Szabo et al. 2024* | n.a. | | BCMA: 58%  GPRC5d: 36% | | BCMA viral 39%  GPRC5d: 38% | | n.a. | n.a. | 4.5% | n.a. | BCMA: 54%  GPRC5d: 50% | BCMA 7%  GPRC5d: 12% | V: 100%; P: 0%; F: 0%; I: ≤400 mg/dL | n.a. | n.a. |
| *Mohan, Szabo et al. 2025* | n.a. | | 73% | | n.a. | | 2.2% | n.a. | n.a. | n.a. | 53% | 2.7% | n.a. | 4.8% | 3.2 m |
| *Lancman, Parsa et al. 2023* | 100% | | 118 infections with 26 grade 3-5 infections | | Upper resp. infec.:33%  Lower resp. infec.: 22% | | 2.7% | 46% | 22% | n.a. | 43% | 11% | n.a. | n.a. | 3.8 (grade 3-5: 18.7) |
| *Uttervall, Tätting et al. 2024* | 53% | | 32.7% | | 3.4% | | n.a. | n.a. | n.a. | n.a. | n.a. | n.a. | n.a. | n.a. | n.a. |
| *Sim, Longhitano et al. 2023* | 87% | | 90% (41%) | | 41% | | 0% | 58% | 18%. | n.a. | 19% | 3% | P :100%; V:100%; F:34%  I: 53% | 3% | n.a. |
| *Contejean, Janssen et al. 2023;* | n.a. | | 27.4% (24%) | | Upper: 18%, pneumonia 17% | | 4% | n.a. | 9% | n.a. | 12% | 1% | n.a. | n.a | 1.2m |
| *Kowalski, Lykon et al. 2025* | 82% | | 47% | | n.a. | | n.a. | n.a. | n.a. | n.a. | n.a. | n.a. | n.a. | n.a. | n.a. |
| *Choi, Byun et al* | 81% | | n.a. | | n.a. | | n.a. | n.a. | n.a. | n.a. | n.a. | n.a. | n.a. | n.a. | n.a. |
| *Park, Jang et al 2025* | 33% | | 66% | | 46% | | 11.5% | 67% | 49.2% reactivation | n.a. | 11% | 13% | I: 32.8% | 8% | 30d 13.5%; 90d: 36.5%  180d: 63.5% |
| *Lancman, Parsa et al 2024* | 100% | | 118 infections | | n.a. | | n.a. | n.a. | n.a. | n.a. | n.a. | 11% | I: 92% | 2 | n.a. |
| *Banarjee, Marsal et al 2021* | n.a. | | 6% | | n.a. | | n.a. | n.a. | n.a. | n.a. | n.a | n.a. | n.a. | n.a- | n.a. |
| *Kumar, Mateos et al 2026* | n.a. | | 31% grade 3/ 4 | | n.a. | | n.a. | n.a. | n.a. | n.a. | n.a. | n.a. | n.a. | 5 infection related deaths | n.a. |
| *Cani, Scott et al 2025* | n.a. | | 62% BCMA, 39% anti-GPRC5d | | 46% (25%) | | URTI 38%  LRTI 22% | n.a. | 60% | 47% | 37% | 3% | I: 68-72% | 4% | n.a. |
| *Al Hadidi, Szabo et al 2025* | n.a. | | 27% (18%) | | n.a. | | n.a. | n.a. | n.a. | n.a. | 41% | 0% | P:90%  V:98%  F:9.7%  B: 24%  I: 61% | none | 0.8 m |
| *Wang, Li et al 2025* | n.a.  Elranatamab (€)  Teclistamab (T) | | n.a. | | n.a. | | Pneumonia: 21% E  23.% T | n.a..COVID-19: 44.1% E 47.3% T influenz39.4% E 38.3% T | 24.5% E 18.6% T | HSV: 6.9% E 10.1% T | n.a. | n.a. | I: 12.8% E, 20.6% T | n.a. | n.a. |
| *Jelinek, Zihala et al 2026* | Anti-BCMA (B) 71%  Anti-GPRC5D (G): 33% | | B: 79% (33%)  G: 54% (34%) | | n.a. | | n.a. | n.a. | n.a. | n.a. | n.a. | n.a. | I: B 71%; G 33% | n.a. | n.a. |
| *Piron, Rodrigo et al 2025* | n.a. | | grade ≥3: 22.7% (reactive IVIG) vs 34.4% (preemptive IVIG) | | Upper RTI: 44.3% vs 37.5%; Lower RTI: 22.7% vs 34.4% | | 0% | 27.8% vs 40.6% | 14.5% vs 7.7% | HSV: 7.4% vs 0%  VZV: 3.7% vs 0% | 17.5% vs 34.4% | 1% vs 0% | P:100%  V:100% | 7.1% | First infection: 1.8 vs 5.2 m; severe infection: 3.2 vs 5.4 m |
| *Bilbao, Anson et al 2025* | n.a. | | n.a. | | n.a. | | n.a. | n.a. | n.a. | n.a. | n.a. | n.a. | n.a. | n.a. | n.a. |
| *Tan, Usmani et al 2026* | 66% | | n.a. | | n.a. | | n.a. | n.a. | n.a. | n.a. | n.a. | n.a. | 40% | 13% | n.a. |
| Idecabtagene vicleucel |  | | | | |  |  |  |  |  |  |  |  |  |  |
| *Munshi, Anderson et al. 2021;* | 41% | | 51% | | 34% | | n.a | n.a. | n.a. | n.a. | n.a. | n.a. | n.a. | n.a. | n.a. |
| *Hansen, Sidana et al. 2023;* | 34% | | n.a. | | n.a. | | n.a. | 16% | n.a. | n.a. | 20% | 1% | n.a. | n.a. | n.a. |
| *Lin, Raje et al. 2023* | n.a. | | 75.8% (22.6%) | | n.a. | | n.a. | n.a. | n.a. | n.a. | n.a. | n.a. | n.a. | n.a. | n.a. |
| *Hashmi, Hansen et al. 2024;* | 32% | | n.a. | | n.a. | | n.a. | n.a. | n.a. | n.a. | n.a. | n.a. | n.a. | n.a. | n.a. |
| *Ailawadhi, Arnulf et al. 2024;* | n.a. | | n.a. | | n.a. | | n.a. | n.a. | n.a. | n.a. | n.a. | n.a. | n.a. | 4% | n.a. |
| *Kalariya, Hildebrandt et al. 2024;* | n.a. | | n.a | | 48% | | n.a. | n.a. | n.a. | n.a. | n.a. | n.a. | n.a. | 15% | n.a. |
| *Minakata, Ishida et al. 2023 – Phase 2;* | n.a. | | 56% (11%) | | 44% | | 0% | n.a. | 11% | n.a. | 0% | 0% | n.a. | n.a. | n.a. |
| *Gaballa, Puglianini et al. 2025;* | n.a. | | 50% | | 20% | | O% | n.a. | 10% | 6-10% | 20% | 0% | n.a. | n.a. | n.a. |
| *Ferreri, Hildebrandt et al. 2023;* | n.a. | | Prior BCMA 82%  no prior BCMA 39% | | n.a. | | n.a. | n.a. | n.a. | n.a. | n.a. | n.a. | n.a. | 3% | n.a. |
| *Dima, Rashid et al. 2024;* | n.a. | | 49% | | 12% | | n.a. | n.a. | n.a. | n.a. | 22% | 5% | n.a. | n.a. | n.a. |
| *Sidana, Ahmed et al. 2025* | n.a. | | 45% | | 26% | | n.a. | n.a. | n.a. | n.a. | 23% | 2% | n.a. | 2.4% | n.a. |
| *Trando, Ghamsari et al. 2024;* | 96% | | 44%(12%) | | 32%. | | 0% | 47% | n.a. | n.a. | 8% | 0% | n.a. | n.a. | n.a. |
| *Logue, Peres et al. 2022* | n.a. | | 54% (23%) | | 42% | | n.a. | n.a. | n.a. | n.a. | 50% | n.a. | n.a. | n.a. | n.a. |
| *Kikuchi, Watanabe et al.* | n.a. | | n.a. | | n.a. | | n.a. | n.a. | 9.2% | n.a. | n.a | n.a. | n.a. | n.a. | n.a. |
| *Akhtar, Oloyede et al. 2025* | n.a. | | 343 frail patients 49.6% v.s. non frail 40.9% | | n.a- | | n.a- | n.a. | n.a. | n.a. | n.a. | n.a. | n.a. | n.a. | n.a. |
| *Cillot, Sleiman et al 2024* | n.a. | | 79% | | n.a. | | n.a. | n.a. | n.a. | n.a. | n.a. | n.a. | IVIG 88% | n.a. | n-a- |
| *Sanoyan, Seipel et al. 2023* | 85% | | 53% | | 68% | | 40% | 0% | 0% | 0% | 53% | 6% | n.a. | 3.9m | 1.8% |
| ciltacabtagene autoleucel |  | | | | |  |  |  |  |  |  |  |  |  |  |
| *Berdeja, Madduri et al. 2021;* | n.a. | | **58**%(20%) | | n.a. | | n.a. | n.a. | n.a. | n.a. | n.a. | n.a. | n.a. | n.a | n.a. |
| *Mi, Zhao et al. 2023* | 70% | | 95.4% (37.5%) | | 33.3%//35.4% | | n.a. | n.a. | n.a. | 18.8% | n.a. | n.a. | V:50% | n.a. | n.a. |
| *Sidana, Patel et al.2025* |  | | 47% (21.7%) | | 80% | | n.a. | 42.7% | n.a. | n.a. | 37.3% | 2.7% | 1.3% (1.3%) | n.a. | n.a. |
| *San-Miguel, Dhakal et al.2023* | 85% | | 76%(27%) | | 22%//14% | | 2% | n.a. | n.a. | n.a. | 30% | 10% | P:90%; V:95%; F: 88%; I:50%; B:70% | 2% | 2.5; grade 3/ 4: 3m |
| *Ri, Suzuki et al. 2022;* | n.a. | | 11.1% | | 11.1%. | | 0% | n.a. | n.a. | n.a. | 11.1% | n.a. | V/B/F:100% | O% | n.a. |
| *Htut, Dhakal et al. 2023;* | 65% | | 57.1% (20%) | | n.a. // 29% | | 0% | 0% | n.a. | n.a. | 43% | 14% | P: 100%; V:100%; F: 86%; I: 43%; B: 71% | 14% | 2 m; grade 3/ 4 2.4m |
| *Ellithi, Elsallab et al. 2025* | n.a. | | n.a. | | n.a. | | n.a. | n.a. | ROR: 3.9, 95% CI 1.6 to 9.5 | n.a. | n.a. | n.a. | n.a. | n.a. | n.a. |
| *Einsele, San-Miguel et al 2026* | 71% | | 35% (24%) | | n.a. | | n.a. | n.a. | n.a. | n.a. | n.a. | n.a. | n.a. | 4% | n.a. |
| *Dima, Loque et al 2026* | n.a. | | 49% (32%) | | n.a. | | n.a. | n.a. | n.a. | n.a. | 42% | 4.5% | I: 52% | n.a. | n.a. |
| *Hansen, Dima et al 2026* | 47% | | n.a. | | n.a. | | 33% | n.a. | n.a. | n.a. | 20% | 2% | n.a. | 11 deaths | n.a. |
| All-CAR-T cells |  | | | | |  |  |  |  |  |  |  |  |  |  |
| *Merz, Albici et al. 2025* | n.a. | | n.a. | | n.a. | | n.a. | n.a. | n.a. | n.a. | n.a. | n.a. | n.a. | 26% of deaths  50% of deaths | n.a. |
| *Hansen, Peres et al. 2025* | Ide-Cel: 67%  Cilta-Cel: 79% | | Ide-Cel 58% (28%)  Cilta-Cel 61% (34%) | | n.a. | | n.a. | 0% | n.a. | n.a. | Ide-Cel: 35%  Cilta-Cel: 42% | Ide-Cel: 7%,  Cilta-Cel 10% | P: 92-95%  V:98-99%  F: 89-91%  I:47-56% | Ide-Cel 2%  Cilta-Cel: 3% | 2.3 m (1.1-4.5)  Grade 3/ 4: 3.2 m (1.6-6.0) |
| *Xu, Wang et al.* | n.a. | | n.a. | | n.a. | | n.a. | n.a. | n.a. | n.a. | n.a. | n.a. | n.a. | n.a. | n.a. |
| *Wesson, Dima et al. 2024* | 50% | | 40%(20%) | | 15% | | 0% | 5% | n.a. | n.a. | 25% | 10% | n.a. | 5% | 2 m(1-3) |
| *Wang, Cao et al. 2022* | 45% | | 45% (27%) | | Upper resp. tract inf. <3m: 8%; >3m: 39%  Pneumonia <3m: 44%; >3m: 43% | | n.a. | n.a. | n.a. | <3m: 2%; >3m:  7 | <3m: 14%  >3m: 4% | n.a. | n.a. | n.a. | n.a. |
| *Merz, Gagelmann et al. 2025* | 79.8% | | 47.1% (17.6%) | | Upper resp. tract inf. 19.3%  Pneumonia: 6.7% | | n.a. | n.a. | 5.9% | Na. | n.a. | n.a. | n.a. | n.a. | n.a. |
| *Portuguese, Liang et al. 2025* | n.a. | | n.a. | | n.a. | | n.a. | n.a. | EMD 15%  Non EMD 8.5% | n.a.. | EMD 15%  Non EMD 2.4% . | EMD: 4%  Non EMD: 0% | n.a. | 7.4% | n.a. |
| *Hill et al, 2024* | n.a. | | n.a. | | n.a. | | n.a. | n.a. | n.a. | n.a. | n.a. | n.a. | n.a. | n.a. | n.a. |
| *Rejeski, Hansen et al 2023* | n-a- | | 39% (20%) | | 17% | | 0% | 10% | n.a. | n.a. | 25% | 2% | n.a. | 1.4% | n.a. |
| *Oliver-Caldés, González-Calle et al. 2023* | n.a. | | n.a. | | n.a. | | n.a. | n.a. | n.a. | n.a. | n.a. | n.a. | n.a. | n.a. | n.a. |
| *Xu, Wang et al. 2024* | n.a. | | n.a. | | n.a. | | n.a. | n.a. | n.a. | n.a. | n.a. | n.a. | n.a. | n.a. | n.a. |
| *Mailankody, Matous et al. 2023* | n.a. | | 53.5% (23.3%) | | n.a. | | n.a. | n.a. | n.a. | n.a. | n.a. | n.a. | n.a. | n.a. | n.a. |
| *Ravi, Richard et al. 2025* | n.a. | | 32% (5%) | | n.a. | | n.a. | n.a. | n.a. | n.a. | n.a. | n.a. | n.a. | n.a. | n.a. |
| *Zhao, Wang et al. 2022* | n.a. | | n.a. | | n.a. | | n.a. | n.a. | n.a. | 5.4% | n.a. | n.a. | n.a. | 2.7% | n.a. |
| *Little, Tandon et al. 2023* | 70% | | 37% | | n.a. | | 41% | n.a. | n.a. | n.a. | n.a. | n.a. | IVIG:63% | n.a. | n.a. |
| *Richardson, Schütte et al. 2025* | n.a. | | 71% | | 52.6% | | n.a. | 63% | 7% | 1.8% | 35% | 5% | n.a. | 7% | 38 days |
| *Xia, Sun et al 2025* | 27% | | 51% (22%) | | n.a. | | n.a. | 22%. | n.a. | n.a. | 19%. | 11% | n.a. | 0% | n.a. |
| *Frenking, Zhou et al 2024* | n.a. | | Late grade 3/ 4 infections 13% | | n.a. | | n.a. | n.a. | n.a. | n.a. | n.a. | n.a. | n.a. | n.a. | n.a. |
| *Reyes, Huang et al 2023* | 82% | | 36% v.s. 52% | | n.a. | | n.a. | n.a. | n.a. | n.a. | n.a. | n.a. | n.a. | n.a. | n.a. |
| *Wang, Li et al 2021* | 53% | | 57.5% | | n.a. | | n.a. | n.a. | n.a. | n.a. | n.a. | n.a. | n.a. | n.a. | n.a. |
| *Thiruvengadam, Sheng et al. 2022* | 76% | | 53% | | 40% | | n.a. | 53% | n.a. | n.a. | n.a. | 6% | n.a. | n.a. | n.a. |
| *Josyula, Pont et al 2022* | 88% | | 53% | | n.a. | | n.a. | 61% | n.a. | n.a. | 30% | 9% | n.a. | n.a. | 46 days |
| *Little, Pena et al 2025* | n.a. | | n.a. | | n.a. | | n.a. | n.a. | n.a. | n.a. | n.a. | 1.7% | F: 12% | 0.9% | 0.2-3.4 m |
| *Yan, Du et al 2026* | n.a. | | 30.6% (19.4%) | | n.a. | | n.a. | n.a. | n.a. | n.a. | n.a. | n.a. | n.a. | n.a. | n.a. |
| *Cohen, Lebel et al 2026* | n.a. | | n.a. | | n.a. | | n.a. | n.a. | 20% | n.a. | n.a. | n.a. | V: 100% | n.a. | n.a. |
| *Habib, Ahmed et al 2026* | n.a. | | Grade 3/ 4: 44% v.s 20% | | n.a. | | n.a. | n.a. | n.a. | n.a. | n.a. | n.a. | n.a. | n.a. | n.a. |
| *Liu, Zhang et al 2026* | 80% | | Sever 29.9% | | n.a. | | n.a. | n.a. | n.a. | n.a. | n.a. | n.a. | n.a. | n.a. | n.a. |
| CAR-T cells and bispecific antibodies (BsAb) | | | |  | |  |  |  |  |  |  |  |  |  |  |
| *Nath, Shekarkhand et al. 2024* | n.a. | | All: 83.5%  CAR-T: 125% (27%)  BsAb: 180% (40%) | | CAR-T: 54% | | CART: 0%  BsAb: 1% | CAR-T: 46%  BsAb: 43% | CAR-T: 0.9%  BsAb: 2% | CAR-T:  0.9%  BsAb: 3% | CAR-T 46%  BsAb: 54% | CAR-T: 4%  BsAb: 3% | n.a. | n.a. | n.a. |
| *Howard, Concepcion et al. 2024* | n.a. | | E.D visit:  CAR-T: 20%  BSAB: 24% | | E.D. visit:  CAR-T: 12%  BSAB: 16% | | n.a. | n.a. | n.a. | n.a. | n.a. | n.a. | n.a. | n.a. | n.a. |
| *Hammons, Haider et al. 2024* | n.a. | | Grade 3:  CAR-T: 50%  BSAB: 44% | | n.a. | | n.a. | n.a. | n.a. | n.a. | n.a. | n.a. | n.a. | n.a. | n.a. |
| *Lesokhin, Nath et al 2024* | CAR-T: 60%  BsAb: 49% | | CAR-T: 60%  BsAb: 73% | | n.a. | | CAR-T:0%  BsAb:42% | CAR-T: 48%  BsAb 42% | CAR-T: 1%  BsAb: 1% | CAR-T: 0%  BsAb:2% | CAR-T;49% BsAb 53% | CAR-T: 4% BsAb: 3% | n.a | CAR-T: 0% BsAb: 7% | n.a. |
| *Mersi, Burow et al 2026* | n.a. | | 76-85% (8-19%) | | 31-39% | | n.a. | COVID-19: 19% | n.a. | n.a. | n.a. | n.a. | n.a. | n.a. | n.a. |

| Supplement Table 3.b: study characteristics and outcomes | | | | |  |  |  |  |  |  |  |
| --- | --- | --- | --- | --- | --- | --- | --- | --- | --- | --- | --- |
| Study | **Prior**  **line** | **Patient**  **count** | **Follow up**  **months (m)** | **ORR**  **(CR, MRD)** | | **DOR**  **m** | **PFS**  **m** | **OS**  **m** | **CRS**  **Any grade (3/4)** | **ICANS**  **Any grade (3/4)** | **Neutropenia**  **Any grade (3/4)** |
| Teclistamab |  | | | |  |  |  |  |  |  |  |
| *Moreau et al N Engl J med 2022* | 5 | 165 | 14.1 | 63% (39.4%; 26.4%) | | 18.4 | 11.3 | 18.3 | 72.1% (0.6%) | 14.5%  (0%) | 70.9% (64.2%) |
| *Usmani, Garfall et al. 2021* | 6 | 157 | 6.1 | 65% | | n.a. | n.a. | n.a. | 57% (0%) | n.a. | 65% (40%) |
| *Nooka, Rodriguez et al. 2024* | 5 | 165 | 22.8 | n.a. | | n.a. | n.a. | n.a. | n.a. | n.a. | 71.5% (65.5%) |
| *Dima, Davis et al. 2024* | 6 | 106 | 3.8 | 66% (29%) | | n.a. | 5.4 | n.a. | 64% (1%) | 14% (3%) | 47% (21%) |
| *Frerichs, Verkleij et al. 2024* | n.a. | 52 | 5 (1-31) | ≥VGPR (61.1%) | | n.a. | n.a. | n.a. | n.a. | n.a. | n.a. |
| *Riedhammer, Bassermann et al. 2024* | 6 (3-14) | 123 | 5.5. | 59.3% (22%) | | n.a. | 8.7 . | n.a. | 58.5% (1.6%) | 7.3% (0.8%) | 55.3% (38%) |
| *Touzeau, Krishnan et al. 2024* | All prior anti-BCMA therapy | 40 | 28 (0.7-31.11) | 52.5% (30%) | | 14.8 | 4.5 | 15.5 | n.a. | n.a. | n.a. |
| *Kawasaki, Steele et al. 2024* | 5 | 28 | n.a. | n.a. | | n.a. | n.a. | n.a. | 50% | 17% | 22% |
| *Tan, Asoori et al. 2025* | 6 | 210 | 5.3 m | 67% (21%) | | 11 | 6m:53% | 6m 73% | 54% | 7.3% | Grade 3: 39.5% |
| *Ariane, Stéphanie et al. 2025* | n.a. | 35 | 6 m | n.a. | | n.a. | n.a. | n.a. | n.a. | n.a. | n.a. |
| *Perrot, Hulin et al. 2024* | 4 | 303 | 11.9 | 68.8% (≥VGPR 61.4%) | | n.a. | 17 | 17 | n.a. | n.a. | n.a. |
| *Ishida, Kuroda et al. 2025* | 5 | 14 | .314 | 76.9% (65.4%) | | n.a. | n.a. | n.a. | 80.8% | 7.7% | 73.1% |
| *Mohan, Monge et al. 2024* | 6 | 110 | 3.5 (0.4-10.9) | 62% (≥VGPR 51%) | | n.a. | 6 m 52% | 6 m 80% | 56% | 11% | n.a. |
| *Coi, Zhongjun et al 2024* | 5 | 26 | 15 | 76.9% (57.5%) | | n.r.. | n.r. | n.r. | 96.2% | 0% | 96.2% |
| *Razzo, Midha et al 2026* | 6 | 509 | 10.1 | 53% (25%) | | n.a. | 5.8 | 61% at 12m | 54% (1.4%) | 11% (2.2%) | Grade ≥3: 17% within 30 d; 33% between day 30–180 |
| *Bros, Harel al 2025* | 3 | 35 | 12 | n.a. | | n.a. | n.a. | n.a. | n.a. | n.a. | n.a. |
| *Snyder, Atrash et al 2025* | 5 | 88 | 12 | CR ≥44% continuous, 69% fixed; MRD NR | | n.a. | 16 continuous; 13 fixed | n.a. | 54% continuous; 57% fixed; grade 3/4: 0% fixed | 5.6% continuous; 6.3% fixed; grade 3/4: 0% fixed | n.a. |
| *Stork, Radocha et al 2025* | 5 | 73 | 4.9 | 58.8% ORR | | n.r. | 9.4 | 15.4 | 49% (0%) | 2.7% (0%) | 52.1% (28.8%) |
| *Yi, Lee et al 2025* | 6 | 42 | 16.4 | 66.7% (40.5%) | | n.r. | 14.1 | n.r. | 47.6% (2.4%) | 0% | 73.8% (69%) |
| *Smits, Groen et al 2026* | 5 | 80 | 21 | n.a. | | n.a. | 18.1 | 43.5 | n.a. | n.a. | n.a. |
| *Ssentongo, Guare et al 2026* | 5 | 19 | 6.4 | n.a. | | n.a. | n.a. | n.a. | 63.2% | 36.8% | n.a. |
| *Cheema, Shrestha et al 2026* | n.a. | 177 | 24.2 | n.a. | | n.a. | n.a. | 24.1 | n.a. | n.a. | n.a. |
| *Costa, Bahlis et al 2025* | 1 | 291 | 34.5 | 89% (81.8%; MRD: 58.4%) | | 36m: 88.5% | 36m: 83.4% | 36m: 83.3% | 60.1% (0%) | 1.1% (0.4%) | 75.6% (75.6%) |
| Elranatamab |  | | | |  |  |  |  |  |  |  |
| *Lesokhin, Tomasson et al. 2023* | 5 | 123 | 14.7 (0.2-25.1) | 61% (35%, 23.6%) | | n.r | n.r. | n.r. | 57.7% (0%) | 3.4% (0%) | 48.8% (37.4%) |
| *Bahlis, Costello et al. 2023* | 5 | 55 | 12 (0.3-32.3 | 63.6% (27.3%, 23.6%) | | 17.1 | 11.8 | 21.2 | 87.3% (0%) | 7.3% (0%) | 74.5% (70.9%) |
| *Tomasson, Iida et al. 2024* | 5 | 123 | 23 | 61%72 (37.4%,) | | 28.4 | 17.2 | 24.6 | n.a. | n.a. | n.a. |
| *Iida, Ito et al. 2024* | 7.5 | n.a. | n.a. | 50% | | n.a. | n.a. | n.a. | 100% (0%) | n.a. | n.a. |
| *Malard, Bobin et al. 2024* | 5 | 101 | 15.5(3.4-18.8) | 51.5% (≥VGPR 42%) | | 1 year 48% | 1 y 34% | 1 y 42% | 45% (0%) | 3% (0%) | n.a. |
| *Soltantabar, Hibma et al 2026* | n.a. | 324 | n.a. | n.a. | | n.a. | n.a. | n.a. | n.a. | n.a. | 48.1% grade ≥3 |
| *Portuguese, Davis et al 2026* | 6 | 130 | 7.5 | 65% (36%) | | 12.2 | 4.3 | 14.6 | 40% (2.3%) | 17% (7.7%) | n.a. |
| Linvoseltamab |  | | | |  |  |  |  |  |  |  |
| Bumma, Richter et al. 2024 | 28% pentarefractory | 117 with 200mg | 14.3 m | 71% (50%) | | 29.4 | n.r. | n.r. | 35% (0.9%) | 7.7% (2.6%) | 42.8%( (41.9%) |
| Lee, Zonder et al 2025 | 5 | 117 with 200mg | 21.3 | 71% (52%) | | 29.4 | n.r. | 31.4 | 46% (1%) | 7.7% (2.6%) | 44% (43%) |
| AMG 420 |  | | | |  |  |  |  |  |  |  |
| *Topp, Duell et al. 2020* | 5 | 42 | n.a. | 31% | | n.a. | n.a. | n.a. | 38% | n.a. | n.a. |
| *Pavurutamab* |  |  |  |  | |  |  |  |  |  |  |
| *Lee, Plattel et al 2026* | 6 | 172 | 17.2 | 46.5% (29.1%) | | 36.6 | 5.5 | 18.1 | 74.4% (8.1%) | 0.6% (0%) | 47.1% (41.9%) |
| Alnuctamab |  |  |  |  | |  |  |  |  |  |  |
| *Bar, Martin et al 2026* | 4 | 95 | 11.8 | 58.9% (35.8%) | | n.a. | 11.5 | n.a. | 57.9% (0%) | 2.1% (0%) | 53.7% (43.2%) |
| Talquetamab |  | | | |  |  |  |  |  |  |  |
| *Chari, Minnema et al. 2022* | 6 | 232 | 11.7 | n.a. | | 10.2 and 7.8 | n.a. | n.a. | 77-80% (0-3%) | 10% | 36-67% (32-60%) |
| *Schinke, Dhakal et al. 2024* | n.a. | n.a. | n.a. | n.a. | | n.a. | n.a. | n.a. | n.a. | n.a. | n.a. |
| *Chari, Touzeau et al. 2025* | n.a. | n.a. | 25.6 (8.5-25.9) | 74% -67% (33-41%) | | 9.5-16.5 | 7.5-11.2 | 1year 74-77% | 75-79% (1-2%) | 29-44% (22%) | 4-11% |
| *Frenking, Riedhammer et al. 2025* | 6 | 138 | 8 | 56.3% (CR:25%) | | 9.6 | 5.2 | 10.3 | 70.3% (0.7%) | 3.6% (0.7%) | 10.9% (2.9%) |
| *Iida, Sunami et al 2025* | 5 | 15 | 9 | 60% (CR 13.3%) | | 7.6 | n.a. | n.a. | 46.7% (0%) | 0% | 60% |
| *Dhakal, Akhtar et al 2025* | 5 | 134 | n.a. | 71% prior CAR-T | | n.a. | n.a. | n.a. | No grade ≥3 CRS reported | 2% | n.a. |
| *Schinke, Rodriguez-Otero et al 2025* | 3 | 399 | 12.7-18.8 | 74% | | n.a. | n.a. | n.a. | n.a. | n.a. | 55% (53%) |
| *Cani, Szabo et al 2025* | 6 | 114 | 6.7 | 73% (26%) | | n.a. | 10 | n.a. | 54% (7.3%) | 9.8% (5.9%) | n.a. |
| *Janakiram, Tan et al 2026* | 6 | 151 | 12.7 | 67.6% | | n.a. | 7 | n.a. | n.a. | 8.1% (0.7%) | 36% (36%) |
| Cevostamab |  |  |  |  | |  |  |  |  |  |  |
| *Trudel, Cohen et al. 2021* | 6 | 167 | 6.1 | 160mg 54.4%  90 mg 36.7% | | 15.6 | n.r. | n.r. | 80.7% (1.2%) | 14.3% | 18.1% (16.3%) |
| *Lesokhin, Richter et al 2024* | 6 | 256 | 5.8- 3.8m | n.a. | | n.a. | n.a. | n.s. | n.a. | n.a. | n.a. |
| All bispecific antibodies |  | | | |  |  |  |  |  |  |  |
| *Jourdes, Cellerin et al. 2024* | n.a. | 229 | n.a. | n.a.. | | n.a. | n.a. | n.a. | n.a. | n.a. | n.a. |
| *Li, Zhang et al. 2025* | n.a. | 9 | n.a. | n.a. | | n.a. | n.a. | n.a. | n.a. | n.a. | n.a. |
| *Cohen, Magen et al. 2025* | n.a. | 94 | 20.3 | 78% | | 86% 18 m | n.a. | n.a. | n.a. | n.a. | 96% |
| *Hammons, Szabo et al. 2024* | n.a. | 66 | n.a. | n.a. | | n.a. | n.a. | n.a. | n.a. | n.a. | n.a. |
| *Mohan, Szabo et al. 2025* | 5 | 225 | IVIG: 9 m  No IVIG: 11 m | n.a. | | b.a. | IVIG 15 m  No IVIG8 | IVIG 44 m  No IVIG 16 | 54.55% | 3.3-9% | 3.8-2,2% |
| *Lancman, Parsa et al. 2023* | 7 | 37 | 18.6 | 70% (VGPR or better 68%, MRD-sCR 41%) | | n.a. | n.a. | n.a. | 63% | n.a. | 73% (62%) |
| *Uttervall, Tätting et al. 2024* | 7 | 58 | 15.1 | 69& (48%) | | n.a. | 15 m: 60% | 15 m: 69% | 65% | 1.7% | 52% (43%) |
| *Sim, Longhitano et al. 2023* | 6 | 39 | 5.1 | n.a. | | n.a. | n.a. | n.a. | n.a. | n.a. | n.a. |
| *Contejean, Janssen et al. 2023* | n.a. | 692 safety reports | n.a. | n.a. | | n.a. | n.a. | n.a. | n.a | n.a. | n.a. |
| *Kowalski, Lykon et al. 2025* | n.a. | 72 | 5 | 66% (38%) | | n.r. | n.r. | n.r. | 14% (0%) | 8% (2.8%) | n.a. |
| *Choi, Buyn et al. 2024* | n.a. | 71 | n.a. | n.a. | | 19.2m | n.a. | n.a. | 52% | 4% | n.a. |
| *Park, Jang et al 2025* | 4 | 61 | n.a. | n.a. | | n.a. |  | n.a. | 60.7% | 0% | 60.7% |
| *Lansman, Parsa et al 2024* | 7 | 37 | n.a. | 70% (VGPR 68%, 41%) | | n.a. | 17m | 26.3m | 64% | 0% | 73% |
| *Banerjee, Marsal et al 2021* | n.a. | 50 | 15.3 | 74% | | n.a. | n.a. | n.a. | 90% | 24% | n.a. |
| *Kumar, Mateos et al 2026* | n.a. | 90 | 12.7 | 79% | | 64% at 12 m | 61% at 12m | 75% at 12m | 78% | n.a. | n.a. |
| *Cani, Scott et al* | 5 | 158 | 6.1 anti-BCMA; 4.5 anti-GPRC5D | n.a. | | n.a. | n.a. | n.a. | 28% anti-BCMA, 21% anti-GPRC5d | 11% | 63% anti-BCMA; 58% anti-GPRC5d |
| *Wang, Li et al 2026* | 5 | 188 Elranatamab €/ 188 Teclistamab (T) | Up to 36 | n.a. | | n.a. | n.a. | 3-y-OS: 58.8% E 59.7% T | 45.2% E . 27.1% T | 18.6% E  13.3% T | Grade ≥3:  74.5% E  84.6% T |
| *Jelinek, Zihala et al 2026* | 3 | Anti-BCMA (B) 86;  Anti-GPRC5D (G): 92 | 24m | n.a. | | n.a. | n.a. | n.a. | n.a. | n.a. | n.a. |
| *Piron, Rodrigo et al 2025* | 5 | 66 | 9 | n.a. | | n.a. | n.a | n.a. | 56.1% (0%) | 0% | n.a. |
| *Bilbao, Anson et al 2025* | 4 | 85 | n.a. | n.a. | | n.a. | n.a. | n.a. | n.a. | n.a. | n.a. |
| *Tan, Usmani et al 2026* | 5 | 441 | 17.1 | 66% | | n.a. | 12m 44.9% | n.a. | n.a. | n.a. | n.a. |
| Idecabtagene vicleucel |  | | | |  |  |  |  |  |  |  |
| *Munshi, Anderson et al. 2021* | 6 | 128 | 13,3 | 73% (33%, 26%) | | n.a. | 8.8 | n.a. | 85% (5%) | 18% (3%) | 91% |
| *Hansen, Sidana et al. 2023* | 3 | 159 | 6.1 | 84%(42%) | | n.a. | 8.5 | 12.5 | 82% (3%) | 18% (6%) | 91%(76.7%) |
| *Lin, Raje et al. 2023* | 6 | 62 | 18.1 | 75.8% (38.7%) | | 10.3 | 8.8 | 34.2 | 75.8% (6.5%) | 37.1% (1.6%) | 88.7%. |
| *Hashmi, Hansen et al. 2024* | 7 | 211 | 9.9 | n.r. | | n.r. | n.a. | n.a. | 82% (2.8%) | 13% (6.5%) | 66% (37%) |
| *Ailawadhi, Arnulf et al. 2024* | 2-4 | 386 | 30.9 | 71% (39%) | | 16.6 | 13.3 | n.r. | 88% (5%) | 15% (3%) | 79% |
| *Kalariya, Hildebrandt et al. 2024* | 4 | 75 | 14.2 | 86.7% (56%) | | n.a. | 9.1 | 26.5 | 88% (1%) | 25.3% (4%) | n.a. |
| *Minakata, Ishida et al. 2023 – Phase 2* | 4 | 10 | 12.9 | 89% (56%) | | n.a. | 12m: 75% | 12m: 78% | 100% (0%) | 33% (0%) | 100% (100%) |
| *Gaballa, Puglianini et al. 2025* | 6 | 10 | 13.6 | 80% | | n.a. | 13.3 | 6.3 | 80% (0%) | 20% (10%) | 30% |
| *Ferreri, Hildebrandt et al. 2023* | BCMA prior treatment:  9  no prior BCMA: 6 | 203  Prior BCMA: 50  no prior BCMA: 153 | 4.5 | Prior BCMA: 74% (29%)  no prior BCMA 88% (48&) | | Prior BCMA 7.4 m  no prior BCMA 7.4 m | Prior BCMA 3.2 m  No prior BCMA 9 m | 6 m: 72-87% | Prior BCMA: 80% (2%)  no prior BCMA 86% (3.3%) | Prior BCMA 17% (8.5%)  no prior BCMA 22% (6.9%) | Prioir BCMA 88% (76%)  No prior BCMA 93% (78%) |
| *Dima, Rashid et al. 2024* | 6 | 120 | 13.3 | 73% (33%) | | n.a. | 8.8 | 19.4 | 81% (3.4%) | 28% (3.3%) | 89% |
| *Sidana, Ahmed et al. 2025* | 7 | 821 | 11.6 | 73% (25%) | | n.a. | 8.8 | n.a. | 80% (3.3%) | 28%(5%) | 6% |
| *Trando, Ghamsari et al. 2024* | 6 | 25 | 13 | 84%(56%) | | n.a. | 13.9 | n.r. | 92% (4%) | 12%(4%) | 96% |
| *Logue, Peres et al. 2022* | 4 | 52 | n.a. | 84% (36%) | | n.a. | n.a. | n.a. | 85% (6%) | 19% (8%) | 35% (11%) |
| *Kikuchi, Watanabe et al* | n.a. | 53 | n.a. | n.a.% | | n.a. | n.a. | n.a. | n.a. | n.a. | n.a. |
| *Akhtar, Oloyede et al. 2025* | n.a. | 821  <70y: 570  >70y: 251 | 11.6 | <70y: 36.9%  >70y: 26.6% | | n.a. | 6-m:  <70y 68.3%  >70y:60.4% | 6-m:  <70y: 86%  >70y: 83% | n.a. | n.a. | n.a. |
| *Caillot, Seilman et al 2024* | 4 | 24 | 92% | n.a. | | n.a. | n.a. | n.a. | 96% | 12.5% | 58% |
| *Sanoyan, Seipel et al. 2023* | n.a. | 16 | 5.7m | 69% | | n.a. | n.a. | n.a. | 94% (0%) | 6% (0%) | 69% |
| Ciltacabtagene autoleucel, |  | | | |  |  |  |  |  |  |  |
| *Berdeja, Madduri et al. 2021* | 6 | 97 | 12.4 | **97%** (67%, MRD in 57 pts 93%) | | n.r. | 1year 77% | 1 year 89% | **95% (4%)** | **21% (9%)** | 95.9% (94.8%) |
| *Mi, Zhao et al. 2023* |  | 48 | 18 | 89.6% (77.1%, MRD in 40 pts 97.5%) | | n.r. | 18 m 66.8% | 18 months 78.7% | 97.9% (35.4%) | 2.1% | 97.9% |
| *Sidana, Patel et al.2025* | 6 | 255 | 13 | 84-95% (70-76%, MRD in 98 pts 94.9) | | n.r. | 12 m 68% | 12 m 82% | 75% (5%) | 14%(4%) |  |
| *San-Miguel, Dhakal et al.2023* | 2 | 208 | 15.9 | 84.6% (73.1%) | | n.r | n.r. | n.r. | 76% (1%) | 5%(1%) | 94% (91%) |
| *Ri, Suzuki et al. 2022* | 5 | 9 | 8.5 | 100% (25%; MRD in 6 pts 100%) | | n.r. | n.r. | 8.5 m 100% | 88.9% (0%) | 0% (0%) | 88.9% (all grade3 /4) |
| *Htut, Dhakal et al. 2023* | 9 | 7 | 27.7 | 85% (71.4%) | | n.r. | n.r. | n.r. | 85.7% | 14.3%(0%) | 100% |
| *Ellithi, Elsallab et al. 2025* | n.a. | 1496 BCMA-CAR-T | n.a. | n.a. | | n.a. | n.a. | n.a. | n.a. | n.a. | n.a. |
| *Einsele, San-Miguel et al* | 1-3 | 419 | 33.6 | 84.6% (CR 73.1%, MRD neg. 60.6%) | | n.r. | n.r. | n.r. | 76% (1%) | 4.5% (1%) | Grad 3 /4 97.9% |
| *Dima, Loque et al 2026* | 4 | 105 | 6 | 92% (63%) | | n.a. | n.a. | n.a. | n.a. | n.a. | n.a. |
| *Hansen, Dima et al 2026* | 7 | 595 | 12 | 87% (35%) | | n.a. | 12m 73% | 12m 85% | 80% (4%) | 22% (4%) | At day 30 15% |
| All Car-T cells |  | | | |  |  |  |  |  |  |  |
| *Merz, Albici et al. 2025* | Ide-Cel: 5  CiltaCel: 6 | 162  42 | 12.5  8.9 | 79% (26%)  93% (48%) | | n.a.  n.a. | 10 m 47%  10m 82% | 10m 77%  10m 90% | 85% (4%)  81% (10%) | 19%(2%)  19% (7%) | n.a. |
| *Hansen, Peres et al. 2025* | 3 | Ide-Cel: 318  Cilta-cel: 269 | Ide-Cel: 13.1  CiltaCel: 12.4 | Ide-cel: 72% (28%)  Cilta-cel: 84% (60%) | | Ide-Cel: 9  Cilta-Cel: 15.9 | Ide-Cel 7.4 Cilta-Cel: 13.8 | Ide-Cel 14.4  Cilta-Cel n.r. | Ide-Cel:88% (2%)  Cilta-Cel: 95% (5%) | Ide-Cel 91% (84%)  Cilta-Cel: 21% (5%) | Ide-Cel 91% (84%)  Cilta-Cel 94% (88%) |
| *Xu, Wang et al* | n.a. | 74 | 65.4 | n.a. (CR 73%) | | n.a. | 5years 21% | 5 years 49.1% |  |  |  |
| *Wesson, Dima et al. 2024* | n.a. | 126 | 12 | 85%(40%) | | 11 | 9 | 18 | 70% (10%) | 15% (5%) | 60% |
| *Wang, Cao et al. 2022* | 4 | 69 | 21.3m | 92%(32%) | | 20.3 m | 18.3m | n.r. | 95% (10%) | 11% (3%) | 98% (94%) |
| *Merz, Gagelmann et al. 2025* | n.a. | Ide-Cel  266  Cilta-Cel  77 | n.a. | Ide  82%  Cila-Cel  94% | | n.a. | 10m:  47%  Cilta-Cel  76% | 10m:  7%  Cilt-Cel:  5% | Ide-Cel  81%  CiltaCel  85% | Ide-Cel  15%  CiltaCel  25% | n.a. |
| *Portuguese, Liang et al. 2025* | EMD 6  Non EMD 5 | Ide.Cel 32  Cilta-Cel 76  EMD 26 | 25.8 | EMD 88% (20%)  Non EMD 92% (59%) | | EMD 10.7  Non EMD 23.4 | EMD 7.6  Non EMD 24.6 | EMD 20  Non EMD n.r. | n.a. | EMD G1 38%  G3 19%  Non EMD G1 17%  G3 1.2% | EMD 96%  Non EMD  78% |
| *Hill, Martens et al 2024* | n.a. | 65 CAR-T | 7.9m | . n.a. | | n.a. | n.a. | n.a. | n.a. | n.a. | n.a. |
| *Rejeski, et al 2023* | 6 | 113 | 7.9m | 80%(39%) | | n.a. | 1yPFS: 47% | 1yOS:71% | 72% | 14%(7%) | 75%(27%) |
| *Oliver-Caldés, González-Calle et al. 2023* | >3 | 74 | 65.4 m | 87.8% (CR 73%) | | 23.3m | 18m | n.r. | 91.9% (9.5%) | 0.7% | 100% (60.8%) |
| *Xu, Wang et al. 2024* | >2 | 60 | n.a. | 100% (67%) | | n.a. | n.a. | n.a. | n.a. | n.a. | n.a. |
| *Mailankody, Matous et al. 2023* | n.a. | 43 | n.a. | 55.8% (6%) | | 8.3 m | n.a. | n.a. | 55.8% (2.3%) | 14% (0%) | n.a. |
| *Ravi, Richard et al. 2025* | 5 | 65 | 10.2 m | 95% (46%) | | n.a. | 12.3m | n.a. | 82% (2%) | 8% (2%) | 76% |
| *Zhao, Wang et al. 2022* | n.a. | 74 | 47.8 m | 87.8% (73%) | | 23.3 m | 18 m | n.r. | 91.9% (9.5%) | 1.4% | n.a. |
| *Little, Tandon et al. 2023* | 6 | 99 | n.a. | n.a. | | n.a. | n.a. | n.a. | 86% | 15% | 45% |
| *Richardson, Schütte et al. 2025* | 6.5 80 | 6.6m | n.a. | n.a. | | n.a. | n.a. | n.a. | 90% | 13% | 44% |
| *Xia, Sun et al 2025* | 6 | 37 | 6m | 84% (35%; 54%) | | 4.5m | n.a. | n.a. | 70% | 3% | 100% |
| *Frenking, Zhou et al 2024* | 5 | 129 | 9.6 | 78% | | 8.6m. | n.a. | n.r. | 84% (1%) | 9% (3%) | Grade 3/ 4  ≤30 92%;  >30: 39% |
| *Reyes, Huang et al 2023* | 6 | 83 | 6m | 82% | | 13.1m | n.r. | n.r. | 77% (0%) | 9% (0%) | n.a. |
| *Han, Zhou et al 2020* | n.a. | 40 | 7m | n.a. | | n.a. | n.a. | n.a. | n.a. | n.a. | n.a. |
| *Wang, Li et al 2020* | n.a. | n.a. | n.a. | n.a. | | n.a. | n.a. | n.a. | n.a. | n.a. | n.a. |
| *Thiruvengadam, Sheng et al. 2022* | 6 | 55 | 6 m | n.s. | | 12m | 24.8m | n.a. | 87% | 14% | 98% |
| *Josyula, Pont et al 2022* | 8 | 32 | 180 days | n.a. | | n.a. | n.a. | n.a. | 72% | 16% | 88% |
| *Little, Pena et al 2025* | 6 | 234 | 12 | n.a. | | n.a. | n.a. | n.a. | 84.6% (4.3%) | 15.3% (4.7%) | n.a. |
| *Yan, Du et al 2026* | 0 | 36 | 15.8 | CR 94.4% | | n.a. | n.a. | n.a. | 52.8% (0%) | 5.6% (0%) | 88.9% (88.9%) |
| *Cohen, Lebel et al 2026* | 5 | 75 | 12 | 92% (55%) | | n.a. | 11.6 | n.a. | n.a. | n.a. | n.a. |
| *Habib, Ahmed et al* | 6 | 223 | 27.9 | n.a. | | n.a. | 21.9 vs. 15 | n.r. vs. 27.9 | 82% | 60% vs. 19% | 84% vs. 76% |
| *Liu, Zhang et al 2026* | n.a. | 147 | 12 | n.a. | | n.a. | n.a. | n.a. | n.a. | n.a. | n.a. |
| CAR-T cells and bispecific antibodies |  | | | |  |  |  |  |  |  |  |
| *Nath, Shekarkhand et al. 2024* | 7 with CAT-T  6 with BSAB | CAR-T: 92  BSAB: 55  ADC 109 | CAT-T: 5.7 m  BSAB: 4.3m | n.a. | | n.a. | n.a. | n.a. | n.a. | n.a | n.a. |
| *Howard, Concepcion et al. 2024* | Penta refractory:CAR-T 17%  BSAB: 43% | CAR-T: 23  BSAB: 23 | n.a. | n.a. | | n.a. | n.a. | n.a. | n.a. | n.a. | n.a. |
| *Hammons, Haider et al. 2024* | n.a. | CAR-T:24  BSAB: 9 | n-a- | CAR-T: 92%  BSAB: 44% | | n.a. | n.a. | n.a- | CAR-T: 83%  BSAB: 78% | CAR-T: 12.5% | n.a. |
| *Lesokhin, Nath et al. 2024* | 6 | 16 | n.a. | CR 62.5% | | n.a. | n.a. | n.a. | n.a. | n.a. | n.a. |
| *Mersi, Burow et al 2026* | 4 | 137 | n.a. | n.a. | | n.a. | n.a. | n.a. | n.a. | n.a. | n.a. |

**Supplementary Table 3 a/b.** shows the results of all included studies. It provides patient characteristics such as median prior treatment, number of patients, and median follow-up in months. Response data are also presented, including the overall response rate (ORR), progression-free survival (PFS), overall survival (OS), and duration of response (DOR), all reported in months. Side effects are reported as percentages for cytokine release syndrome (CRS), immune effector cell neurotoxicity syndrome (ICANS), and infections. Where specified, gradings are also shown. More detailed information on infections is provided, including the frequency of bacterial, Pneumocystis jiroveci (PJP), fungal, and viral infections. Infections caused by herpes zoster (HZV), herpes simplex (HSV), cytomegalovirus (CMV), and parvovirus B19 are also considered. Additionally, more detailed information on prophylactic measures has been reviewed (immunoglobulin administration = IVIG, fungal prophylaxis = F, viral prophylaxis = V, and bacterial prophylaxis = B). The frequency of infection-related deaths and the median time to infection onset in months are also provided. Additional abbreviations: **Additional abbreviations:** URTI, upper respiratory tract infection; LRTI, lower respiratory tract infection; BCMA, B-cell maturation antigen; GPRC5D, G protein-coupled receptor family C group 5 member D; CAR-T, chimeric antigen receptor T-cell therapy; BsAb, bispecific antibody; ED, emergency department; NR, not reported; NA, not available. Prophylaxis abbreviations: P, PJP prophylaxis; V, antiviral prophylaxis; F, antifungal prophylaxis; B, antibacterial prophylaxis; I, immunoglobulin replacement.
