# Supplementary material for: Infectious toxicities associated with bispecific antibodies and CAR-T Cells in multiple myeloma: a systematic review
Source: Ann Hematol. 2026 Jun 18;105(9):397. doi: 10.1007/s00277-026-07140-8 (PMC13521976; doi:10.1007/s00277-026-07140-8)
Supplement: Supplementary file 4 [file 277_2026_7140_MOESM4_ESM.docx]

| Supplementary Table 4: Summary Table  Teclistmab | | |
| --- | --- | --- |
| Study | **primary // secondary outcome** | **summary** |
| *Moreau et al N Engl J med 2022* | ORR// VGPR, CR, MRD, DOR, PFS, OS, TTR, Safety | In the phase 1/2 study, the objective overall response rate (ORR) of weekly teclistamab (1.5 mg/kg) was analysed in 165 patients with relapsed/refractory multiple myeloma (median of five previous therapies), with a median follow-up of 14.1 months. In addition to an ORR of 63%, CRS was observed in 72% of patients, and neutropenia in 70.9%. Infections were common (76.4%, with grade 3 occurring in 44.8%), as was hypogammaglobulinaemia (74.5%), yet only 65 patients received immunoglobulins. Twelve cases of SARS-CoV-2 infection and one case of streptococcal pneumonia led to death |
| *Usmani, Garfall et al. 2021* | Tolerability, ORR // Depth of response, DOR, PFS, OS, Safety | In the dose-finding study, different dose levels of teclistamab were administered intravenously or subcutaneously. The aim was to determine the optimal dose. The safety and efficacy (ORR: 65%) were analysed in 157 patients. The most common adverse events were CRS (70%), neutropenia (65%) and infections (60%, grade 3: 22%). Upper respiratory tract infections (6.3%) and pneumonia (7.6%) were also reported. Most patients received antiviral prophylaxis (83%), but only 11% received immunoglobulins |
| *Nooka, Rodriguez et al. 2024* | ORR// Safety | This Majestec 1 study analysis focused on prevention and management of infections. infections occurred in 80% of patients (55.2% were grade 3/4) and resulted in deaths in 12.7% of patients (18 due to COVID, 2 due to pneumonia). On average, the first infection occurred early (1.7m). The respiratory tract was the most common site of bacterial and viral infections. (57.6%). Herpes infections (4.2%), CMV viraemia (1.2%), PJP infections (4.2%) and fungal infections (5.5%) also occurred. Hypogammaglobulinemia also occurred early (1.2 months) as well as neutropenia (2-4 months). The authors recommend screening for HBV, HCV and HIV as well asCMV screening at regular intervals. Vaccinations (COVID, Influenza, VZV, Pneumococcus) should be up to date but the optimal timing is unclear. PJP and HSV/VZV prophylaxis is recommended, as well as maintaining IgG levels >400mg/dl. GCSF is recommended for grade 3 neutropenia |
| *Dima, Davis et al. 2024* | Efficacy// PFS, OS, Safety | This retrospective, real-world analysis showed an ORR of 66% in 106 patients with relapsed and refractory myeloma. One third of patients (31%) experienced infections, with severe outcomes in 46% of cases and three fatalities. The most commonly reported infections were respiratory (69%), followed by viral (51%) and bacterial (41%) infections. Infections were the most common cause of hospitalisation (15%) and dose delay (44%). Neutropenia occurred in 42% of cases. Immunoglobulins were administered in 42% of cases |
| *Frerichs, Verkleij et al. 2024* | Changes in polyclonal immunoglobulin, B‑cell count// Vaccination response, incidence on infection | This analysis evaluates the impact of weekly teclistamab on polyclonal immunoglobulins and B cell count. It describes a rapid decline in B cell count alongside a persistent reduction in polyclonal immunoglobulins. This resulted in a significantly reduced vaccine response (streptococcus pneumoniae vaccination: ORR of 7.1%). Primary immunoglobulin administration was found to drastically reduce the risk of infection compared to secondary prophylaxis following infection (54.8% vs. 5.3%). |
| *Riedhammer, Bassermann et al. 2024* | ORR// PFS, OS, DOR, Saftey | A real-world analysis of 123 patients showed impressive ORRs of 59.3%, demonstrating the efficacy and tolerability of the treatment. Infections occurred in over half of the patients (54.5%), with hospitalisation required for 26.8% of patients. Prophylaxis for PJP and HSV, as well as immunoglobulin administration, were routinely used |
| *Touzeau, Krishnan et al. 2024* | ORR// DOR, PFS, OS, Safety | The subgroup analysis of the MAJESTEC Phase 1/2 study included patients who had previously received BCMA-targeted therapy. While a ORR of 52.5% was achieved, infections were again common, affecting 70% of patients |
| *Kawasaki, Steele et al. 2024* | CRS, ICANS// length of hospital stay, hematologic toxicity, infection rate | This retrospective study analysed 27 patients, focusing on the frequency of CRS/ICANS, length of hospitalisation, haematotoxicity and infections. Infections occurred frequently alongside hematotoxicity, with a prevalence of 25–42%. |
| *Tan, Asoori et al. 2025* | ORR// PFS, OS, Safetyy | This real-world analysis of 210 myeloma patients treated with teclistamab found that 44% of patients had previously received BCMA therapy. An ORR of 67% was achieved, alongside frequent occurrences of infection (56.2%, grade 3/4: 22%). Prophylaxis for PJP and viral infections (HSV/VZV) was administered uniformly, but there was no clear statement on the frequency of antibiotic and antifungal prophylaxis. On average, infections occurred within 33 days, with frequent respiratory infections (41.4%) and viral infections (41%), which were often accompanied by hypogammaglobulinemia (69%) and immunoglobulin administration (62%). |
| *Ariane, Stéphanie et al. 2025* | Porportion and timing of Ig-substitution// infections, IgG level, Ig consumption | This analysis analysed the frequency of hypogammaglobulinemia under Teclistamab treatment and discussed the procedure, based on a retrospective analysis of 35 patients. Twenty-four of the patients received immunoglobulins after only one month (at a dose of 0.4 g/kg per month), primarily due to infections. These patients were able to maintain IgG levels of 2.6 g/L for six months. While the use of immunoglobulins is considered beneficial, concerns are raised about the poor measurement of these preparations |
| *Perrot, Hulin et al. 2024* | ORR// PFS, Safety | This analysis of 249 patients included many who would not have met the inclusion criteria for the registration study. This cohort achieved an ORR of 68.8%, with a similar side effect profile and infections in 55% of cases, which were always severe. 13% of patients discontinued treatment due to infections, 30% were hospitalised, and 61%received immunoglobulins. The infections were primarily viral (50%), bacterial (45%), or fungal (5%). |
| *Ishida, Kuroda et al. 202^2^* | Safety& Tolerability, Efficacy// DOR, PFS, OS, Safety | In this Japanese cohort of 26 patients, Teclistamab demonstrated an ORR of 76.9%. There were 64.3% of infections and 14.3% of grade 3–4 infections. Hypogammaglobulinemia was detected in the majority of patients (84.6%), with 80.8% receiving IVIG. CMV infections were observed in 11.5% of patients in this study. |
| *Mohan, Monge et al. 2024* | ORR// PFS, CRS, ICANS, Infections, Impact of IVIG | This real-world analysis examined the safety and efficacy of teclistamab in 110 patients. An ORR of 62% was achieved. CRS was reported in 56% of patients, ICANS in 11%, and infections in 40%. IVIG prophylaxis was found to significantly reduce the risk of infection (HR = 0.33, p = 0.001) |
| *Cai, Xia et al 2024* | ORR// CR, MRD, DOR, PFS, Safety | In the Chinese phase 2 cohort of the MajesTEC-1 study, 26 patients with heavily pretreated RRMM (median 5 prior therapies, follow-up 15 months) received teclistamab. The overall response rate was 76.9%, ≥CR 57.7%, MRD negativity was achieved in 90%; median DOR, PFS and OS were not reached (12-month rates: 78.5%, 68.0%, 83.5%). Infections occurred in 96% (grade ≥ 3 in 69%), mostly COVID-19 (73%) and pneumonia (38%); the incidence decreased with less frequent dosing. Hypogammaglobulinaemia was common (92%), 81% received IVIG; HBV reactivations only occurred without antiviral prophylaxis. CRS occurred in 96% (grade 1–2 only), ICANS was not observed. Neutropenia was very common (96%, grade 3/4 in 77%). Overall, high efficacy was demonstrated with manageable toxicit |
| *Razzo, Midha et al 2025* | Safety, ORR/ CR, PFS, OS, predictors of response and survival, impact prior BCMA -directed therapy | This large multicenter real-world retrospective study evaluated teclistamab in 509 heavily pretreated RRMM patients, of whom 89% would have been ineligible for the MajesTEC-1 trial. Infections occurred in 42% of patients, with serious infections in 22% and infection-related mortality in 5%. Hypogammaglobulinemia (IgG <400 mg/dL) was associated with a significantly higher risk of serious infections, while IVIG use appeared protective. Despite the high-risk population, teclistamab achieved an ORR of 53%, with manageable CRS (54%; grade ≥3: 1.4%) and ICANS (11%; grade ≥3: 2.2%). The study highlights the importance of infection prophylaxis and IVIG replacement in real-world BCMA-bispecific antibody therapy. |
| *Bros, Harel et al 2025* | Hypogammaglobulinemia management | This retrospective single-center observational study evaluated immunoglobulin replacement practices in 35 RRMM patients treated with teclistamab during early access in France. Hypogammaglobulinemia and recurrent infections emerged as major complications of BCMA-directed bispecific antibody therapy, leading to increasingly early and systematic IVIG substitution. Twenty-four patients received IVIG after a median of 1 month, and at least one infection occurred in 13 patients approximately 1 month after teclistamab initiation. Most infections were bacterial (75%), predominantly pneumonia, bronchitis, and urinary tract infections. Despite IVIG substitution, IgG levels remained persistently low over time. |
| *Snyder, Atrash et al 2025* | Efficacy and Safety | This multicenter retrospective real-world study evaluated continuous versus fixed-duration teclistamab strategies in heavily pretreated RRMM patients. Fixed-duration therapy after deep response appeared feasible, with comparable early PFS and OS despite shorter treatment exposure. The study primarily focused on treatment de-escalation and response-adapted therapy, while infections emerged as an important safety concern. Infection rates remained substantial, particularly in the fixed-duration cohort and even after treatment discontinuation, suggesting persistent immune dysfunction and delayed immune reconstitution. |
| *Stork, Radocha et al 2025* | Efficacy// ORR, PFS, OS, relative dose intensity, Safety | This real-world Czech study evaluated reduced-frequency dosing of teclistamab in 73 heavily pretreated patients with relapsed/refractory multiple myeloma. About 25% of patients received less frequent dosing (every 2–4 weeks instead of weekly), often within the first month of treatment. Despite lower dose intensity, progression-free survival remained comparable to standard weekly dosing, suggesting maintained efficacy. Reduced dosing was associated with less neutropenia, but infection rates remained high in both groups. The follow-up was limited to 4.9 months (0.3-17.9 months) |
| *Yi, Lee et al 2025* | Efficacy and Safety | This nationwide Korean retrospective real-world study evaluated teclistamab in 42 heavily pretreated patients with relapsed/refractory multiple myeloma enrolled through a Named Patient Program. The primary endpoint was efficacy and safety of teclistamab in routine clinical practice, while secondary endpoints included ORR, DoR, PFS, OS, CRS, ICANS, infections, and treatment-related toxicities. Patients had received a median of 6 prior lines of therapy, and the ORR was 66.7%, with 40.5% achieving ≥CR. After a median follow-up of 16.4 months, median PFS was 14.1 months and the 12-month OS rate was 61.7%. Hematologic toxicities and CRS were mostly manageable, but grade ≥3 infections occurred frequently (42.9%) and represented a major cause of treatment interruption and death. |
| *Smits, Groen et al 2026* | Burden of infection // Impact of IVIG and dose deescalation, PFS, OS | This retrospective single-center study evaluated the impact of IVIG supplementation and dosing interval de-escalation on infection risk in 80 patients with RRMM treated with teclistamab between 2019 and 2024. The primary objective was to assess the burden of infections using annualized infection rates and to determine whether IVIG prophylaxis and less frequent dosing reduce infectious complications. After a median follow-up of 21 months, 90% of patients experienced at least one infection and 38% developed a severe infection. IVIG supplementation significantly reduced both all-grade infections (3.15 vs 4.41 infections per patient-year) and severe infections (0.33 vs 0.93 infections per patient-year). Importantly, extending teclistamab dosing intervals was associated with a marked reduction in infection burden, with all-grade infection rates decreasing from 6.08 infections per patient-year during weekly dosing to 2.25 during bimonthly dosing, and severe infections decreasing from 0.81 to 0.10 infections per patient-year. |
| *Ssentongo, Guare et al 2026* | Incidence of infections // infection-related outcomes, risk factors. | This retrospective single-center real-world cohort study evaluated the incidence, timing, and severity of infections in 19 heavily pretreated RRMM patients receiving teclistamab between January and November 2023. With a median follow-up of 196 days, 57.9% of patients developed at least one infection, accounting for 19 infectious episodes. Most infections were severe (78.9% grade ≥3), occurred early (median time to first infection: 20 days), and frequently resulted in hospitalization and treatment discontinuation. Bacterial infections, including bacteremia, and respiratory viral infections predominated, while no PJP, HSV/VZV, or fungal infections were observed. Infection was the leading cause of permanent teclistamab discontinuation and contributed to mortality. |
| *Tan, Usmani et al 2026* | Incidence of CMV reactivation // risk factors, clinical outcomes, and overall survival. | This retrospective single-center cohort study evaluated the incidence, risk factors, and clinical significance of CMV reactivation in 177 RRMM patients treated with teclistamab between December 2022 and December 2024. CMV reactivation occurred in 38/173 monitored patients (22%), typically within the first 2–3 months of treatment (median 5 weeks). Most episodes were asymptomatic (89.5%), only four patients developed symptomatic infection, three required antiviral treatment, and no cases of CMV end-organ disease were observed. Prior CMV reactivation was the only independent predictor of subsequent CMV reactivation (OR 3.34). Importantly, CMV reactivation did not affect overall survival, and no patients discontinued teclistamab because of CMV. The findings support risk-adapted CMV surveillance rather than universal antiviral prophylaxis in teclistamab-treated patients. |
| *Costa, Bahlis et al 2025* | PFS // CR, ORR, MRD, OS, Safety | Costa et al. (MajesTEC-3) conducted a randomized, open-label phase III trial comparing teclistamab plus daratumumab with standard daratumumab-based therapy (DPd/DVd) in 587 patients with relapsed or refractory multiple myeloma after 1–3 prior lines of therapy. After a median follow-up of 34.5 months, teclistamab–daratumumab significantly improved progression-free survival, overall response rate, complete response rate, MRD negativity, and overall survival. Infections were common (96.5%), with grade 3–4 infections occurring in 54.1% of patients. Fatal infections occurred in 4.6% of patients receiving teclistamab–daratumumab, predominantly within the first six months of treatment. |
| A summary of the trial is given in the supplemental table. It includes the title of the trial. The primary and secondary endpoints are also given. Abbreviations: Graft-versus-host disease = GVHD; Overall response = ORR; Minimal residual disease = MRD; Cytokine release syndrome = CRS; Immune effector cell neurotoxicity syndrome = ICANS; Progression-free survival = PFS; Overall survival = OS; Complete response = CR; Herpes simplex infection = HSV; Herpes zoster = HZV; Cytomegalovirus = CMV; Pneumocystis jiroveci = PJP; Hepatitis B = HBV; Hepatitis C = HCV. | | |

| Summary table Elranatamab | | |
| --- | --- | --- |
| Study; type of study | **primary // secondary outcome** | **summary** |
| *Lesokhin, Tomasson et al. 2023* | ORR// DOR, PFS, OS, Safety | The MagnestisMM-1 study analysed 88 patients, with the primary endpoints being dose-limiting toxicities, ORR and duration of response (DOR). No dose-limiting toxicities were observed, with an ORR of 63.6% and a DOR of 17.1 months. Infections occurred in 74.5% of patients, with severe progression occurring in 27.3%, and PJP, CMV and three deaths occurring |
| *Bahlis, Costello et al. 2023* | Safety& tolerability, ORR, DOR//PFS, OS Safety profil | This Phase 2 data from the MagnetisMM-3 study shows the ORR for 123 patients treated with elranatamab. An ORR of 61% was observed. The most common side effect was infection, occurring in 69.9% of patients, with 39.8% of cases being severe. Infections were the most common reason for dose delays (50.4%), leading to death in eight patients. Hypogammaglobulinaemia occurred frequently in 75.5% of patients. Most patients received antiviral prophylaxis (87%), and almost half received PJP prophylaxis (49.6%). Fewer patients received antifungal prophylaxis (11.4%) and antibacterial prophylaxis (5.7%). Neutropenia also occurred in 15.4% of patients |
| *Tomasson, Iida et al. 2024* | ORR// DOR, PFS, OS, Safety | This long-term follow-up of the MagnetisMM-3 study had a follow-up period of 14.7 months. PFS of 17,2 months and OS of 24.6 months has been achieved, with an ORR of 61%. The long-term analysis also revealed frequent infections (70.7%). |
| *Iida, Ito et al. 2024* | Safety& tolerability, ORR, DOR//PFS, OS Safety profil | This analysis presents a subgroup analysis of Japanese MagnetisMM2/3 patients (n = 16). Hypogammaglobulinaemia (50–25%) and infections (75–58.3%) were equally prevalent. This long-term follow-up of the MagnetisMM-3 study had a follow-up period of 14.7 months. PFS of 17.2 months and OS of 24.6 months were reached, with an ORR of 61%. The long-term analysis also revealed frequent infections (70.7%). |
| *Malard, Bobin et al. 2024* | ORR// ≥VGPR rate, 1‑year PFS & OS, DOR) | This retrospective analysis examined the treatment of 101 patients in the compassionate use programme with elranatanab in a real-world setting. The analysis revealed an ORR of 51.5%, with at least one infection occurring in 49% of patients. Fungal infections were reported in three patients, 19 bacterial infections were reported, and 11 infections were not documented. Only 50% of patients received intravenous immunoglobulins. |
| *Soltantabar, Hibma et al 2026* | Safety | This pooled analysis evaluated 324 RRMM patients treated across the MagnetisMM-1, -2, -3, and -9 trials. The study investigated whether higher elranatamab exposure was associated with increased toxicity, particularly grade ≥3 infections and hematologic adverse events. Grade ≥3 infections occurred in 41.7% of patients, while grade ≥3 neutropenia, anemia, and thrombocytopenia occurred in 48.1%, 42.6%, and 26.2%, respectively. No significant exposure–toxicity relationship was identified. Immunoglobulin supplementation emerged as the only significant protective factor associated with reduced grade ≥3 infection risk, although this finding should be interpreted cautiously due to late and inconsistent implementation across studies. Severe cytopenias mainly occurred during early treatment cycles, whereas severe infections were distributed across later treatment periods. |
| *Piron, Rodrigo et al 2025* | ORR, PFS, OS, DOR, Infection-free survival | This multicenter retrospective real-world study from the U.S. Multiple Myeloma Immunotherapy Consortium evaluated the efficacy and safety of commercial elranatamab in 130 heavily pretreated patients with relapsed/refractory multiple myeloma treated between August 2023 and March 2025. The primary endpoints were overall response rate (ORR), progression-free survival (PFS), overall survival (OS), duration of response (DOR), and infection-free survival (IFS). Patients had received a median of six prior lines of therapy; 91% were triple-class refractory, 49% were penta-refractory, and 49% had prior BCMA-directed therapy. With a median follow-up of 7.5 months, ORR was 65%, including ≥CR in 36%. Median PFS, OS, and DOR were 4.3, 14.6, and 12.2 months, respectively. Infections occurred in 38% of patients, and intravenous immunoglobulin (IVIG) supplementation was independently associated with improved infection-free survival and infection-free PFS. CRS occurred in 40% and ICANS in 17% of patients |
| A summary of the trial is given in the supplemental table. It includes the title of the trial. The primary and secondary endpoints are also given. Abbreviations: Graft-versus-host disease = GVHD; Overall response = ORR; Minimal residual disease = MRD; Cytokine release syndrome = CRS; Immune effector cell neurotoxicity syndrome = ICANS; Progression-free survival = PFS; Overall survival = OS; Complete response = CR; Herpes simplex infection = HSV; Herpes zoster = HZV; Cytomegalovirus = CMV; Pneumocystis jiroveci = PJP; Hepatitis B = HBV; Hepatitis C = HCV. | | |

| Summary Table, Linvoseltamab | | |
| --- | --- | --- |
| Study; type of study | **primary // secondary outcome** | **summary** |
| *Bumma, Richter et al. 2024* | ORR// DOR, PFS, OS, Safety | The efficacy of linvoseltamab is being analysed. An ORR of 71% was achieved with a dose of 200 mg. CRS and ICANS occurred frequently, with infections observed in 74.4% of patients (33.3% grade 3, 2.6% were grade 4). Opportunistic infections, including CMV reactivation or infection, occurred in 10.3% of patients; the most frequent was PJP (4%). Following the introduction of PJP prophylaxis, no additional cases of PJP were observed. The rate of grade 3–4 infections was 20–22% between 0–6 months and 4–8% between 6–15 months. Three treatment-related deaths occurred due to infection: Pneumocystis jirovecii pneumonia (PJP), progressive multifocal leukoencephalopathy, and pseudomonal sepsis. Most treatment-emergent adverse events (TEAEs) leading to discontinuation were infection-related (9.4%), and 75 out of 117 patients (64.1%) received intravenous immune globulin |
| *Lee, Zonder et al 2025* | ORR//DOR, PFS, OS, Safety | This updated analysis of the phase 1/2 LINKER-MM1 trial evaluated long-term efficacy and safety of linvoseltamab in 117 heavily pretreated RRMM patients with a median follow-up of 21.3 months. Linvoseltamab achieved an ORR of 71%, ≥CR in 52%, MRD negativity in 94% of evaluable CR patients, median DOR of 29.4 months, and median OS of 31.4 months. Responses were maintained across high-risk subgroups including penta-refractory disease, high-risk cytogenetics, and extramedullary disease, although outcomes were poorer in patients with high disease burden and EMP. CRS occurred in 46% and ICANS in 8%, mostly low grade. Infections were common (75% any grade; 48% grade ≥3), including opportunistic infections in 11% and CMV infections in 5%, with infection-related deaths reported in seven patients. Infection incidence decreased after 6 months of treatment, coinciding with transition to less frequent Q4W dosing in responding patients. |
| A summary of the trial is given in the supplemental table. It includes the title of the trial. The primary and secondary endpoints are also given. Abbreviations: Graft-versus-host disease = GVHD; Overall response = ORR; Minimal residual disease = MRD; Cytokine release syndrome = CRS; Immune effector cell neurotoxicity syndrome = ICANS; Progression-free survival = PFS; Overall survival = OS; Complete response = CR; Herpes simplex infection = HSV; Herpes zoster = HZV; Cytomegalovirus = CMV; Pneumocystis jiroveci = PJP; Hepatitis B = HBV; Hepatitis C = HCV. | | |
|  | | |

| Summary Table, AMG420-BITE | | |
| --- | --- | --- |
| Study; type of study | **primary // secondary outcome** | **summary** |
| *Topp, Duell et al. 2020* | Safety, ORR// DOR, Safety | The safety and efficacy of the BCMA-targeted antibody AMG 420 was analysed in a dose-finding cohort of 42 patients (0.2-800ug/d for 10 6 week cycles). The primary endpoints were dose-limiting toxicity, ORR and MRD negativity. Severe adverse events occurred in 48% of patients, including 14 cases of infection and polyneuropathy. The tolerable dose was 400 μg/d. An ORR of 70% was detected at this dose level. Due to the short half-life, daily infusion is necessary |
| A summary of the trial is given in the supplemental table. It includes the title of the trial. The primary and secondary endpoints are also given. Abbreviations: Graft-versus-host disease = GVHD; Overall response = ORR; Minimal residual disease = MRD; Cytokine release syndrome = CRS; Immune effector cell neurotoxicity syndrome = ICANS; Progression-free survival = PFS; Overall survival = OS; Complete response = CR; Herpes simplex infection = HSV; Herpes zoster = HZV; Cytomegalovirus = CMV; Pneumocystis jiroveci = PJP; Hepatitis B = HBV; Hepatitis C = HCV. | | |

| Summary Table, ALNUCTAMAB | | |
| --- | --- | --- |
| Study; type of study | **primary // secondary outcome** | **summary** |
| *Bar, Martin et al 2026* | Safety, Tolerability // Efficacy | In this first-in-human phase I study, the BCMA-directed bispecific antibody alnuctamab demonstrated meaningful clinical activity in heavily pretreated RRMM patients (ORR 58.9%; 71.4% at the RP2D) with frequent but predominantly low-grade infections (64.2%), while grade ≥3 infections occurred in 14.7%. Opportunistic infections were uncommon, including asymptomatic CMV reactivation (4%) and a single case of fungal esophagitis, suggesting that intensive prophylaxis and immunoglobulin supplementation may mitigate infectious toxicity. |
| A summary of the trial is given in the supplemental table. It includes the title of the trial. The primary and secondary endpoints are also given. Abbreviations: Graft-versus-host disease = GVHD; Overall response = ORR; Minimal residual disease = MRD; Cytokine release syndrome = CRS; Immune effector cell neurotoxicity syndrome = ICANS; Progression-free survival = PFS; Overall survival = OS; Complete response = CR; Herpes simplex infection = HSV; Herpes zoster = HZV; Cytomegalovirus = CMV; Pneumocystis jiroveci = PJP; Hepatitis B = HBV; Hepatitis C = HCV. | | |

| Summary Table, PAVURUTAMAB | | |
| --- | --- | --- |
| Study; type of study | **primary // secondary outcome** | **summary** |
| *Lee, Plattel et al 2026* | Safety, Tolerability // Efficacy | Lee et al. conducted a prospective multicenter phase 1/1b study evaluating the BCMA×CD3 bispecific T-cell engager pavurutamab (AMG 701) in 172 heavily pretreated patients with relapsed/refractory multiple myeloma. Pavurutamab demonstrated encouraging clinical activity, with an ORR of 65.8% at the recommended phase 2 dose and a median PFS of 16.8 months. Infections were common, occurring in 70.9% of patients, including 30.8% grade 3–4 infections, 12.2% opportunistic infections, and 4.1% fatal infections |
| A summary of the trial is given in the supplemental table. It includes the title of the trial. The primary and secondary endpoints are also given. Abbreviations: Graft-versus-host disease = GVHD; Overall response = ORR; Minimal residual disease = MRD; Cytokine release syndrome = CRS; Immune effector cell neurotoxicity syndrome = ICANS; Progression-free survival = PFS; Overall survival = OS; Complete response = CR; Herpes simplex infection = HSV; Herpes zoster = HZV; Cytomegalovirus = CMV; Pneumocystis jiroveci = PJP; Hepatitis B = HBV; Hepatitis C = HCV. | | |

| Summary Table, Talquetamab | | |
| --- | --- | --- |
| Study; type of study | **primary // secondary outcome** | **summary** |
| *Chari, Minnema et al. 2022* | ORR// VGPR or better, DOR, PFS, Safety | A total of 232 patients have received talquetamab. Four dose-limiting adverse effects were observed: increased lipase levels in three patients, and maculopapular rash in one patient. The dosage used was 800 μg/kg every two weeks and 405 μg/kg weekly. An ORR of 64–70% was reported. CRS occurred in 77-80% of patients. Haematological effects occurred, but were rarely severe. Infections occurred in 47% of patients, with severe cases occurring in 7% of patients at a dose of 405 μg/kg. There was no CMV reactivation or infection. Hypogammaglobulinaemia occurred in 87% of cases. Skin toxicity occurred in 67% of patients, nail toxicity in 57%, and loss of taste in 63%. |
| *Schinke, Dhakal et al. 2024* | ORR//PFS, Safety | This real-world analysis included 11 healthcare providers to understand talquetamab dosing and symptom management in real-world settings. Step-up was performed on an inpatient basis in five cases, on an outpatient basis in three cases, and in two cases on both bases. The most common dosing regimen was administered every two weeks. Symptom management focused on oral mucosal toxicity, for which dexamethasone, nystatin, zinc and a vitamin B complex were used, as well as local steroids for skin and nail toxicity. These measures were taken to prevent local inflammation |
| *Chari, Touzeau et al. 2025* | ORR// CRS, Infections, other AE | This multicentre study involved 375 patients who were treated with talquetamab. An ORR of 74–69% was achieved. In addition to cytokine release syndrome (CRS) (reported in 75% of patients), taste disturbances (reported in 72%) and infections (reported in 59–76%) were also reported, as well as neutropenia (reported in 21–47% of patients). Opportunistic infections (e.g. oesophageal candidiasis, adenovirus, herpesvirus 6, herpes ophthalmicus, VZV, CMV, fungal sepsis and viral retinitis) occurred in 3% of patients. Hypogammaglobulinaemia occurred in 8–19% of patients within two to three months of starting immunoglobulin treatment. T cell counts were also analysed, revealing a higher frequency in responders and a lower proportion of LAG-3- and CD38/CD8-expressing T cells. |
| *Frenking, Riedhammer et al. 2025* | ORR// DOR,PFS,OS, Safety | The multicentre German real-world study (n = 138) investigated talquetamab in heavily pretreated patients with relapsed/refractory multiple myeloma (median 6 prior therapies). The ORR was 56.3%, with a CR/sCR rate of 25%. Median PFS was 5.2 months, and OS was 10.3 months. CRS occurred frequently (70.3%), but was mostly mild, ICANS was rare. Infections were common (55.8%, grade ≥3 in 16.7%), including CMV reactivations. The study confirms the efficacy and safety profile from clinical trials. |
| *Iida, Sunami et al 2025* | Safety// ORR, DOR, TTR | In this single-arm, open-label Phase 1 study (Japan), 15 heavily pretreated RRMM patients received s.c. talquetamab (135 µg/kg QW, 400 µg/kg QW or 800 µg/kg Q2W). Safety/DLT was evaluated as the primary endpoint, with efficacy (including ORR, DOR, TTR) and PK/PD as secondary endpoints. After a median follow-up of 9.0 months, the ORR was 60% with a manageable toxicity profile (CRS exclusively grade 1; no ICANS). Infection occurred in 53.3%. |
| *Dhakal, Akhtar et al 2025* | Feasability and safety | Talquetamab bridging prior to BCMA CAR-T therapy was feasible and effective in a heavily pretreated real-world RRMM population, including many trial-ineligible patients. A 71% response rate during bridging and an 88% response after CAR-T were observed, while severe CRS, ICANS, and grade ≥3 infections remained infrequent. However, interpretation is limited by the retrospective non-randomized design, heterogeneous multicenter management, and limited long-term infection follow-up |
| *Schinke, Rodriguez-Otero et al 2025* | Infections and parameters of humoral immunity | This full publication from the phase 1/2 MonumenTAL-1 trial analyzed infections and humoral immunity in 339 heavily pretreated RRMM patients receiving talquetamab. Any-grade infections occurred in 59–73% of patients, whereas grade 3/4 infections remained comparatively low (16–28%) relative to BCMA-directed bispecific antibodies. Respiratory infections, particularly COVID-19 and upper respiratory tract infections, predominated, while opportunistic infections and infection-related deaths were infrequent (1.5%). Importantly, most severe infections occurred early during therapy, predominantly within the first treatment cycles and the first 100 days, after which infection risk declined substantially. Neutrophil counts recovered rapidly after cycle 2, B-cell levels remained stable, and polyclonal IgG levels increased over time, supporting preservation of humoral immunity with GPRC5D targeting |
| *Al Hadidi, Szabo et al 2025* | Efficacy/ Safety | This multicenter real-world study evaluated talquetamab in 114 heavily pretreated RRMM patients, most of whom were triple-class refractory, penta-refractory, and previously exposed to BCMA-directed therapies. Talquetamab achieved an ORR of 73%, with a median PFS of 10 months and 6-month OS of 86%, confirming efficacy outside clinical trials. CRS occurred in 54% of patients and ICANS in 9.8%, predominantly low grade. Infections occurred in 27% of patients, with grade ≥3 infections in 18%, mainly viral respiratory infections, while no fatal fungal infections were observed. More than half of infectious episodes required hospitalization, although no infection-related deaths occurred. IVIG prophylaxis was frequently used (61%) and may have contributed to the lower infection incidence compared with BCMA-directed bispecific antibodies. The study additionally highlighted progressive weight loss. |
| *Janakiram, Tan et al 2026* | Real-world safety, tolerability, and effectiveness | This international multicenter real-world study evaluated talquetamab in 151 heavily pretreated RRMM patients across five countries. Patients had received a median of 6 prior lines of therapy, 53% were penta-refractory, and 75% had prior BCMA-directed treatment. With a median follow-up of 12.7 months, talquetamab demonstrated sustained efficacy with an ORR of 67.6%, median PFS of 7 months, and 12-month OS of 65%. Infections occurred in 51% of patients, with 48% of infected patients requiring hospitalization. The most common infections were upper respiratory tract infections, pulmonary infections, viral reactivations, bacteremia, and urinary tract infections. CMV reactivation occurred in 11%, although no CMV end-organ disease was observed and no infection-related deaths were reported. Grade 3–4 neutropenia occurred in 36% of patients. |
| A summary of the trial is given in the supplemental table. It includes the title of the trial. The primary and secondary endpoints are also given. Abbreviations: Graft-versus-host disease = GVHD; Overall response = ORR; Minimal residual disease = MRD; Cytokine release syndrome = CRS; Immune effector cell neurotoxicity syndrome = ICANS; Progression-free survival = PFS; Overall survival = OS; Complete response = CR; Herpes simplex infection = HSV; Herpes zoster = HZV; Cytomegalovirus = CMV; Pneumocystis jiroveci = PJP; Hepatitis B = HBV; Hepatitis C = HCV; Duration of response= DOR; TTR= Time to response. | | |

| Summary Table, Cevostamab | | |
| --- | --- | --- |
| Study; type of study | **primary // secondary outcome** | **summary** |
| *Trudel, Cohen et al. 2021* | Dose-limiting toxicities, safety// DOR, CRS, infections | A total of 167 patients were given cevostamab at various escalating doses. The ORR was 43.1%. In addition to CRS and ICANS, infections were prevalent (53.9%), with 22.2% of cases classified as severe. Haemophagocytic lymphohistiocytosis and one case of Pseudomonas sepsis were also reported. |
| *Lesokhin, Richter et al. 2022* | Safety& dose limiting toxicity// Efficacy, Safety | Sixteen patients received 17 cycles of intravenous cevostamab. A CR was achieved in 58.8% of cases. Only two patients developed an infection after completing the full 17 cycles |
| A summary of the trial is given in the supplemental table. It includes the title of the trial. The primary and secondary endpoints are also given. Abbreviations: Graft-versus-host disease = GVHD; Overall response = ORR; Minimal residual disease = MRD; Cytokine release syndrome = CRS; Immune effector cell neurotoxicity syndrome = ICANS; Progression-free survival = PFS; Overall survival = OS; Complete response = CR; Herpes simplex infection = HSV; Herpes zoster = HZV; Cytomegalovirus = CMV; Pneumocystis jiroveci = PJP; Hepatitis B = HBV; Hepatitis C = HCV. | | |

| Summary Table, all bispecific antibody | | |
| --- | --- | --- |
| Study; type of study | **primary // secondary outcome** | **summary** |
| *Jourdes, Cellerin et al. 2024* | Significant infections// rate and severity of infections, risk factors, impact on bispecific antibody | This retrospective study involved analysing 229 patients receiving teclistamab, elranatamab and talquetamab in a real-world setting across 14 French centres. A total of 234 infections were reported, 53% of which were of grade ≥3 severity. 70% of patients had at least one infection, but this figure was only 51% for GPRC5D BITE patients. Hospitalisation due to infection was necessary in 56% of cases. Intensive care was required in 13% of cases, with a 9% mortality rate. The most common infections were respiratory (50%) and septic (15%) infections. Hypogammaglobulinemia was present in 61% of infections, but only in 21% of patients receiving immunoglobulin. Cortisone, which is used in CRS and ICANS, increases the risk of infection. Patients with GPRC5D-targeting therapies had a lower risk of infection. Antibiotic prophylaxis was protective too (HR 0.65). Infections led to dose adjustments in almost half of patients (44%), therapy discontinuation in 13%, and therapy delays in 30%. |
| *Li, Zhang et al. 2025;* | Incidence of COVID 19 infections.// protein expression and pathways | This analysis examined plasma proteins to determine the risk of severe progression of the disease under different myeloma therapies. It was found that the proportion of severe cases of progression was significantly higher under BITE. Several differentially expressed proteins (DEPs) were identified that were present at higher levels in BITE patients and were associated with cytokine activity and leukocyte migration. In particular, the leukaemia inhibitory factor was found to be increased. |
| *Cohen, Magen et al. 2025* | Safety, ORR// CR, DOR, PFS, MRD, infections, CRS, ICANS | The combination of talquetamab and teclistamab in RedirecTT1 has an ORR of 80%, with a particularly good response to extramedullary disease (75%), though this is accompanied by corresponding toxicity. Dose-limiting toxicities were reported in three patients, presenting with oral herpes and LFT elevation. Neutropenia was observed in 73% of patients, primarily presenting severely (68%), alongside skin/nail or taste toxicity (52–62%), and CRS (79%), with grade 3/4 occurring in 2% of cases. 15% of patients died, 12% of whom due to infection complications. Infections occurred in almost all patients (89%), with severe courses in 64% of cases. 82% received antiviral prophylaxis and 48% received PJP prophylaxis. 63% received the SARS-CoV-2 vaccine, yet 40% had SARS-CoV-2 infections, 2% of which were fatal. Up to 70% of patients had hypogammaglobulinaemia. |
| *Hammons, Szabo et al. 2024* | Incidence and severity of grade 3 infections// Etiology and site of infection, risk factors, cumulative infections. | The analysis included 90 patients with BCMA and GPRC5D bispecific antibodies. Similar frequencies of tocilizumab and steroid use were observed in both cohorts due to CRS/ICANS. During the follow-up period of 193 days, 127 infections occurred. Of these, 99 occurred under BCMA bsAb and 24 under GPRC5D bsAb. The infection rate differed between the two types of bsAb (0.57 vs. 0.62). A stepwise increase in the cumulative incidence of infection was observed within the first six months. A continuous but slower increase was detected until month 18. Bacterial infections occurred in 5% of cases, viral infections in 39%, and fungal infections in 7%. GPRC5D bsAb-related infections were milder than BCMA-related infections (grade3/ 4: 42% vs. 65%). The risk was significantly lower with GPRC5D bsAb (HR 1.36). |
| *Mohan, Szabo et al. 2025* | Infection-free survival// grade 3 infections, PFS, OS | Patients were treated with BCMA bsAb in a real-world setting. The impact of immunoglobulin substitution on infection rates was analysed. Infection prophylaxis was administered in accordance with institutional guidelines. Of the 225 patients included in the study, 59% received no primary IVIG, while 41% received primary immunoglobulins. A total of 288 infections were detected, with a median time to infection of 97 days. Bacterial and viral infections were once again the most common, with isolated fungal infections occurring, as well as five cases of PJP pneumonia despite the patients not receiving PJP prophylaxis. Administering primary immunoglobulins did not result in significant protection, but using them again after an initial infection significantly reduced the risk of future infections. Bacterial infections in particular were significantly reduced. Additionally, mortality was reduced by primary immunoglobulin use, and overall survival (OS) was prolonged (44 months versus 16 months). The risk of infection increased with lymphopenia, tocilizumab use and prior grade 3 infection. |
| *Lancman, Parsa et al. 2023* | Incidence of grade 3-5 infections// IVIG use | A total of 37 patients who had been treated with anti-BCMA bispecific antibodies were included in the study. CRS was reported in 64% of cases. 92% of patients received IVIG. VZV prophylaxis was used universally, but routine antibacterial, antifungal and PJP prophylaxis were not. A total of 118 infections were documented, with viral (46%) and bacterial (43%) infections being the most common, followed by fungal infections (11%). CMV reactivation and infection were documented. Respiratory infections were the most common. 27% of infections occurred in the first month, with a median time to first infection of 3.8 months. |
| *Uttervall, Tätting et al. 2024* | ORR, OS// incidence of infections and timing, severity of infections | This real-world analysis of BCMA bispecific antibodies in Sweden examines their safety and efficacy. The analysis includes 58 patients. CRS occurred in over half of the patients (62%), while only one patient experienced ICANS. Neutropenia occurred in 52% of cases (43% grade 3/4). Infections were reported in 28 patients (53%), with a total of 57 infections. The majority of these were respiratory infections, but viral reactivations (HSC/VZV/CMV) and two cases of PJP were also reported in patients without prophylaxis. There were six treatment discontinuations and two deaths due to infection. In sweden, immunoglobulins are not routinely administered as a primary treatment, but rather after infection. Patients received influenza and SARS-CoV-2 vaccinations. Antiviral and PJP prophylaxis were also administered, as were oral antibiotics to patients with severe neutropenia |
| *Sim, Longhitano et al. 2023* | Incidence and severity of infections// type of pathogens, Infection timing, immune parameter | All patients who participated in the study between 2018 and 2022 were included, and their medical records were analysed to identify any infectious complications. VZV/HSV and PJP prophylaxis were used routinely. A total of 39 patients were included in the study. CRS occurred in 72% of patients and ICANS in 2%. Hypogammaglobulinaemia was documented in 87% of patients, 53% of whom received IVIG. 90% of patients had at least one infection. A total of 111 infections occurred, with a median of two cycles. Respiratory infections (41%) were once again the most common, followed by gastrointestinal infections (7%). Viruses were detected in 58% of cases, while bacteria were detected in 39% of cases. The most frequent viral infections were rhinovirus and enterovirus (36%), followed by CMV (18%) and adenovirus (18%). The most frequently detected bacteria were Salmonella, E. coli, Pseudomonas, Clostridium difficile and Enterococcus faecalis |
| *Contejean, Janssen et al. 2023* | Disproportionality in infection // subgroup analysis by infection type, severity, timing | The global, pharmacovigilance-based study was evaluated in terms of its risk of infection. Anti-BCMA bispecific antibodies were found to double the risk of infection. These antibodies cause infections more frequently than other biologics due to their effect on plasma cells, hypogammaglobulinaemia, and pronounced immunosuppression. Increased vigilance is necessary to prevent infection and provide immunological support with immunoglobulins |
| *Kowalski, Lykon et al. 2025;* | CRS-incidence and severity// ORR, ICANS, infections, safety | The study investigated the prophylactic administration of tocilizumab (8 mg/kg i.v., 24 hours before the start of therapy) in 72 patients with relapsed/refractory multiple myeloma who were treated with bispecific antibodies (teclistamab, elranatamab or talquetamab). The aim was to reduce the risk of cytokine release syndrome (CRS). The results show a significant reduction in the CRS rate to 14% (exclusively grade 1–2) and a low rate of neurotoxic events (ICANS: 8%, of which 2 cases were grade 3). The overall ORR was 66% (including 38% CR), and the efficacy of the therapy was maintained despite tocilizumab. Hypogammaglobulinemia occurred in 82% of patients, infections in 47%, but there were no therapy-related deaths. Prophylactic tocilizumab administration proved to be safe, effective in preventing CRS and practical for outpatient use. |
| *Choi, Byun et al 2024* | PFS// OS, Safety | In this real-world analysis, BiTE therapy showed significantly prolonged PFS compared to SOC (19.2 vs. 5.4 months) in heavily pretreated RRMM patients, with comparable OS. Side effects were predominantly mild CRS, rarely neurotoxicity, but significantly more infections (81% vs. 49%). Non-BCMA-targeted BiTEs achieved better early survival rates than BCMA-targeted BiTEs. |
| *Park, Jang et al 2025* | Infection | Retrospective single-centre analysis from Korea (n=61) on infections under bispecifics in RRMM: 66% of patients had ≥1 infection (69 events in total), predominantly severe (grade ≥3: 80%). The most common site was the respiratory tract (46%) with primary viral (67%) infection; CMV played a role (reactivation 49%). PJP occurred in 7 cases, invasive fungal infections in 2 cases. Infection-related mortality was 8% (5 deaths). BCMA-targeted and combination regimens showed higher CMV rates. Prophylaxis at the centre: valaciclovir (HSV/VZV), TMP-SMX (PJP), risk-adapted antibacterial/antifungal prophylaxis; IVIG for hypogammaglobulinaemia (33%). CRS was common, mostly mild (61%, predominantly grade 1–2), ICANS 0; neutropenia common (61%, grade 3/4: 48%). |
| *Lancmann, Parsa et al 2024* | Infection | Retrospective analysis of anti-BCMA bispecifics in RRMM (n=37; median 7 prior therapies; follow-up 18.6 months) showed 118 infections, 26 of which were grade 3–5 (41%), mostly respiratory; viral 46% (COVID-19 43%, CMV 22%), bacterial 43%, fungal 11% (1 PJP). Two infection-related deaths (COVID-19, sepsis). All responders had profound, persistent hypogammaglobulinaemia; IVIg administration (400 mg/kg q4w) reduced severe infections by ~90% (IRR 0.10; p=0.03). Prophylaxis: VZV only; no bacterial, antifungal or PCP prophylaxis. ORR 70%, ≥VGPR 68%, sCR MRD-neg 41%; median PFS 17, OS 26 months. CRS 64% (1×grade 3), no ICANS; neutropenia 73% (grade 3/4 62%). Primary endpoint: rate of grade 3–5 infections ‘on’ vs. ‘off’ IVIg. |
| *Banerjee, Marsal et al 2021* | Tocilizumab time point// CRS, ICANS, Infectionrate, PFS, OS | A retrospective single-centre analysis (UCSF) of BCMA-targeted CAR-T therapies in RRMM (n = 50; median follow-up 15.3 months) investigated the influence of the timing of tocilizumab (Toci) administration in CRS. Thirty-eight patients (76%) received Toci (median 12 hours after CRS onset): 19 ‘early’ (≤12 hours) vs. 19 ‘late’ (>12 hours); 12 without Toci. The median duration of CRS was significantly shorter with early Toci administration (19 hours vs. 85 hours), with no differences in the frequency or severity of CRS, ICANS, infections or other toxicities. |
| *Kumar, Mateos et al 2026* | ORR// DOR, PFS, OS, Safety | This phase II study evaluated dual targeting with talquetamab and teclistamab in 90 patients with drug-resistant extramedullary RRMM. The combination achieved an ORR of 79% despite the high-risk population. However, toxicity remained substantial, with grade 3/4 adverse events in 76% and grade 3/4 infections in 31% of patients. Five infection-related deaths were reported during follow-up. |
| *Cani, Scott et al 2025* | Incidence and timing of infections// infections characteristics, effect of IVIG, reduced dosing | This retrospective single-center study analyzed infectious complications in 158 RRMM patients receiving bispecific antibodies. Clinically relevant infections occurred in 46% of patients, with grade ≥3 infections in 25% and infection-related mortality in 4%. Viral respiratory infections and CMV reactivations predominated, particularly with BCMA-directed therapies. IVIG administration significantly reduced severe infections and emerged as the only independent protective factor in multivariate analysis. Reduced dosing frequency was also associated with lower infection incidence over time. |
| *Wang, Li et al 2026* | TTNT// OS, Safety | This multinational propensity score-matched real-world study compared 188 patients treated with elranatamab and 188 treated with teclistamab. Both BCMA bispecific antibodies demonstrated comparable effectiveness, with similar TTNT (11.0 vs 12.3 months) and 3-year OS rates (~59%). Teclistamab showed longer TTNT in selected high-risk subgroups, including patients with thrombocytopenia and >5 prior treatment lines. Toxicity profiles differed: grade ≥3 neutropenia was more common with teclistamab, whereas CRS occurred more frequently with elranatamab. Infection and ICANS rates were comparable between groups, supporting individualized treatment selection in RRMM. |
| *Jelinek, Zihala et al 2026* | B-cell and plasma-cell depletion patterns // infection rates, IVIG, immune reconstitution, longitudinal immune profiling. | This multicenter translational study investigated the mechanisms underlying the higher infection risk associated with anti-BCMA compared with anti-GPRC5D bispecific antibodies in relapsed/refractory multiple myeloma. The study included 178 patients treated with anti-BCMA (teclistamab/elranatamab) or anti-GPRC5D (talquetamab) bispecific antibodies. Anti-BCMA therapy was associated with significantly higher rates of any-grade infections (79% vs 54%) and more frequent IVIG use (71% vs 33%). Using flow cytometry and single-cell RNA sequencing, the authors demonstrated that BCMA, unlike GPRC5D, is expressed throughout the B-cell lineage, leading to profound and persistent depletion of mature B cells, B-cell precursors, and normal plasma cells. In contrast, GPRC5D-targeting therapy largely spared normal B-cell compartments. The study provides important mechanistic insight into infection susceptibility under BCMA-targeted therapy and supports individualized prophylactic and treatment strategies |
| *Piron, Rodrigo et al 2025* | Risk of infection //  Infection characteristics, OS, PFS, NRM | This retrospective single-center study evaluated whether preemptive immunoglobulin (IG/IVIG) prophylaxis reduces infections in patients receiving anti-BCMA bispecific antibodies (mainly teclistamab and elranatamab). The primary endpoint was infection risk during the first 9 months of therapy, while secondary endpoints included severe infections, time to first infection, and infection characteristics. A total of 66 patients were included: 42 received reactive/secondary IG supplementation and 24 received preemptive IG prophylaxis according to international guidelines. Preemptive IG was initiated much earlier (median 1.6 vs 7.8 months) and was associated with a 66% reduction in all-grade infections (HR 0.34; P=0.0002) and a 76% reduction in grade ≥3 infections (HR 0.24; P=0.008). Infections also occurred significantly later in patients receiving preemptive supplementation (median 5.2 vs 1.8 months) |
| *Bilbao, Anson et al 2025* | Incidence of Campylobacter infection | This retrospective single-center real-world study evaluated the incidence and characteristics of *Campylobacter* spp. infections in 85 patients with RRMM treated with BCMA- and GPRC5D-directed bispecific antibodies between 2021 and 2025. Nine patients (10.5%) developed infection, corresponding to an incidence rate of 10.5 per 100 person-years, with more than half of cases presenting as invasive bacteremia. The median time to infection was 185 days. Significant declines in IgG and IgA levels during therapy highlighted the role of treatment-induced hypogammaglobulinemia as a potential risk factor. |
| A summary of the trial is given in the supplemental table. It includes the title of the trial. The primary and secondary endpoints are also given. Abbreviations: Graft-versus-host disease = GVHD; Overall response = ORR; Minimal residual disease = MRD; Cytokine release syndrome = CRS; Immune effector cell neurotoxicity syndrome = ICANS; Progression-free survival = PFS; Overall survival = OS; Complete response = CR; Herpes simplex infection = HSV; Herpes zoster = HZV; Cytomegalovirus = CMV; Pneumocystis jiroveci = PJP; Hepatitis B = HBV; Hepatitis C = HCV. | | |

| Summary Table, Idecabtagene Vicleucel | | |
| --- | --- | --- |
| Study; type of study | **primary // secondary outcome** | **summary** |
| *Munshi, Anderson et al. 2024* | ORR//CR, MRD, DOR, PFS, OS, Safety | The Phase 2 study aims to evaluate the efficacy and safety of ide-cel, with the primary endpoint being the ORR. A total of 128 patients received ide-cel. CR was reported in 33% of patients, giving an ORR of 73%. Adverse events included neutropenia (91%), CRS (84%) and neurotoxicity (18%). Infections occurred in 69% of patients, with 22% experiencing severe cases. Immunoglobulin, antimicrobial therapy and G-CSF use was common. 34% of patients died, with 3% of these deaths being attributed to infectious complications. |
| *Hansen, Sidana et al. 2023* | ORR//PFS, OS, Safety | This retrospective study analysed ide-cel in a real-world setting. A total of 196 patients underwent leukapheresis, of whom 159 received ide-cel. An ORR of 84% was reported. The side effect profile is consistent with that observed in the approval study. CRS occurred in 83% of patients and ICANS in 18%. 88% experienced neutropenia, and grade 3/4 neutropenia was present for ≥30 days after infusion in 60% of patients. Infection occurred in 34% of patients, with 20% experiencing bacterial infection, 16% viral infection and 1% fungal infection. 19% of patients died; three of these deaths were due to complications from SARS-CoV-2 infection. |
| *Lin, Raje et al. 2023* | Safety// ORR, DOR, PFS, OS | This phase 1 study demonstrated sustained remissions (ORR 75.8%) with no new safety concerns emerging. Sixty-seven patients underwent leukapheresis and 62 received Ide-Cel infusion. The most common adverse event (AE) was grade 3/4 neutropenia (88.7%). CRS occurred in 75.8% of patients (6.5% grade 3/4), and ICANS in 37.1%. Infections occurred in 75.8% of patients, with 22.6% grade 3/4. Secondary malignancy was reported in eight patients. (ORR: 75%; infections: 23%) |
| *Hashmi, Hansen et al. 2024* | Early progression, death// associated factors | This retrospective study evaluated factors associated with early progression in 215 patients. Forty-three patients experienced progression. The presence of EMD, elevated ferritin at lymphodepletion, plasma-cell leukaemia and the cytogenetic risk marker t(4;14) were identified as risk factors for progression. The presence of three or more of these markers had a negative impact on PFS and OS. There was no difference in the rate of infection between responders and non-responders |
| *Ailawadhi, Arnuf et al. 2024* | PFS// ORR, OS, Safety | The phase 3 KarMMa-3 trial included 386 patients with triple-class–exposed relapsed and refractory multiple myeloma, who were randomized 2:1 to receive idecabtagene vicleucel (ide-cel; n = 254) or standard regimens (n = 132). Of the patients randomized to ide-cel, 225 ultimately received an ide-cel infusion. The overall response rate (ORR) was significantly higher with ide-cel than with standard regimens (71% vs 42%, respectively), with deeper responses observed in the ide-cel group. Regarding safety, neutropenia occurred in approximately 79% of ide-cel–treated patients and infections in 56%, most commonly respiratory infections. Cytokine release syndrome (CRS) occurred in 88% of ide-cel–treated patients, while neurotoxicity (iiNT/ICANS) was reported in approximately 15%.Deaths occurred in both treatment arms at similar overall rates, predominantly due to disease progression; deaths due to infection were reported in a small proportion of patients in both groups. No new safety signals were identified with longer follow-up. |
| *Kalariya, Hildebrandt et al. 2024* | ORR// PFS, OS, Safety | This retrospective study analysed 156 patients and calculated a frailty score for each patient. Eighteen patients were under 65 years old; the rest were up to 83 years old. The ORR was comparable at 86.7%. CRS occurred frequently in both groups without a significant difference (81.5% in the younger group versus 88% in the older group). ICANS did not differ by age. There were no increased rates of infection-related mortality or treatment discontinuations due to infections, even among older and frailer patients. Ten patients died due to infections. |
| *Minakata, Ishida et al. 2023 ^7^* | ORR, DOR// Safety | This analysis assesses the response and safety of nine Japanese patients in the Phase 2 KarMMa trial. The ORR was 89%. Infections occurred in 56% of patients (grade 3/4 in 11%), with influenza in 22% of patients, and CMV, otitis externa and upper respiratory tract infection in 11% of patients. |
| *Gaballa, Puglianini et al. 2025* | Safety// efficacy, survival | BCMA bispecific antibodies are being analysed in 10 patients with proven CNS involvement. An ORR of 80% is achieved with 100% CNS response. No unmanageable toxicity occurred. Infection occurred in 50% with 1 CMV and HH6 infection. |
| *Ferreri, Hildebrandt et al. 2023* | ORR// DOR, PFS, Safety | This retrospective, multicentre study analysed the efficacy and safety of ide-cel in 50 patients who had previously received BCMA therapy. An ORR of 74% was detected after ide-cel pretreatment versus 88% without prior treatment. The risk of infection was 34% versus 39% in patients with prior BCMA therapy. |
| *Dima, Rashid et al. 2024* | ORR// CR, Safety, PFS, OS | Real-world data from 69 patients with impaired kidney function were analysed to evaluate the efficacy and safety of ide-cel in this group. An ORR of 93% was achieved, with 81% experiencing cytokine release syndrome (CRS) and 28% experiencing immune-related adverse events (IRAE). Infections occurred in 38% of patients receiving SOC versus 49% of patients receiving CAR-T therapy within eight weeks of infection. The dominant infections were bacterial and viral, with a few cases of fungal infection. |
| *Sidana, Ahmed et al. 2025* | ORR, CR// PFS, OS, Safety | A total of 821 patients were included in the real-world analysis, which evaluated the efficacy and safety of ide-cel. An ORR of 73% with a CR rate of 25%) was achieved through the use of CRS. 80% of patients experienced a CR and 45% experienced infections. Secondary malignancy occurred in 4% of patients. |
| *Trando, Ghamsari et al. 202* | ORR// PFS, OS, Safety | This single-centre retrospective analysis included 25 patients, examining efficacy and safety in a real-world setting. Of these patients, 16% had previously received BCMA therapy. An ORR of 84% was achieved, with CRS and infections occurring frequently, affecting 44% of patients. CMV reactivation occurred in nine out of 19 patients. |
| *Logue, Peres et al. 2022* | Incidence of grade ≥ 3 cytopenia // cumulative infection rate, severity, risk-factors | This multicentre study analysed the first 100 days following infusion. After 30 days, 65% of patients had grade 3/4 cytopenias, compared to 40% after 90 days. G-CSF was administered in 88% of cases and an autologous stem cell boost in 8%. Thirteen per cent of patients received immunoglobulins. Infections occurred in 54% of patients, 23% of which were severe. |
| *Kikuchi, Watanabe et al 2025* | Incidence of CMV retinitis// treatment and outcome | This analysis examined 53 Japanese patients with myeloma who received ide-cel therapy and developed CMV retinitis. CMV reactivation and CMV rhinitis were described as rare complications, affecting one patient in eleven. Monitoring for CMV should be performed alongside an additional ophthalmological consultation if symptoms occur. |
| *Akhtar, Oloyede et al. 2025* | ORR, PFS// OS, CRS, Infection, Cytopenia, Frail patients | This retrospective analysis evaluated patients following ide-cel therapy in a real-world setting. A total of 821 patients were analysed, including 251 aged over 70. Being over the age of 70 was associated with an increased risk of CRS and ICANS. No difference in response was detected. Prolonged cytopenia or clinically significant infection did not differ by age group. However, frail patients experienced significantly more infections (49.6% versus 40.9%). |
| *Caillot, Sleiman et al 2024* | Efficacy// Safety | In a French early access study of ide-cel in RRMM (n=24; median 4 prior therapies; follow-up 15.2 months), 92% achieved ≥PR and 70% achieved ≥CR after 6 months, MRD negativity (10⁻⁶) was 75%, median PFS was 14.8 months, and OS was not reached. CRS occurred in 96% (grade ≥3: 4%), ICANS in 12.5% (grade 3: 4%); grade 4 neutropenia in 58%. Infections were common (79%: viral 79%, bacterial 21%, fungal 12.5%), mostly mild; no infection-related deaths. Hypogammaglobulinaemia with IVIG substitution affected 88% of patients. Early CMV viraemia (17%) and BK cystitis (29%) were predominant, COVID-19 was mild. Prophylaxis data (PJP, antiviral, fungal, bacterial) were not reported. CAR-T expansion > 180/µl correlated with longer PFS. |
| *Sanoyan, Seipel et al. 2023* | Infection// risk factors, PFS, OS, IVIG | In the study a total of 47 infections occurred in 55 patients after Ide-cel therapy (53% affected), most of which were mild to moderate and respiratory (68%). There were 40% bacterial, 53% viral (all respiratory), and 6% fungal infections; no CMV, HSV/VZV, or PJP cases. One fatal case of Aspergillus pneumonia occurred. Hypogammaglobulinaemia was common (76% after 1 year) and persistent. The median time to the first infection was 3.9 months, with more than half of the infections occurring in the first 100 days. |
|  |  |  |
| A summary of the trial is given in the supplemental table. It includes the title of the trial. The primary and secondary  endpoints are also given. Abbreviations: Graft-versus-host disease = GVHD; Overall response = ORR; Minimal residual disease = MRD; Cytokine release syndrome = CRS; Immune effector cell neurotoxicity syndrome = ICANS; Progression-free survival = PFS; Overall survival = OS; Complete response = CR; Herpes simplex infection = HSV; Herpes zoster = HZV; Cytomegalovirus = CMV; Pneumocystis jiroveci = PJP; Hepatitis B = HBV; Hepatitis C = HCV. | | |

| Summary Table, Ciltacabtagene autoleucel | | |
| --- | --- | --- |
| Study; type of study | **primary // secondary outcome** | **summary** |
| *Berdeja, Madduri et al. 2021* | ORR// sCR, DOR, PFS, OS, MRD, Safety | The CARTITUDE 1 study, which was conducted as a single arm, investigated the safety and clinical efficacy in 113 patients. An ORR of 97% was achieved. The CRS and neutropenia rates were both 95%. Neurotoxicity occurred in 21% of patients. There were 14 deaths. Infections occurred in 58% of patients, 20% of which were grade 3/4. The most common infections were upper respiratory tract infections, predominantly grade 3/4 pneumonia. |
| *Mi, Zhao et al. 2023* | ORR// CR, DOR, PFS, OS, Safety | 48 patients received cilta-cel in the pivotal Phase II study in China. OR was evaluated. An ORR of 89.6% was achieved. Neutropenia occurred in 97.9%, CRS in 97.9% and infections in 85.4%, of which 37.5% were grade 3/4 infections. The most common infections were pneumonia (35.4%) and respiratory tract infections (33.3%), as well as herpes zoster infections (18.8%). Five patients developed HBV infection. Hypogammaglobulinemia was present in 70%, with a nadir of <400 mg/dl in the first 200 days. Three deaths occurred due to infections |
| *Sidana, Patel et al.2025* | Safety, Efficacy// PFS | Cilta-cel is being evaluated under real-world conditions. Of the 255 patients who underwent leukapheresis, 236 received cilta-cel. Of these patients, 54% would not have met the eligibility criteria for CARTITUDE 1. An ORR of 89% was achieved. Under real-world conditions, infection was the most common cause of death (10%). Infection was detected in 47% of patients (grade 3/4: 46%; bacterial: 41%; viral: 47%; fungal: 3%). Grade 3/4 neutropenia occurred in 80% of patients |
| *San-Miguel, Dhakal et al.2023* | PFS// ORR, CR, MRD, OS, Safety | This Phase 3 study randomised patients between standard therapy and cilta-cel treatment in order to assess PFS. A total of 419 patients were randomised. At a median follow-up period of 15.9 months, 75.9% of patients achieved a PFS of 12 months. The overall ORR differed between the treatment groups: 84.6% in the cilta-cel group and 67.3% in the standard of care (SOC) group. CRS occurred in 76.1% of patients (grade 3/4: 1.1%), with 4.5% experiencing ICANS. Infections occurred in 62% (26.9% grade 3/4) of the cilta-cel cohort and 71.2% (24.5% grade 3/4) of the SOC group. Hypogammaglobulinemia occurred more frequently in the treatment group (90.9% vs. 71.6%). Deaths from any cause were reported in 39 patients in the treatment group and 46 patients in the SOC group. CRS occurred in 76.1% of patients and ICANS in 4.5%. Infections occurred in 62% (grade 3/4: 26.9%) of the cilta-cel cohort, compared to 71.2% (grade 3/4: 24.5%) in the SOC group |
| *Ri, Suzuki et al. 2022* | ORR// VGPR or better, MRD, Safety | The Japanese cohort of the CARTITUDE-1 study is evaluating the efficacy and safety of the treatment. All patients received antiviral, antibiotic, PJP and antifungal prophylaxis. In this subgroup, an ORR of 100% was achieved, with CRS occurring in 88.9% of cases without ICANS. 11.1% of patients experienced an infectious complication. |
| *Htut, Dhakal et al. 2023* | ORR, Safety// sCR, PFS, Os, sever grade of adverse events | This additional analysis evaluated the safety and efficacy of CARTITUDE-1 in patients who had previously undergone allogeneic stem cell transplantation. Seven patients were included in this subgroup. Their safety profile and response were consistent with those of patients without prior allogeneic transplantation, with an infection rate of 57.1%. |
| *Ellithi, Elsallab et al. 2025* | Neurotoxicity and rare adverse events// broader safety profil | This analysis of FAERS reports examines the side effects of commercially available CAR-T products. Of the 4,471,782 entries described, 1,496 were deemed relevant to the therapies. Cilta-cel was associated with facial paralysis and parkinsonism, while patients with ide-cel exhibited confusion and balance disorders. New safety signals emerged in the form of Guillain-Barré syndrome (ROR 17.1) and intracranial haemorrhages (ROR 2.9). Infections occurred, with Haemophilus and CMV infections being particularly notable. Cases of sarcoidosis and cardiac arrhythmias were also reported. |
| *Einsele, San-Miguel et al 2026* | PFS// OS, response rate, MRD, Safety | The updated randomized phase III CARTITUDE-4 trial confirmed superior efficacy of cilta-cel compared with standard of care in lenalidomide-refractory multiple myeloma, with deeper and more durable responses as well as improved PFS and emerging OS benefit. However, infectious complications remained clinically highly relevant.. Infections contributed to treatment related mortality, with four of six treatment-related deaths being infection-related, predominantly within the first year after treatment initiation. High rates of neutropenia and frequent IVIG use are reported. |
| *Dima, Loque et al 2026* | Characterization of cytopenias and infections// Immune reconstitution, risk factors, supportive care, response | In this multicenter retrospective real-world study of 105 RRMM patients treated with cilta-cel, cytopenias and infections were frequent and often prolonged. Grade ≥3 cytopenias occurred in 52% at day 30 and 24% at day 90, while infections affected 49% of patients and were severe in 32%. Viral infections predominated later after treatment. Despite high toxicity rates, cilta-cel showed strong efficacy with a 92% ORR and 63% CR or better. The study highlights the importance of prolonged supportive care, IVIG supplementation, infection prophylaxis, and monitoring of immune recovery after BCMA CAR-T therapy. |
| *Hansen, Dima et al 2026* | ORR, PFS // OS, Safety, treatment-related mortality | This large CIBMTR registry-based real-world study evaluated the safety and efficacy of commercial ciltacabtagene autoleucel (cilta-cel) in 595 heavily pretreated patients with relapsed/refractory multiple myeloma treated between 2022 and 2023. Patients had received a median of 7 prior lines of therapy and 8% had prior BCMA-directed treatment. With a median follow-up of 12 months, ORR was 87%, ≥CR rate was 35%, and 12-month PFS and OS were 73% and 85%, respectively. CRS occurred in 80% (4% grade ≥3), ICANS in 22% (4% grade ≥3), and clinically significant infections in 47% of patients. Most infections were viral (33%) or bacterial (20%), while fungal infections were uncommon (2%). Infection was the leading cause of treatment-related mortality. |
| A summary of the trial is given in the supplemental table. It includes the title of the trial. The primary and secondary endpoints are also given. Abbreviations: Graft-versus-host disease = GVHD; Overall response = ORR; Minimal residual disease = MRD; Cytokine release syndrome = CRS; Immune effector cell neurotoxicity syndrome = ICANS; Progression-free survival = PFS; Overall survival = OS; Complete response = CR; Herpes simplex infection = HSV; Herpes zoster = HZV; Cytomegalovirus = CMV; Pneumocystis jiroveci = PJP; Hepatitis B = HBV; Hepatitis C = HCV. | | |

| Summary Table, all CAR-T cells | | |
| --- | --- | --- |
| Study; type of study | **primary // secondary outcome** | **summary** |
| *Merz, Albici et al. 2025* | *ORR, PFS, CRS, ICANS// NRM, CR* | This analysis compared the efficacy of Ide-Cel (162 patients) and cilta-Cel (42 patients). The ORR was higher in the cilta-Cel cohort (93% versus 79%). The 10-month PFS rate was 82% and the OS rate was 90% in the cilta-cel cohort, compared to 47% and 77% respectively in the ide-cel cohort. Infections caused death in 26% of patients in the ide-cel cohort and 50% of patients in the cilta-cel cohort. cilta-cel was associated with later CRS and longer hospital stays. However, increased use of steroids may further suppress the immune system |
| *Hansen, Peres et al. 2025* | Safety, efficacy // PFS, OS | This retrospective study compares ide-cel and cilta-cel to the standard of care. Of the 641 patients who underwent leukapheresis, 350 were infused with ide-cel and 236 with cilta-cel. cilta-cel patients were more likely to achieve a CR than ide-cel patients (OR 2.42; 95% CI 1.63-3.6). However, cilta-cel patients were also at a higher risk of CRS (OR 6.8), infections (OR 2.03), and delayed neurotoxicity (OR 20.07). There was no difference in the overall incidence of CRS, ICANS or cytopenias, or in non-relapse mortality. |
| *Xu, Wang et al. 2024* | CRS, ICANS, NRM // hospitalization, monitoring, long-term outcome | This multicentre retrospective analysis evaluated the early toxicity profile and early non-relapse mortality in 157 patients. Of these patients, 129 received a CAR-T infusion: 70% received ide-cel and 30% received cilta-cel. CRS occurred in 80% of patients, and ICANS in 26%. Cytopenia occurred in 44% of patients, with 32–33% experiencing neutropenia and infection. 85% were discharged by day 15 after infusion; 3% were readmitted due to infection. An ORR of 88% was achieved by day 30, falling to 77% by day 90. Five patients died within the first 30 days, one of whom died from sepsis. |
| Wang, Cao et al. 2022 | CRS, ICANS, hematologic toxicity // long-term toxicity, infections, NRM | This single-arm phase II study assessed the overall response of BCMA-CAR-T cells, long-term outcomes and safety in 69 patients. An ORR of 92% was achieved. 95% experienced CRS and 11% experienced ICANS. B-cell aplasia, hypogammaglobulinemia, and infections were reported as late adverse events. Thirty per cent had persistent B-cell aplasia beyond three months, 42% had hypogammaglobulinemia, and 41% had infections (30% grade 3/4). Herpes zoster infection and repeated diarrhoea occurred in 5% of patients, and lung infections occurred in 23%. Eighteen patients died |
| *Merz, Gagelmann et al. 202* | ORR, CR, PFS// Safety | The study ‘Remission conversion drives outcomes after CAR T-cell therapy for multiple myeloma’ analysed 343 patients from the German DRST registry who were treated with the CAR-T cell therapies id-cel or cilta-cel. The ORR was 82% for ide-cel and 94% for cilta-cel, with higher CR rates (61% vs. 39%) and better 10-month PFS (76% vs. 47%) for cilta-cel. CRS occurred frequently but was mostly mild (ide-cel: 81%, cilta-cel: 85%); ICANS was more common with cilta-cel (25% vs. 15%). The study shows that conversion to deep remissions is crucial for long-term outcomes. Cilta-cel was overall more effective with comparable safety, which could support its preferred use in everyday clinical practice. |
| *Hill et al, 2024* | Anti-spike IgG titers post-second vaccine dose//Neutralizing antibody level and adverse events | This study analyses the optimal timing of haematological therapies. It was conducted on 466 patients after AlloTX and 65 patients after CAR-T. There was no significant difference in humoral and cellular responses between the 0–4 month and 4–12 month groups. A T-cell-directed response was observed in 57% of patients after alloTX, 83% after autoTX, and 58% after CAR-T cell therapy. |
| *Rejeski, Hansen et al 2023* | CAR-HEMATOTOX score and incidence/severity of hematologic toxicity// infectious complications, prolonged cytopenia, PFS, OS, non-relapse mortality | Rejeski et al. (2023) evaluated the CAR-HEMATOTOX (HT) score as a predictive tool for toxicity and treatment response in patients receiving BCMA-directed CAR-T cell therapy for relapsed or refractory multiple myeloma (RRMM). The study demonstrated that a high HT score was strongly associated with increased rates of severe hematologic toxicity, infections, prolonged cytopenias, and non-relapse mortality (NRM). Moreover, it was found to correlate with inferior PFS and OS. This supports the utility of the HT score in identifying high-risk patients before CAR-T infusion, enabling risk-adapted supportive care strategies. |
| *Portuguese, Liang et al. 2025* | Toxicity// OSS, PFS, OS, time to toxicity, impact of EMD | The study by Portuguese et al. (2025) analyses the influence of extramedullary disease (EMD) on the tolerability of BCMA-CAR-T cell therapies in patients with multiple myeloma. In a retrospective cohort, patients with EMD were found to suffer significantly more severe side effects, particularly a higher incidence of cytokine release syndrome (CRS), ICANS (neurotoxic complications) and infections. Despite comparable ORR, patients with EMD had shorter PFS and OS, as well as a lower CR rate. Patients with EMD show higher therapy-related morbidity. |
| *Oliver-Caldés, González-Calle et al. 2023* | ORR at day 100, CRS and ICANS at day 30// DOR, PFS, OS | This study describes a Spanish BCMA CAR-T product, examining the responses of 30 patients to a fractionated application. A response was achieved in 100% of patients within the first 100 days. CRS was reported in 80% of patients, haematotoxicity in 67%, and the same number of patients experienced infections. Fractional administration and the booster led to a favourable response and potentially reduced acute toxicity. CRS occurred in 80% of patients, but did not reach grade 3 /4. |
| *Mailankody, Matous et al. 2023* | Safety// ORR, DOR | This analysis evaluates the safety and tolerability of the ALLO-715 anti-BCMA CAR-T cell therapy. The aim is to minimise graft-versus-host disease (GVHD). The study included 43 patients with relapsed and refractory myeloma. CRS occurred in 55.8% of cases, neurotoxicity in 14%, and infections in 53.5%. The ORR was 55.8%. |
| *Ravi, Richard et al. 2025* | Safety// ORR, CR, DOR | This phase 1 study is analysing the BCMA-targeted CAR-T product. It is manufactured using an optimised NEX-T process, which shortens production times. The aim is to produce more central memory cells with higher functionality. The analysis included 65 participants. A dose escalation was carried out in two phases. CRS occurred in 82% of participants, ICANS in 8%, and infections in 32% (grade 3/4 in 5% of cases). An ORR of 95% was achieved. Immunophenotypic analysis revealed a higher proportion of central memory T cells |
| *Zhao, Wang et al. 2022* | Safety// Efficacy | This evaluation reports on the safety and, secondarily, the efficacy of the Phase 1 LEGEND-2 study conducted in China after 4 years of follow-up in 74 patients. The BCMA CAR-T product contains two BCMA binding domains and is administered in a fractionated manner. The ORR was 87.8%. CRS occurred in 91.9% of patients. Infections occurred within 6 months of treatment, including 4 viral infections. |
| *Xu, Wang et al. 2024* | Safety// Efficacy | This analysis describes the long-term (5 years) observation of autologous BCMA-CAR-T cell LCAR-B38M. The dosage was 0.5 x 10⁶ cells/kg, administered as either single or divided fractionated infusions. A total of 74 patients were enrolled in the study, achieving a 5-year PFS rate of 21% and an OS rate of 49.1%. Infections were observed over a period of more than six months, without severe cases. |
| *Xia, Sun et al 2025* | ORR// PFS, Safety | Single-arm phase 2 salvage study from China: In 37 MM patients with progression after anti-BCMA CAR-T, anti-GPRC5D CAR-T achieved an ORR of 84% (≥CR 35%; MRD-negative 54%) after a median follow-up of 12.6 months. Follow-up; median PFS 4.5 months. CRS occurred in 70% (grade 3: 5%, no grade 4) and ICANS in 3% (grade 1 only); haematological toxicities were common (neutropenia grade 3/4: 78%). Infections occurred in 51% of patients (predominantly respiratory; 11% fungal). There were no treatment-related deaths. |
| *Frenking, Zhou et al 2024* | Predictive value of EASIX// PFS and OS in association with EASIX | A retrospective multicentre analysis (n = 129) of ide-cel in relapsed/refractory multiple myeloma (RRMM) showed that a higher EASIX score prior to lymphodepletion or on day 0 was associated with severe late haematotoxicity (late ICAHT), delayed neutrophil recovery, late severe infections and more frequent ICANS. It was also associated with shorter progression-free survival (PFS) and poorer overall survival (OS). The median PFS was 8.6 months and the OS had not yet been reached at the time of follow-up (9.6 months). CRS occurred in 84% of patients (grade 3: 1%), and ICANS occurred in 9% of patients (grade 3: 2%; grade 4: 1%). Late ICAHT grade ≥3 occurred in 21% of evaluable patients, and late severe infections (CTCAE grade ≥3) occurred in ~13%. |
| *Reyes, Huang et al 2023* | CRS, ICANS, Hypogammaglobulinemia, Infection, Neutropenia// OS, PFS, MRD negativity | This retrospective, single-centre study investigated the safety and efficacy of BCMA CAR-T cell therapy in older (≥70 years) vs. younger (<70 years) patients with similar characteristics (n 83). Toxicities occurred at similar rates (CRS 77% vs. 78%; ICANS 9% vs. 13%; median ANC recovery 12.5 vs. 13 days; Hypogammaglobulinaemia: 82% v.s. 72%; infections: 36% vs. 52%). The ORR was 82% in patients aged ≥70 years vs. 89% in patients aged <70 years, the ≥CR was 59% vs. 54% and the MRD negativity was 50% vs. 59%. The median PFS was 13.1 months for patients aged ≥70 years and 12.5 months for patients aged <70 years. The OS was not reached for patients aged ≥70 years and was 31.4 months for patients aged <70 years. There was no significant age difference after multivariate analysis. |
| *Wang, Li et al 2021* | Humoral immunodeficiency | In the retrospective multicentre study, 40 patients with relapsed/refractory multiple myeloma were examined after anti-BCMA CAR T-cell therapy to characterize humoral immune reconstitution and infection risks. There was marked hypogammaglobulinaemia of all immunoglobulin classes with recovery after one year in IgM 73%, IgG 53% and IgA only 24% (median for IgA not reached). Overall, 57.5% of infections occurred – predominantly bacterial and respiratory – but no infection-related deaths. Low IgG levels correlated with severe infections. |
| *Wesson, Dima et al. 2024* | Toxicity and non-relapse mortality // Side effect | In this multicentre retrospective cohort study, 129 patients who underwent CAR-T cell therapy for multiple myeloma were analysed. CRS occurred in 80%, ICANS in 26% and persistent cytopenia in 44%. Infections were observed in about one third of patients (32–33%), mostly within the first 100 days after infusion. Early mortality was 1.1% (mainly due to infection or HLH), with a further 1.2% dying in the first year, mainly from infections. Approximately one quarter of patients required inpatient monitoring for up to four weeks after therapy; the median length of hospitalisation was 15 days. The objective response rate was 88% by day 30. The study showed that most toxicities occurred early and that infection-related mortality accounted for the largest proportion of NRM. |
| *Thiruvengadam, Sheng et al. 2022* | Infection// risk factors, PFS, OS, blood cell count | 12 months after BCMA-CAR-T in RR myeloma: 47 infections in 53% (53% viral, 40% bacterial, 6% fungal), mostly mild–moderate and respiratory; one fatal Aspergillus infection. Frequency highest in the first 100 days (median time to first infection 3.9 months). Early severe neutropenia almost universal, hypogammaglobulinaemia common and persistent (76% after 1 year; 58% received IVIG). No clear statistical risk factor identified; trends: >3 prior therapies, bridging, recent infection, post-CAR-T lymphopenia. Overall, few severe cases (9%); no detectable influence of ‘infection yes/no’ on OS. |
| *Josyula, Pont et al. 2022* | Infection// risk factors for infection | The study examined 32 patients with relapsed/refractory multiple myeloma after BCMA-CAR-T therapy. Almost all developed severe cytopenia and hypogammaglobulinaemia (IgG < 400 mg/dL). Even before therapy, pathogen-specific immune protection was often lacking, and this continued to decline after treatment. Infections occurred in 53% of patients, mostly mild viral infections, while severe infections occurred predominantly in the first 28 days and in patients with a high CRS score. |
| *Little, Tandon et al. 2023* | Infection// Severity, risk factors | Retrospective 2-centre analysis of infections following BCMA-CAR-T in RRMM (n=99; median 6 prior therapies, follow-up 12 months): 37% suffered 64 infections—early (day 0–100) mainly bacterial (69%; BSI/UTI/pneumonia), late (day 101–365) mainly viral (41%; predominantly respiratory infections including COVID-19); no late invasive fungal infections occurred, a total of 2 probable early aspergillosis cases, 1 Candida peritonitis; 1 infection-related death. Hypogammaglobulinaemia (IgG ≤ 400 mg/dL) in 70%; IVIG in 63% (first dose median day 70). Prophylaxis: continuous antiviral (acyclovir/valacyclovir), PJP until at least day 180, some antibacterial until cell recovery, inconsistent antifungal. Risk factors for late respiratory infections: early respiratory infection (HR 2.95) and hypogammaglobulinaemia d30–100 (HR 6.06). |
| *Richardson, Schütte et al. 2025* | Infection // risk factor, prophylaxes | In this multicentre German real-world study on BCMA-CAR-T in RRMM (n = 80; median 6.5 prior therapies), infections occurred in 71% of patients – predominantly viral (63%, including COVID-19 17%), followed by bacterial 35% and fungal 5%. The most common sites of infection were the respiratory tract (53%) and the bloodstream (12%). Infection-related mortality was 7%. Hypogammaglobulinaemia was frequently observed and treated with IVIG. Prophylaxis included valacyclovir + TMP-SMX for 6 months, no standard bacterial or antifungal prophylaxis. Median onset of infection: 38 days. CRS 90% (grade ≥ 3: 6%), ICANS 13% (grade 3: 1%), neutropenia 44%. |
| *Little, Pena et al 2025* | Incidence and characteristics of invasive fungal disease | This retrospective two-center cohort study evaluated the incidence of invasive fungal disease (IFD) in 234 patients with relapsed/refractory multiple myeloma treated with BCMA-directed CAR T-cell therapy between November 2016 and October 2023. The 1-year cumulative incidence of IFD was low at 1.7%, with only four cases identified (three invasive aspergillosis and one *Candida albicans* peritonitis). All patients who developed IFD had experienced significant CAR T-cell–related toxicities (CRS and/or ICANS), received high-dose corticosteroids and additional immunosuppressive therapies, and most had high CAR-HEMATOTOX scores. Two patients recovered after antifungal treatment, whereas two died with concomitant IFD. |
| *Yan, Du et al 2026* | MRD negativity// CR, Safety | The phase II CAREMM-001 study evaluated frontline BCMA-directed CAR T-cell therapy in patients with newly diagnosed multiple myeloma (NDMM) who were ineligible for or not proceeding to autologous stem-cell transplantation. Patients received induction therapy followed by BCMA CAR T-cell infusion, consolidation, and lenalidomide maintenance. Among 36 infused patients, the MRD negativity rate reached 100%, with no MRD recurrence observed during follow-up. Deep responses improved over time, with the complete response rate increasing from 33.3% before infusion to 94.4% at last follow-up. The safety profile was manageable: CRS occurred in 52.8% of patients (all grade 1–2), ICANS in 5.6% (all grade 1), and infections in 30.6% of patients, including grade ≥3 infections in 19.4%. No disease progression or deaths were reported at the data cutoff. |
| *Cohen, Lebel et al 2026* | Incidence of viral reactivations // characteristics and risk factors of viral reactivation | This single-center study evaluated the incidence and clinical significance of viral reactivations in 75 patients with relapsed/refractory multiple myeloma treated with anti-BCMA CAR T-cell therapy (HBI0101). The primary focus was on CMV, EBV, and adenovirus reactivation. CMV reactivation occurred in **20%** of patients and EBV reactivation in **8%**, whereas no adenovirus reactivations were observed. Most CMV reactivations (87%) were asymptomatic and clinically insignificant. Neither CMV nor EBV reactivation was associated with progression-free survival or overall survival. Most clinically significant CMV reactivations occurred after disease progression and during subsequent immunosuppressive therapies rather than being directly attributable to CAR T-cell treatment. The authors concluded that routine CMV surveillance in asymptomatic patients may not be necessary and that a risk-adapted monitoring strategy may be more appropriate. |
| *Habib, Ahmed et al 2026* | Safety and Efficacy | This multicenter real-world study evaluated the safety and efficacy of BCMA-directed CAR-T therapy (ide-cel and cilta-cel) in 223 patients with RRMM, including 25 patients with baseline renal impairment (CrCl <45 mL/min). Response rates, PFS, and OS were comparable between groups, demonstrating that renal impairment did not adversely affect CAR-T efficacy. However, patients with renal impairment experienced significantly higher rates of ICANS (60% vs. 19%) and infections requiring hospitalization (44% vs. 20%). The authors concluded that BCMA CAR-T therapy remains feasible and effective in patients with renal impairment, although enhanced monitoring for neurotoxicity and infections is warranted. |
| *Filippator, Ntanasis-Stathopoulos et al 2026* | OS // PFS, Safety | This retrospective real-world study analyzed 1,272 patients with relapsed/refractory multiple myeloma (RRMM) treated with the approved BCMA-directed CAR T-cell therapies ciltacabtagene autoleucel (cilta-cel) or idecabtagene vicleucel (ide-cel) using the TriNetX database. Both therapies demonstrated favorable survival outcomes in routine clinical practice, with 12-month overall survival rates of 89.6% for cilta-cel and 86.0% for ide-cel. Prior exposure to anti-CD38 antibodies did not significantly affect outcomes, whereas impaired renal function was associated with poorer survival. Cytokine release syndrome (CRS) and ICANS were common but severe events were rare. Hematologic toxicities, infections, and hypogammaglobulinemia were frequent, highlighting the need for long-term monitoring. |
| *Liu, Zhang et al 2026* | Hypogammaglobulinemia // Infection, risk factors, mortality | Liu et al. conducted a retrospective study of 147 patients with relapsed/refractory multiple myeloma treated with BCMA-directed CAR-T therapy to evaluate hypogammaglobulinemia, infections, and mortality. Hypogammaglobulinemia was common and increased after CAR-T therapy, affecting 80% of patients, while 29.9% developed severe infections and 25% required hospitalization for infection. Pre-existing hypogammaglobulinemia was associated with a significantly increased risk of post-CAR-T infections, and lower IgG levels were linked to higher mortality. The study highlights persistent immune dysfunction and identifies hypogammaglobulinemia as an important risk factor for infection and adverse outcomes following BCMA CAR-T therapy |
| A summary of the trial is given in the supplemental table. It includes the title of the trial. The primary and secondary endpoints are also given. Abbreviations: Graft-versus-host disease = GVHD; Overall response = ORR; Minimal residual  disease = MRD; Cytokine release syndrome = CRS; Immune effector cell neurotoxicity syndrome = ICANS; Progression-free survival = PFS; Overall survival = OS; Complete response = CR; Herpes simplex infection = HSV; Herpes zoster = HZV; Cytomegalovirus = CMV; Pneumocystis jiroveci = PJP; Hepatitis B = HBV; Hepatitis C = HCV. | | |

| Summary Table, bispecific and CAR-T cells | | |
| --- | --- | --- |
| Study; type of study | **primary // secondary outcome** | **Summary** |
| *Nath, Shekarkhand et al. 2024;* | Incidence of severe infections// risk factors | This retrospective study compares BCMA products. The analysis included 92 CAR-T cell patients, 55 patients with bispecific antibodies, and 109 patients with antibody-drug conjugates. Infections were observed in 40% of patients receiving bispecific BCMA antibodies, 26% of CAR-T patients, and 8% of ADC patients. Deaths due to infection occurred only in patients receiving bispecific antibodies (7%). Patients receiving bispecific antibodies experienced more severe infections with a faster onset compared to CAR-T patients. Patients with CAR-T show a lower risk of infection in cases of hypogammaglobulinaemia and neutropenia compared to bispecific antibody patients. |
| *Howard, Concepcion et al. 2024* | Frequency and nature of unscheduled healthcare interactions// comparison between bispecific and CAR-T | This retrospective study analysed 46 patients receiving BCMA CAR-T or bispecific antibodies according to the frequency and cause of unscheduled contacts. A total of 89% experienced an unscheduled admission, with an average of 3.7 admissions per patient. Patients receiving bispecific antibodies visited the emergency department 1.8 times more frequently. Mild respiratory infections were a common cause of unplanned presentations, particularly among patients receiving bispecific antibodies, and to a lesser extent among CAR-T recipients. |
| *Hammons, Haider et al. 2024* | ORR, MRD, safety // CRS, ICANS, GVHD | This retrospective cohort analysis examined the further treatment of patients with myeloma after alloTX, comparing CAR-T and bispecific antibody therapy. An ORR of 92% was observed with CAR-T therapy, compared to 44% with bispecific antibody therapy. Grade 3 side effects were reported in 92% of patients receiving CAR-T therapy and 56% of those receiving bispecific antibody therapy. CRS occurred in 83% of patients receiving CAR-T therapy and in 78% of those receiving bispecific antibody therapy. Neurotoxicity occurred in three CAR-T patients, none of which were severe. Grade 3/4 infections occurred in 50% of patients receiving CAR-T therapy and 44% of patients receiving bispecific antibody therapy. No deterioration of GVHD was detected following any of the therapies. |
| *Lesokhin, Nath et al 2024* | Safety// Infections | In this single-centre retrospective study involving 256 RRMM patients (CAR-T n=92, BsAb n=55, ADC n=109), severe infections (grade ≥3) occurred more frequently with BCMA bispecifics (40%) than with CAR-T (26%) or ADC (8%); fatal infections were only observed with BsAb (7%). With CAR-T, infections were more frequent in the first 100 days, while the risk remained persistent with BsAb (after 1 year: CAR-T vs. BsAb IRR 0.43). Hypogammaglobulinaemia was more strongly associated with infections with BsAb; neutropenia was more frequent after CAR-T and correlated with early infections. Most infections were respiratory bacterial/viral infections; CMV/PJP were rare. |
| *Mersi, Bruow et al 2026* | Characteristic of infections | A single-center retrospective cohort study including 137 patients with multiple myeloma treated with BCMA-directed CAR T-cell therapy, BCMA-targeting bispecific antibodies (BsAbs), or GPRC5D-targeting BsAbs found that infections were common across all treatment groups, with most being low grade but often prolonged and recurrent. Severe infections occurred more frequently in patients receiving CAR-T therapy and BCMA-directed BsAbs, whereas GPRC5D-directed BsAbs showed a more favorable infection profile. The findings highlight the substantial infectious burden associated with T-cell–redirecting therapies and emphasize the importance of infection prevention and immunoglobulin supplementation. |
| A summary of the trial is given in the supplemental table. It includes the title of the trial. The primary and secondary endpoints are also given. Abbreviations: Graft-versus-host disease = GVHD; Overall response = ORR; Minimal residual disease = MRD; Cytokine release syndrome = CRS; Immune effector cell neurotoxicity syndrome = ICANS; Progression-free survival = PFS; Overall survival = OS; Complete response = CR; Herpes simplex infection = HSV; Herpes zoster = HZV; Cytomegalovirus = CMV; Pneumocystis jiroveci = PJP; Hepatitis B = HBV; Hepatitis C = HCV. | | |
